# The Effect of GLP-1 Agonists on Patients with Metabolic-Associated Steatotic Liver Disease: A Systematic Review and Meta-Analysis

**DOI:** 10.3390/pharmaceutics18010086

**Published:** 2026-01-09

**Authors:** Denisia Adelina Tornea, Christian Goldis, Alexandru Isaic, Alexandru Catalin Motofelea, Alexandra Christa Sima, Tudor Ciocarlie, Andreea Crintea, Razvan Gheorghe Diaconescu, Nadica Motofelea, Adrian Goldis

**Affiliations:** 1 Doctoral School, “Victor Babes” University of Medicine and Pharmacy Timisoara, 300041 Timisoara, Romania; denisia.tornea@umft.ro; 2 Department of Gastroenterology and Hepatology, “Victor Babeș” University of Medicine and Pharmacy, 300041 Timisoara, Romania; 3 Faculty of Medicine, “Victor Babeș” University of Medicine and Pharmacy, 300041 Timisoara, Romania; 4 Department X of General Surgery, “Victor Babes” University of Medicine and Pharmacy, 300041 Timisoara, Romania; isaic.alexandru@umft.ro; 5 Centre for Molecular Research in Nephrology and Vascular Disease/MOL-NEPHRO-VASC, “Victor Babes” University of Medicine and Pharmacy, 300041 Timisoara, Romania; alexandru.motofelea@umft.ro (A.C.M.); sima.alexandra@umft.ro (A.C.S.); 6 Department of Diabetes, Nutrition and Metabolic Diseases Clinic, “Pius Brînzeu” Emergency Clinical County University Hospital, 300723 Timisoara, Romania; 7 Department VII Internal Medicine II, Discipline of Cardiology, University of Medicine and Pharmacy “Victor Babes”, 300041 Timisoara, Romania; ciocarlie.tudor@umft.ro; 8 Department of Molecular Sciences, University of Medicine and Pharmacy “Iuliu Hațieganu”, 400349 Cluj-Napoca, Romania; 9 Department of Oncology, OncoHelp Hospital Timisoara, Ciprian Porumbescu Street, No. 59, 300239 Timisoara, Romania; 10 Department of Obstetrics and Gynecology, “Victor Babes” University of Medicine and Pharmacy Timisoara, Eftimie Murgu Square No. 2, 300041 Timisoara, Romania; 11 Department of Clinical Practical Skills, “Victor Babes” University of Medicine and Pharmacy Timisoara, 300041 Timisoara, Romania

**Keywords:** liver steatosis, GLP-1 receptor agonists, liver fibrosis, weight loss, glycosylated hemoglobin, liver fibrosis

## Abstract

**Background**: Metabolically associated fatty liver disease (MASLD) constitutes a major burden. Glucagon-like peptide-1 agonists (GLP-1) could improve hepatic steatosis as well as weight loss. However, the effect of GLP-1 agonists on patients with and without diabetes and the effect of newer drugs (dual and triple agonists) are unclear. **Objective**: To investigate the effect of GLP-1 agonists, including dual and triple agonists, in patients with metabolic-associated liver steatosis and steatohepatitis, while exploring their effect on patients with and without type 2 diabetes. **Methods**: We searched PubMed, Scopus, and Web of Science in October 2025 for randomized parallel controlled trials that investigated the effect of GLP-1 agonists in patients with MASLD or metabolic-associated steatohepatitis (MASH). We assessed the quality of the included studies using Cochrane ROB2. We performed the analysis using RevMan 5.4. We performed subgroup analysis based on the status of diabetes, the control group, and the class of GLP-1 agonist (single, dual, or triple). **Results**: We included twenty studies. Compared to the control group, GLP-1 agonists were associated with a statistically significant increase in the resolution of MASH without worsening fibrosis (RR 3.03, *p* < 0.0001) and at least one stage of liver fibrosis without the worsening of MASH compared to the control group (RR: 1.45, *p* < 0.00001). GLP-1 agonists were associated with a statistically significant weight reduction (SMD −1.11, *p* < 0.0001), glycosylated hemoglobin (SMD −0.81, *p* < 0.00001), levels of aspartate aminotransferase (SMD −0.48, *p* = 0.008), and alanine aminotransferase (SMD −0.54, *p* = 0.008). However, in patients without type 2 diabetes, GLP-1 agonists had no significant effect on weight loss (SMD −0.97, *p* = 0.12) or improvement in fibrosis (RR 1.54, *p* = 0.24). There was a statistically significant increase in the overall adverse events (RR 1.10, *p* < 0.00001), while there was no significant difference in serious adverse events (*p* = 0.35). **Conclusions**: GLP-1 agonists improved liver fibrosis, steatohepatitis, weight loss, HbA1c, and liver enzymes in patients with MASLD or MASH. Overall, GLP-1 agonists were associated with a significantly higher risk of adverse events compared to the control, while serious adverse events were comparable between both groups. There was no significant effect on weight loss or improvement in fibrosis in patients without type 2 diabetes. However, there was a limited number of studies in this population. Thus, further research is needed before recommendations can be made for this subgroup.

## 1. Introduction

Metabolic dysfunction-associated fatty liver disease (MASLD), previously known as non-alcoholic fatty liver disease, represents a major burden, affecting up to 34.2% of the population globally [1]. Metabolic dysfunction-associated fatty liver disease is defined as the presence of hepatic steatosis along with metabolic risk factors, including obesity, type 2 diabetes, and hyperlipidemia [2]. This could be further complicated by steatohepatitis, liver fibrosis, and cirrhosis. Indeed, it is a major cause of liver cirrhosis and hepatocellular carcinoma [3]. MASLD is the second leading cause of liver transplants and is associated with significant morbidity and mortality rates [3,4]. Thus, early management and control of the disease is essential.

The pathophysiology of MASLD is driven by weight gain and insulin resistance; thus, the current treatment options focus on addressing these two factors [5]. The main management option for MASLD is weight loss through lifestyle interventions. Weight reduction ≥5% could reduce hepatic steatosis, while a reduction of 7–10% could reduce inflammation, and ≥10% could improve fibrosis [6]. However, Gomez et al. reported that less than a third of the participants could achieve weight loss of more than 5% through lifestyle interventions [6]. Moreover, the long-term sustainability of weight loss is limited, as most benefit is achieved at 6 months, with partial weight regain at one-year and two-year follow-ups, highlighting the challenges of long-term adherence to lifestyle interventions [7]. Thus, there is a need for other therapeutic strategies.

Glucagon-like peptide 1 receptor agonists (GLP-1) are incretin-based therapies that increase insulin release and reduce gastric emptying. They are used in the management of diabetes as well as obesity; they improve weight loss, increase satiety, and reduce insulin resistance and inflammation [8]. Thus, they could improve hepatic steatosis and reduce the progression of liver fibrosis. Furthermore, dual agonists that work through activating both GLP-1 agonists and glucose-dependent insulinotropic polypeptide (GIP) could provide superior results in weight reduction through the synergistic action [8,9]. Thus, they could provide promising results in patients with MASLD or MASH. However, their effect is yet to be determined.

While several systematic reviews [10,11,12] have investigated the effect of GLP-1 agonists, there are still research gaps. For instance, Liu et al. [10] investigated the effect of incretins; however, outcomes such as MASH resolution or improvement of fibrosis were not investigated in patients receiving GLP-1 agonists only. On the other hand, the recent meta-analysis by Mantovani et al. examined the effect of GLP-1 agonists on these outcomes in patients with confirmed hepatic steatosis through MRI or biopsy. However, they excluded the studies involving newer dual or triple GLP agonists [11].

Thus, the effect of newer GLP-1 agonist drugs (dual or triple agonists) was not investigated, although several newly published studies reported a significant reduction in hepatic steatosis [13,14,15,16]. Furthermore, these meta-analyses investigated the effect of GLP-1 agonists regardless of diabetes status [10,11,12]. Thus, while most of their included studies had patients with type 2 diabetes, the effect of GLP-1 agonists on patients without type 2 diabetes is yet to be determined. Thus, we conducted this comprehensive systematic review and meta-analysis to provide an up-to-date investigation of the efficacy of GLP-1 agonists, including dual and triple agonists, in patients with metabolic-associated liver steatosis and steatohepatitis, while focusing on the effect on both patients with and without type 2 diabetes.

## 2. Materials and Methods

We conducted this systematic review and meta-analysis according to the Preferred Reporting Items for Systematic Reviews and Meta-Analyses (PRISMA) statement and followed the Cochrane Handbook guidelines [17,18]. The registered version is under registration number CRD420251236298.

### 2.1. Literature Search

We searched Scopus, PubMed, and Web of Science in October 2025 using the following keywords in the search strategy (“Glucagon Like Peptide 1 Receptor Agonists” OR “GLP-1 Agonists” OR “GLP 1 Agonists” OR “GLP-1 Receptor Agonists”) AND (“Non alcoholic Fatty Liver Disease” OR “Nonalcoholic Fatty Liver” OR NAFLD OR “Metabolic-dysfunction associated steatotic liver disease” OR MASLD OR “Fatty Liver” OR NASH OR “Metabolic dysfunction-associated Steatohepatitis” OR MASH OR “liver fibrosis” OR “hepatic steatosis”). The full search strategy is in the Supplementary Materials.

### 2.2. Eligibility Criteria and Study Selection

We included studies written in English with the following PICO criteria: Population (P): patients aged ≥ 18 with metabolic-associated fatty liver disease or steatohepatitis (hepatic steatosis confirmed through MRI or biopsy in addition to metabolic risk factors regardless of baseline fibrosis stage or inflammatory activity); Intervention (I): GLP-1 agonists; Control (C): placebo or standard of care such as lifestyle intervention, insulin, or metformin for T2DM; Outcome (O): resolution of steatohepatitis, improvement in fibrosis, weight loss, liver enzymes, intrahepatic fat, and adverse events; Study design (S): Randomized parallel controlled clinical trials.

We excluded animal studies, studies not available in English, reviews, case reports, abstracts, letters, and non-randomized clinical trials. Additionally, we excluded studies that were conducted on pediatric patients or where the diagnosis was not confirmed through MRI or biopsy. Moreover, we excluded studies that did not include a high proportion of patients (≥95% of patients with MASLD) when separate data for patients with MASLD were not provided.

The results of the literature search were collected in an Excel sheet through two phases. At first, we screened the titles and abstracts of the retrieved studies, and then we screened the full text of the eligible studies.

### 2.3. Data Extraction and Quality Assessment

The authors performed the data extraction using prepared Excel sheets. The extracted data from the included studies were the general characteristics, baseline characteristics, and outcomes. The general characteristics of trials involved the country, sample size, patient inclusion, and exclusion criteria, and follow-up duration. The baseline characteristics of patients included age, gender, proportion of patients with diabetes, and baseline BMI, HbA1C, and liver enzyme levels. The primary outcomes were the resolution of MASH without worsening fibrosis, improvement of at least one stage of liver fibrosis without worsening of MASH, and the change in hepatic fat content.

The secondary outcomes were the change in weight, HbA1C, resolution of MASH with improvement in fibrosis, change in alanine aminotransferase (ALT), aspartate aminotransferase (AST), quality of life, and the incidence of adverse events.

The quality of the included randomized clinical trials (RCTs) was assessed using the Cochrane ROB2 tool [19]. The tool involves seven domains: the risk of bias arising from the randomization process, the risk of bias due to deviation from the intended intervention, the risk of bias due to missing outcome data, the risk of bias in measuring the outcome, and the risk of bias in selecting the reported results. The judgment of each domain was classified as either low risk, some concerns, or high risk of bias.

### 2.4. Statistical Analysis

We performed the analysis using RevMan 5.4 software. We used a fixed-effects model when no significant heterogeneity was detected. Otherwise, we used a random-effects model. Significant heterogeneity was defined as *p* < 0.1. For continuous outcomes, the standardized mean difference (SMD) and its 95% confidence interval (CI) were calculated, while the risk ratio (RR) with 95% confidence intervals (CI) was calculated for dichotomous outcomes. When significant heterogeneity was detected, we attempted to resolve it through a leave-one-out sensitivity analysis. We performed subgroup analysis based on the used GLP-1 agonist, the control group, and type 2 diabetes status. We assessed publication bias through visual inspection of funnel plots. We performed a meta-regression using open meta-analyst software to investigate the effect of weight reduction on GLP-1 agonist-mediated reduction in liver fat content.

## 3. Results

### 3.1. Search Results and Study Selection

**Figure 1:**
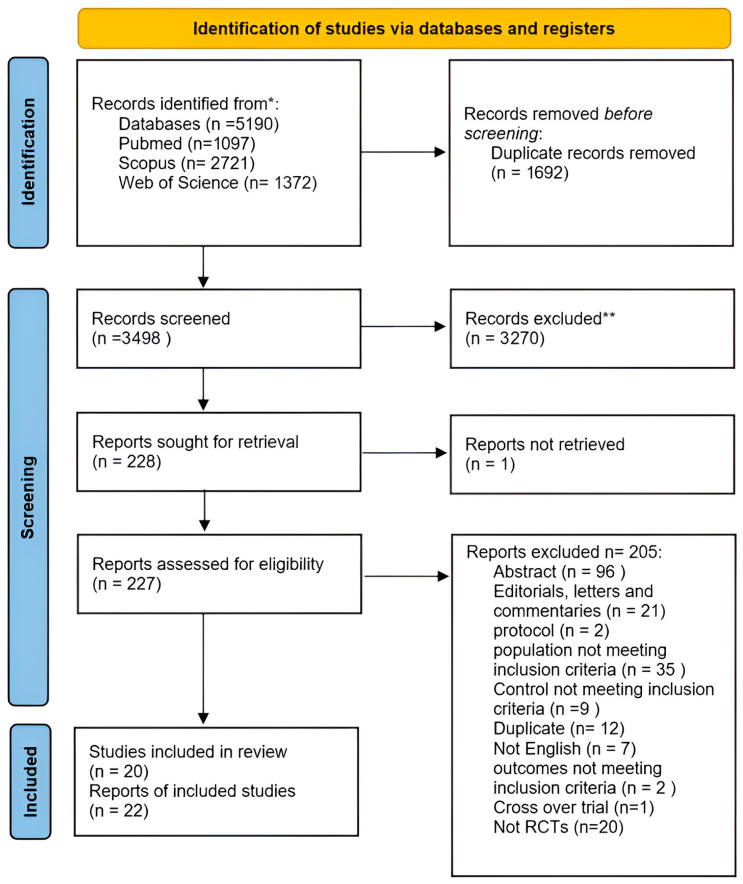
PRISMA flow chart. * Databases searched: PubMed, Scopus, and Web of Science. ** Records excluded based on title and abstract screening.

The literature search revealed 5190 articles, of which 1692 were duplicates (Figure 1). Title and abstract screening were performed on 3498 articles, while 227 articles were screened according to their full text. Finally, 20 studies [13,14,15,16,20,21,22,23,24,25,26,27,28,29,30,31,32,33,34,35], in addition to two reports [36,37] of the studies, were included in this systematic review and meta-analysis.

### 3.2. Characteristics of Included Studies

The baseline characteristics and summary of the included studies are shown in Table 1 and Table 2. The sample size of the included studies ranged from 33 to 800. Eight studies [14,16,20,29,30,31,32,33] included patients with MASH, while four studies [15,25,26,31] included patients without type 2 diabetes. The follow-up duration ranged from 12 to 72 weeks. The mean age of the participants ranged from 33.8 to 65.8 years, and the percentage of males ranged from 34% to 100%. Baseline mean body mass index ranged from 24.5 to 38.6, while baseline mean weight ranged from 65.2 to 110.8 kg.

### 3.3. Quality Assessment

**Figure 2:**
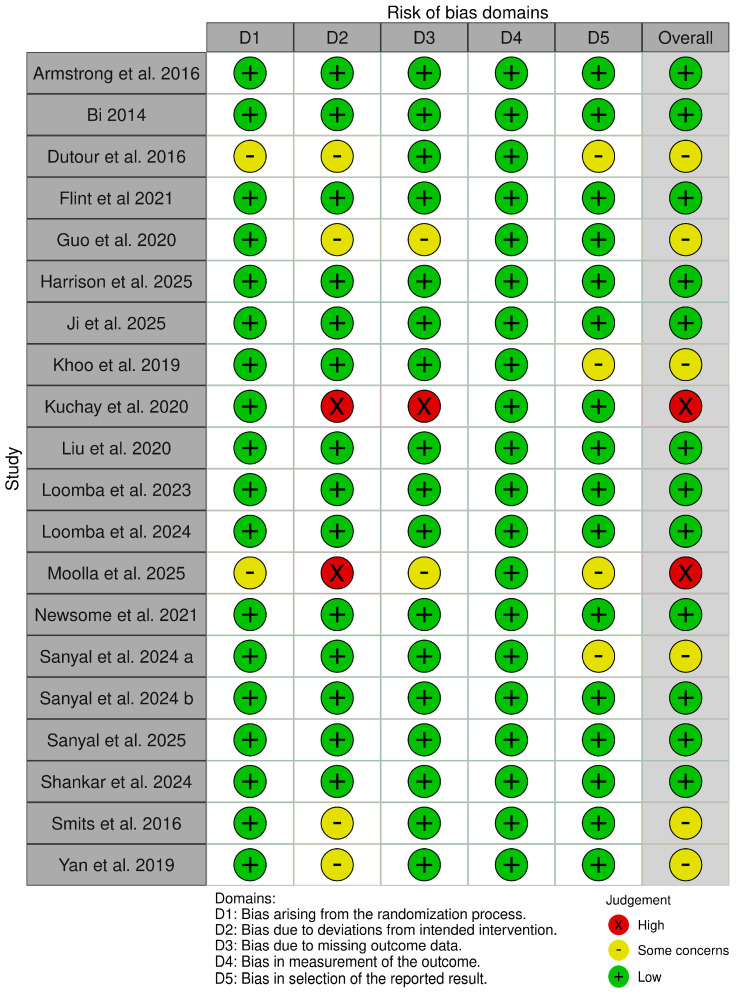
Risk of bias summary [13,14,15,16,20,21,22,23,24,25,26,27,28,29,30,31,32,33,34,35].

**Figure 3:**
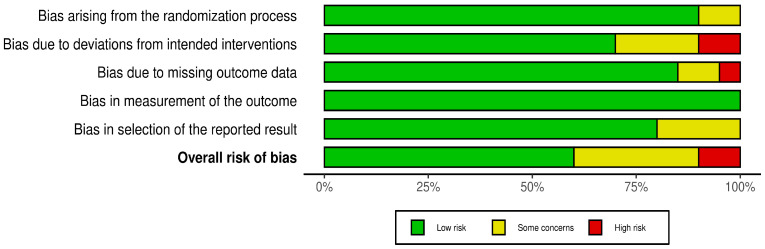
Risk of bias graph.

The risk of bias summary and graph are shown in Figure 2 and Figure 3. Six studies [14,22,24,26,34,35] had some concerns, two studies [27,31] had high concerns, while eleven studies [13,15,20,21,23,25,28,29,30,31,33] had a low risk of bias. Dutour and Moolla et al. had some concerns about bias due to the randomization process, as there was no information about the allocation concealment [22,31]. Smits et al., Yan et al., Guo et al., and Dutour et al. [22,24,34,35] had some concerns about deviations from the intended interventions, while Kuchay and Moolla et al. [27,31] had a high risk of bias. In the bias due to missing outcome data, Moolla et al. and Guo et al. had some concerns [24,31], while Kuchay et al. had high concerns [27]. Dutour et al., Khoo et al., Moolla et al., and Sanyal et al. [14,22,26,31] had some concerns about the risk of bias due to missing outcome data, as there was insufficient information on the analysis plan.

### 3.4. Outcomes

In the following subsections, subgroup analyses comparing single versus dual or triple GLP-1-based agonists, as well as subgroups based on type 2 diabetes status, are presented. These comparisons should be interpreted cautiously, as dual and triple agonists were evaluated in a limited number of recent trials. There was significant heterogeneity detected in some outcomes, including liver fat percentage, weight loss, HbA1c, and liver enzyme levels. Thus, these results should be interpreted cautiously, and statistical significance should not be assumed to reflect a consistency of effects across trials.

### 3.5. Effect on Liver Steatosis, Steatohepatitis, and Liver Fibrosis

**Figure 4:**
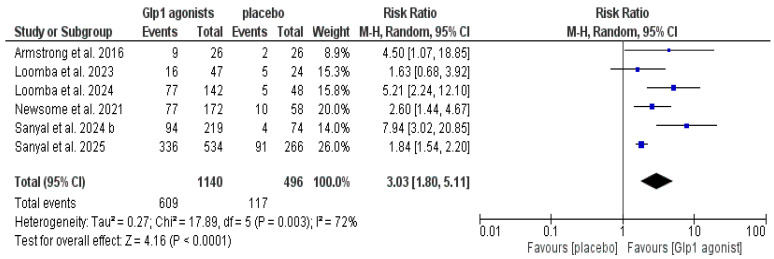
Forest plot of resolution of MASH without worsening in fibrosis in the overall population [14,20,29,30,32,33].

The data showed that GLP-1 agonists were associated with a statistically significant increase in the incidence of resolution of MASH without worsening fibrosis compared to the control group (RR: 3.03, CI [1.80, 5.11], *p* < 0.0001); however, the pooled studies were heterogeneous (*p* = 0.003, I^2^ = 72%), as shown in Figure 4 and Table 3.

**Figure 5:**
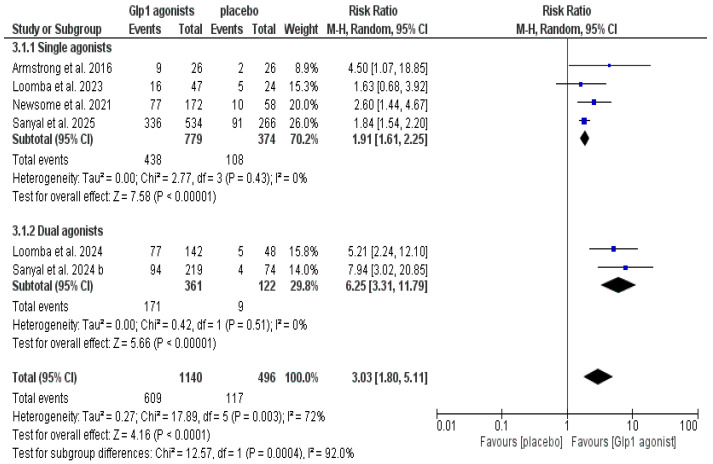
Forest plot of resolution of MASH without worsening in fibrosis in single vs. dual GLP-1 agonists [14,20,29,30,32,33].

Similarly, the same findings were found for both single GLP-1 agonists (RR 1.91, CI [1.61, 2.25], *p* < 0.00001) and dual or triple agonists (RR 6.25, CI [3.31, 11.79], *p* < 0.00001), and the analysis was homogeneous, I^2^ = 0, as shown in Supplementary Table S1 and Figure 5.

**Figure 6:**
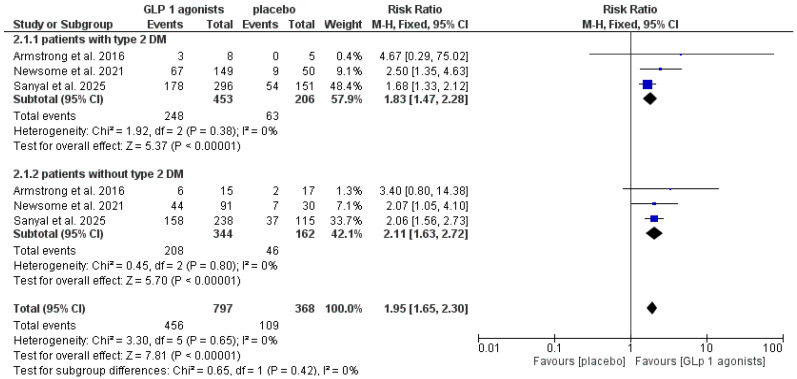
Forest plot of resolution of MASH without worsening in fibrosis in patients with and without type 2 diabetes [20,32,33].

Also, both patients with and without type 2 diabetes had a statistically significant increase in the resolution of MASH without worsening of fibrosis (RR 1.83, CI [1.47, 2.28], *p* < 0.00001), (RR 2.11, CI [1.63, 2.72], *p* < 0.00001), and the pooled studies were homogeneous (I^2^ = 0%), as shown in Figure 6 and Supplementary Table S2.

**Figure 7:**
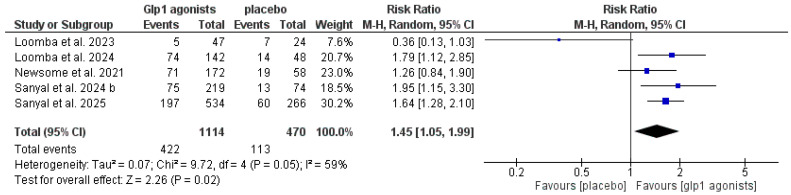
Forest plot of improvement of at least one stage of liver fibrosis without worsening of MASH in the overall population [14,29,30,32,33].

**Figure 8:**
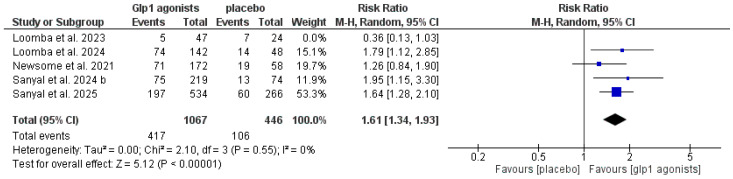
Forest plot of improvement of at least one stage of liver fibrosis without worsening of MASH in the overall population after excluding Loomba et al. 2023 [14,29,30,32,33].

The data showed that GLP-1 agonists were associated with a statistically significant increase in the improvement of at least one stage of liver fibrosis without the worsening of MASH compared to the control group (RR 1.61 [1.34, 1.93], *p* < 0.00001), and the pooled studies were homogeneous (*p* = 0.55, I^2^ = 0%) after excluding Loomba et al. 2023 [29], as shown in Table 3 and Figure 7 and Figure 8.

**Figure 9:**
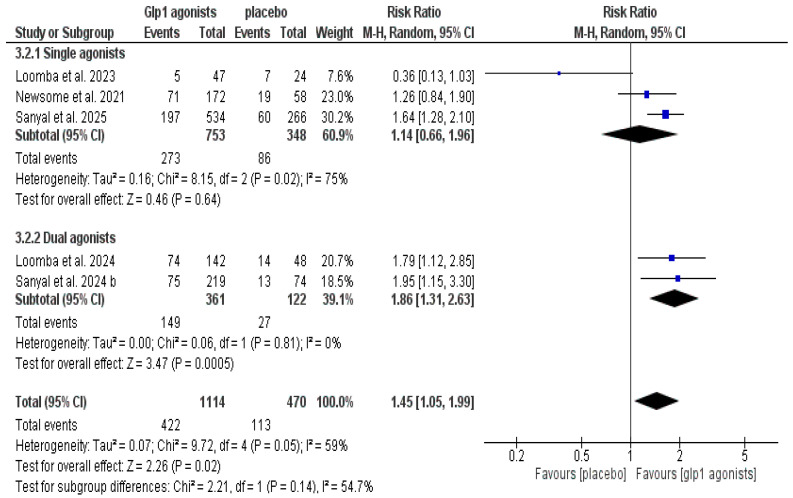
Forest plot of improvement of at least one stage of liver fibrosis without worsening of MASH in single or dual agonists [14,29,30,32,33].

**Figure 10:**
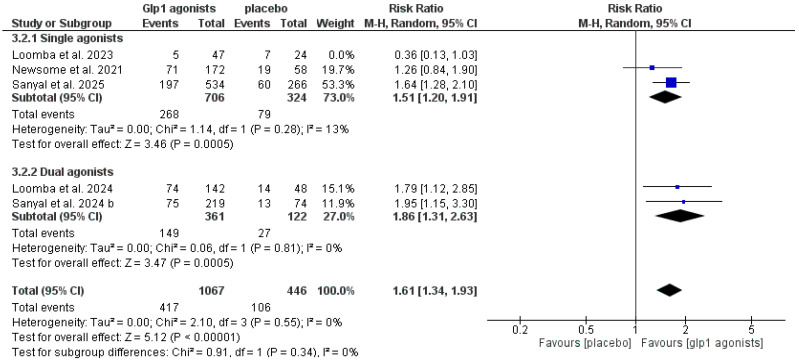
Forest plot of improvement of at least one stage of liver fibrosis without worsening of MASH in single or dual agonists after excluding Loomba et al. 2023 [14,29,30,32,33].

Similarly, the same findings were found for dual or triple agonists (RR 1.86, CI [1.31, 2.63], *p* = 0.0005) and single agonists (RR 1.51 [1.20, 1.91], *p* = 0.0005). The pooled studies in the dual agonist subgroup were homogeneous, I^2^ = 0, while in the single GLP-1 agonist group, they were homogeneous after excluding Loomba et al. 2023 [29] (I^2^ = 13), as shown in Supplementary Table S1 and Figure 9 and Figure 10.

However, only patients with diabetes had a statistically significant increase in improvement of at least one stage of liver fibrosis without worsening of MASH (RR 1.36, CI [1.05, 1.78], *p* = 0.02), (RR 1.75, CI [1.26, 2.44], *p* = 0.0009), and the pooled studies were homogeneous (I^2^ = 0%); there was no significant difference in patients without type 2 diabetes (*p* = 0.24), as shown in Supplementary Table S2 and Supplementary Figure S1.

On performing subgroup analyses based on the duration of treatment, we found that in treatment durations (48 and 72 weeks), GLP-1 agonists were associated with a significant improvement in the resolution of MASH (*p* = 0.04 in 48 weeks; *p* < 0.00001 in 72 weeks duration). The analysis was homogeneous in the 72 weeks and 48 weeks after excluding Loomba et al. 2023 [29], as shown in Supplementary Figures. GLP-1 agonists were associated with a significant increase in the improvement in fibrosis at 72 weeks treatment duration (*p* = 0.0005) and the pooled analysis was homogeneous (I^2^ = 13%), while there was no significant difference between the two groups at 48 weeks (*p* = 0.90); however, the pooled analysis was heterogeneous (I^2^ = 88%) (Supplementary Figures S2–S4).The data showed that GLP-1 agonists were associated with a statistically significant increase in the resolution of MASH and improvement in fibrosis compared to the control group (RR 2.01 [1.55, 2.62], *p* < 0.0001), as shown in Table 3 and Supplementary Figure S5. GLP-1 agonists were associated with a statistically significant reduction in the proportion of patients with liver fat reduction in more than 30% and 70% (RR 2.95, CI [1.88, 4.63], *p* < 0.00001), (RR 10.18, CI [2.32, 44.68], *p* = 0.002). There was no significant heterogeneity (I^2^ = 33%) in liver fat reduction in more than 30% after exclusion of Harrison et al. 2025 [13], while the pooled studies were heterogeneous in the liver fat reduction in more than 70% outcome (I^2^ = 62%), as shown in Table 3 and Supplementary Figures S6–S9.

The data showed that GLP-1 agonists were associated with a statistically significant reduction in liver fat percentage compared to the control group (SMD −0.72, CI [−0.99, −0.45], *p* < 0.0001); however, the pooled studies were heterogeneous (I^2^ = 78%), as shown in Table 3 and Supplementary Figure S10. Similarly, the same findings were found for both single GLP-1 agonists (SMD −0.67, CI [−1.08, −0.26], *p* = 0.001) and dual or triple agonists (SMD −0.81, CI [−1.07, −0.56], *p* < 0.00001), and the analysis was heterogeneous (I^2^ = 83% in single agonists), as shown in Supplementary Table S1 and Supplementary Figure S11. However, only patients with type 2 diabetes had a statistically significant reduction in liver fat (SMD −0.55, CI [−0.77, −0.33], *p* < 0.00001), and the pooled studies were homogeneous (I^2^ = 0%), as shown in Supplementary Table S2 and Supplementary Figures S12 and S13. On performing subgroup analysis based on the control group, GLP-1 agonists were associated with a statistically significant reduction in liver fat compared to the placebo (SMD −1.00, CI [−1.42, −0.57], *p* < 0.00001), and the pooled analysis was heterogeneous (I^2^ = 88%) or insulin only (SMD −0.58, CI [−0.87, −0.30], *p* < 0.0001); the pooled analysis was homogeneous (I^2^ = 0%). On the other hand, there was no significant difference between GLP-1 agonists and usual care or lifestyle interventions (*p* = 0.14), as shown in Supplementary Table S3 and Supplementary Figure S14. On performing meta-regression, we found no significant association between weight loss and the effect of GLP-1 agonists on liver fat content reduction (*p* = 0.881). Supplementary Figure S15.

### 3.6. Weight and HbA1c

The data showed that in the overall population, GLP-1 agonists were associated with a statistically significant weight reduction (SMD −1.11, CI [−1.57, −0.66], *p* < 0.0001) and HbA1c (SMD −0.81, CI [−1.16, −0.45], *p* < 0.00001) compared to the control group. However, the pooled analysis was heterogeneous (I^2^ = 95%, I^2^ = 90%), respectively, as shown in Table 3 and Supplementary Figures S16 and S17. Patients with type 2 diabetes had a statistically significant weight reduction (SMD −0.90, CI [−1.29, −0.50], *p* < 0.00001), while there was no statistically significant difference between patients without type 2 diabetes receiving GLP-1 agonists and the control group (SMD −0.97 [−2.18, 0.25], *p* = 0.12), as shown in Supplementary Table S2 and Supplementary Figure S18.

On performing subgroup analysis based on the control group, GLP-1 agonists were associated with a statistically significant decrease in weight (SMD −1.49 [−2.13, −0.85], *p* < 0.0001) and HbA1c (SMD −1.14, CI [−1.59, −0.68], *p* < 0.00001) compared to the placebo; however, the pooled studies were heterogeneous (*p* < 0.00001). Similarly, there was a significant reduction in weight compared to insulin (SMD −0.80, CI [−1.13, −0.46], *p* < 0.00001), while there was no significant difference between both groups in HBA1c (SMD −0.28, CI [−0.56, −0.00], *p* = 0.05). On the other hand, there was no significant difference between GLP-1 agonists and usual care or lifestyle interventions in weight (SMD −0.23 [−0.81, 0.35], *p* = 0.43) or HbA1c (SMD −0.16, CI [−0.56, 0.24], *p* = 0.44); the pooled results were heterogeneous in HbA1c (I^2^ = 70%), as shown in Supplementary Table S3 and Supplementary Figures S19–S21.

On performing subgroup analysis based on the intervention group, there was a significant difference in weight when single GLP-1 agonists (SMD −0.63, CI [−0.90, −0.37], *p* < 0.00001) or dual agonists (SMD −2.96, CI [−4.90, −1.02], *p* = 0.003) were administered. Similarly, HbA1c was significantly reduced when single GLP-1 agonists (SMD −0.86, CI [−1.31, −0.41], *p* = 0.0002) or dual agonists (SMD −1.13, CI [−1.36, −0.91], *p* < 0.00001) were administered. The pooled studies were heterogeneous, which was not resolved through the leave-one-out test except in HBA1c in the dual agonist subgroup after excluding Harrison et al. 2025 [13] (I^2^ = 0%), as shown in Supplementary Table S1 and Supplementary Figures S22–S24.

### 3.7. Biomarkers and Liver Enzymes

The data showed that there was a statistically significant difference between GLP-1 agonists and the control group (SMD −0.67, CI [−1.03, −0.31], *p* = 0.0003) in CRP levels, and the pooled studies were homogeneous after excluding Harrison et al. 2025 [13] (*p* = 0.17, I^2^ = 44%), as shown in Table 3 and Supplementary Figures S25 and S26.

The data showed that in the overall population, there was a significant difference between GLP-1 agonists and the control group in the levels of AST (SMD −0.48, CI [−0.83, −0.13], *p* = 0.008). However, the pooled analysis was heterogeneous (*p* < 0.00001, I^2^ = 91%), and the heterogeneity was not resolved through the leave-one-out test, as shown in Table 3 and Supplementary Figure S27.

On performing subgroup analysis based on the control group, GLP-1 agonists were associated with a statistically significant decrease in AST levels compared to the placebo (SMD −0.76, CI [−1.24, −0.27], *p* = 0.002); however, the pooled studies were heterogeneous (*p* < 0.00001, I^2^ = 94%), which was not resolved through the leave-one-out sensitivity analysis. On the other hand, there was no significant difference between GLP-1 agonists and usual care or lifestyle interventions (SMD −0.02, CI [−0.41, 0.38], *p* = 0.94), or when the control group received insulin (SMD −0.26, CI [−0.63, 0.12], *p* = 0.18), and the pooled analysis was homogeneous (I^2^ = 35%, I^2^ = 41%, respectively), as shown in Supplementary Table S3 and Supplementary Figure S28.

On performing subgroup analysis based on the intervention group, there was no significant difference between single GLP-1 agonists and the control (SMD −0.42, CI [−0.87, 0.03], *p* = 0.07), and the pooled analysis was heterogeneous (I^2^ = 92%). Dual and triple GLP-1 agonists were associated with a statistically significant reduction in AST levels compared to the control group (SMD −0.67, CI [−1.02, −0.32], *p* = 0.0002), and the pooled analysis was homogeneous (I^2^ = 35) after excluding Loomba et al. 2024 [30], as shown in Supplementary Table S2 and Supplementary Figures S29 and S30. Similarly, patients with type 2 diabetes receiving GLP-1 agonists had a statistically significant reduction in AST levels compared to the control group (SMD −0.24, CI [−0.46, −0.02], *p* = 0.03), and the pooled analysis was homogeneous (I^2^ = 26%), as shown in Supplementary Table S1 and Supplementary Figure S31.

The data showed that in the overall population, there was a significant difference between GLP-1 agonists and the control group in the levels of ALT (SMD −0.54, CI [−0.85, −0.23], *p* = 0.008). However, the pooled analysis was heterogeneous (*p* < 0.00001, I^2^ = 89%), and the heterogeneity was not resolved through the leave-one-out test, as shown in Table 3 and Supplementary Figure S32. On performing subgroup analysis based on the control group, GLP-1 agonists were associated with a statistically significant decrease in ALT levels compared to the placebo (SMD −0.72, CI [−1.15, −0.29], *p* = 0.001) or insulin (SMD = −0.52, CI [−0.79, −0.26], *p* = 0.0001). The pooled studies in insulin comparison were homogeneous (I^2^ = 0%); however, the pooled studies in placebo comparison were heterogeneous (*p* < 0.00001, I^2^ = 93%). On the other hand, there was no significant difference between GLP-1 agonists and usual care or lifestyle interventions (SMD = −0.08, CI [−0.42, 0.26], *p* = 0.66), and the pooled analysis was homogeneous (I^2^ = 12), as shown in Supplementary Table S3 and Supplementary Figure S33.

On performing subgroup analysis based on the intervention group, both dual and triple GLP-1 agonists and single agonists were associated with a statistically significant reduction in ALT levels compared to the control group (SMD −0.37, CI [−0.52, −0.23], *p* < 0.00001), (SMD −0.59, CI [−0.92, −0.26], *p* = 0.0004); the pooled analysis was homogeneous after excluding Loomba et al. 2024 [30] (I^2^ = 47%) and Newsome et al. 2021 [32] (I^2^ = 13%), respectively, as shown in Supplementary Table S1 and Supplementary Figures S34 and S35. Similarly, patients with type 2 diabetes receiving GLP-1 agonists had a statistically significant reduction in ALT levels compared to the control group (SMD −0.34 [−0.56, −0.12], *p* = 0.002), and the pooled analysis was homogeneous (I^2^ = 0), as shown in Supplementary Table S2 and Supplementary Figure S36.

### 3.8. Quality of Life and Adverse Events

The data showed that GLP-1 agonists significantly improved the physical domain of SF-36 compared to the control group (SMD 0.35, CI [0.12, 0.58], *p* = 0.003), while there was no statistically significant difference between both groups in the mental domain (SMD = 0.14, CI [−0.09, 0.38], *p* = 0.23), and the analysis was homogeneous (I^2^ = 0), as shown in Table 3 and Supplementary Figures S37 and S38.

The data showed that GLP-1 agonists were associated with a statistically significant increase in the incidence of adverse events (RR 1.10, CI [1.05, 1.14], *p* < 0.00001), gastrointestinal adverse events (RR 1.51, CI [1.37, 1.67], *p* < 0.00001), diarrhea (RR 2.02, CI [1.67, 2.43], *p* < 0.00001), nausea (RR 2.98, CI [2.49, 3.58], *p* < 0.00001), vomiting (RR 4.76, CI [3.40, 6.66], *p* < 0.00001), and fatigue (RR 1.52, CI [1.10, 2.10], *p* = 0.01) compared to the control group. The pooled results were homogeneous (I^2^ = 0) for gastrointestinal adverse events, diarrhea, and vomiting; I^2^ = 22% for adverse events; and I^2^ = 13% for nausea, as shown in Table 3 and Supplementary Figures S39–S44.

There was no significant difference between GLP-1 agonists and the control group in the incidence of serious adverse events (RR 1.13, CI [0.88, 1.44], *p* = 0.35), hypoglycemia events (RR 1.08, CI [0.72, 1.61], *p* = 0.71), injection site reactions (RR 1.01, CI [0.67, 1.51], *p* = 0.98), and gall bladder events (RR 1.75, CI [0.87, 3.54], *p* = 0.12), and the pooled analysis was homogenous (I^2^ = 0), as shown in Table 3. Similarly, there was no significant difference in the incidence of dizziness (RR 0.99 [0.67, 1.46], *p* = 0.97). The pooled studies were homogeneous after excluding Sanyal et al. 2024 [15] (*p* = 0.43, I^2^ = 0), as shown in Table 3 and Supplementary Figures S45–S50. Regarding the incidence of pancreatitis, among our included studies, six studies [16,20,25,26,30,32] reported zero cases. On the other hand, Sanyal et al. 2024 [14] reported that patients receiving survodutide had an asymptomatic elevation of pancreatic enzymes, while Sanyal et al. 2025 [33] reported a similar incidence in both groups, with 0.4% in the semaglutide group compared to 0.5% in the placebo group.

### 3.9. Publication Bias

As shown in Supplementary Figures S51–S60, the funnel plots of diarrhea, nausea, vomiting, HbA1c, ALT, and AST showed asymmetry, indicating publication bias, while adverse events, weight, and serious adverse events showed no significant asymmetry.

## 4. Discussion

We investigated the effect of GLP-1 agonists on patients with MASLD or MASH. Our systematic review and meta-analysis included 20 studies with 3216 participants. We found that GLP-1 agonists improved liver steatosis, steatohepatitis, and liver fibrosis. Moreover, it improved weight loss, HbA1c, and liver enzymes. However, they were associated with a significant increase in gastrointestinal adverse events, although there was no significant difference in serious adverse events. Both single and dual agonists had significant benefits; however, the magnitude was greater for dual agonists. On the other hand, there was no significant difference between GLP-1 agonists and the control group in weight loss, liver fat, or improvement in fibrosis among patients without type 2 diabetes. Furthermore, there was no significant difference between GLP-1 agonists and lifestyle interventions in HbA1c, weight, liver fat, or liver enzymes, whereas, compared to insulin, there was no significant difference between the groups in HbA1c and AST.

Liver steatosis could progress to steatohepatitis, fibrosis, cirrhosis, and liver cancer, and patients often remain asymptomatic until late stages [38]. Thus, patients with MASLD are at an increased risk of overall mortality as well as mortality due to liver cancer or liver diseases [39]. Indeed, liver fibrosis is associated with mortality in patients with MASLD [39]. Thus, resolution of steatohepatitis and fibrosis regression is important. However, liver steatosis and liver fibrosis have distinct pathological mechanisms [40]; liver steatosis arises from dysregulation in lipid metabolism and accumulation in the hepatocyte. This could further be complicated by inflammation, cell apoptosis, the activation of macrophages and hepatic stellate cells, and fibrosis. Thus, while reversal of these processess is supoposed to reverse fibrosis, some studies found only a significant improvement in steatosis only without significant improvement in fibrosis [12,32,41]. However, we found a significant improvement in both MASH resolution and liver fibrosis, similar to the findings of Sanyal et al. [33]. The included studies involved patients with F1 to F3 stages; thus, our findings could be applied to patients with mild to advanced fibrosis. Yet, further studies should investigate the effect of GLP-1 agonists in each fibrosis stage. While liver steatosis could be assessed through both invasive and noninvasive procedures, among our included studies, seven of them used biopsy, while the remaining thirteen studies used MRI for liver fat evaluation. Meanwhile, the improvement in fibrosis outcome was assessed through biopsy rather than noninvasive markers, which strengthens the reliability of our findings.

Overall, we found that GLP-1 agonists had a hepatoprotective effect. They significantly reduced liver fat content and improved the incidence of the resolution of steatohepatitis compared to the control. Consistently, liver enzymes, which are indicators of liver injury and inflammation, were significantly reduced. This is relevant since Loomba et al. found that reduction in liver enzymes after 6 months of follow-up was associated with improvements in the histological outcomes of patients with MASH after 18 months [42]. Also, we found a significant reduction in CRP levels, which denotes reduced inflammation. Similarly to our findings, Fang et al. found that GLP-1 agonists significantly reduced CRP and liver enzymes [12]. Moreover, GLP-1 agonists had a significant effect on the improvement of fibrosis, which is consistent with the findings of Mantovini et al. [11]. Both single and dual agonists were beneficial in improving liver fibrosis; however, dual agonists showed a higher effect, with an RR of 1.86, while single agonists had an RR of 1.51. Similarly, dual agonists had a higher effect on the resolution of MASH, with an RR of 6.25, while single agonists had an RR of 1.5. Consistent with that, we found a significant reduction in liver enzymes in both single and dual agonists, while dual agonists had a greater reduction compared to the control group. Consistent with our findings, Li et al. found that dual agonists significantly improved the resolution of liver steatosis and liver fibrosis and reduced liver enzyme levels [43]. On the other hand, we found no significant difference in patients without type 2 diabetes in liver fat content or improvement in liver fibrosis. We found only a significant improvement in the resolution of steatohepatitis, although liver enzymes were not significantly reduced. However, this could be due to the limited number of studies included in the analysis. Thus, further research is needed.

Weight loss and glycemic control are essential for reducing steatosis, inflammation, and fibrosis in patients with MASLD. Koutoukidis et al. found that every 1 kg of weight loss was associated with a 0.77 reduction in liver steatosis [44]. Gomez et al. found that weight reduction of more than 10% could reverse the incidence of fibrosis [6]. Achieving glycemic control is important to reduce the risk of fibrosis. Alexopoulos et al. showed that each 1% increase in HbA1c is associated with a 15% increase in the odds of fibrosis [45].

In our study, we found that GLP-1 agonists significantly reduced weight and HbA1c compared to the control. However, we found no significant association between GLP-a agonists’ effect on liver fat reduction and weight loss, which is consistent with the findings of Mantovani et al. [11]. Notably, we found that dual agonists had an SMD of −2.96 compared to −0.63 in single agonists, which denotes the superior effect of dual GLP-1 agonists. This could be attributed to the synergistic effect on multiple receptors, thus improving the overall efficacy of the drug [46]. Frias et al. showed that dual agonist tirzepatide had a superior effect in weight loss and HbA1c reduction compared to single agonist semaglutide [9]. Moreover, similarly to our findings, Li et al. investigated the effect of dual agonists and found that dual agonists had a higher effect on weight reduction with an MD of −11.38, while single agonists had an MD of −6.13 compared to the control group. However, their analysis was based on only six studies, and only two of them reported using dual agonists [43].

On the other hand, we found no significant difference between GLP-1 agonists and lifestyle interventions or standard of care on weight, HbA1c, or liver fat content. However, this could be explained by several factors, as the follow-up duration was short, which aligns with the findings of Alejandre et al., who found significant benefits on weight reduction in follow-ups up to six months, whereas partial weight regain occurred on long-term follow-up [7]. Furthermore, the unblinding of participants carries the risk of co-interventions. Also, we found no significant difference in weight in patients without type 2 diabetes. This is inconsistent with the literature. The FDA approved GLP-1 agonists for weight reduction in patients without T2DM. Moreover, Vosoughi et al. found that patients without T2DM receiving GLP-1 agonists had significantly greater mean weight loss than those with T2DM [47,48]. Thus, further research is needed to investigate the effect of GLP-1 agonists in patients with MASLD without type 2 diabetes, since the analysis of this subgroup involved a limited number of studies.

We found a significantly higher risk of adverse events. However, there was no significant difference in the incidence of serious side effects or hypoglycemic events, which signifies their safety. The increase in gastrointestinal side effects, including diarrhea, nausea, and vomiting, is consistent with the literature and could be explained by their mechanism of action, which alters GIT mobility. However, these could be managed through patient education, proper dose escalation protocols, and symptomatic treatment [49]. Also, there was no significant difference in the incidence of injection site reactions compared to the placebo, denoting local tolerability.

## 5. Strengths and Limitations

Our study has several strengths. Our study is comprehensive and includes a large sample of patients with hepatic steatosis and steatohepatitis. Additionally, the diverse setting of the included studies supports the generalizability of our findings. We included patients where the diagnosis of MASLD was confirmed through MRI or biopsy. This is particularly important since most of the conducted studies included patients with mild steatosis, where a diagnosis based on ultrasound would not be accurate [50]. It is dependent on operator assessment and could yield false negative results when steatosis is less than 20% [51]. Furthermore, the accuracy of ultrasound decreases in overweight and obese individuals [52]. In contrast, MRI is the gold standard for the noninvasive assessment of liver steatosis. Furthermore, we excluded studies that included a relatively low proportion of patients with MASLD. Additionally, we investigated several outcomes, including the quality of life and various adverse events. To our knowledge, these outcomes were not investigated in the previous meta-analyses. Moreover, we conducted several subgroup analyses. For instance, since some of the included studies included more than one control group, we conducted a separate analysis for the overall population and each subgroup to avoid the unit of analysis error, in contrast to the SR conducted by Mantovani et al. [11]. Additionally, we conducted subgroup analysis based on the class of the used GLP-1 agonist, which was not investigated in the previous meta-analyses. Additionally, we conducted a subgroup analysis to investigate the effect of GLP-1 agonists in patients with and without type 2 diabetes. However, the number of studies that provided data for this comparison was low. Thus, further studies should investigate the effect of diabetes status to further confirm our findings. Additionally, we conducted a sensitivity analysis in order to resolve the heterogeneity.

Our study has some limitations. First, we only included English studies. Second, the heterogeneity in the analysis could be attributed to several factors, including the different settings or baseline characteristics of the included studies. Thus, for outcomes with high heterogeneity, such as weight and HBA1c, the findings should be interpreted with caution and further studies with more standardized interventions and longer follow-ups are needed to better elucidate the sources of heterogeneity and improve the robustness of the pooled estimates. Moreover, the analysis of patients without type 2 diabetes involved a limited number of studies. Similarly, the subgroup based on dual or triple agonists was based on a limited number of recently conducted studies. Thus, further studies should evaluate the effect of GLP-1 agonists, and the findings should be interpreted with caution. Some of the included studies had some concerns about the risk of bias due to unblinding and the possibility of co-intervention. However, we conducted subgroup analysis based on the control to verify the effect of GLP-1 agonists compared to each control group. Moreover, we detected publication bias in the analysis. The included studies used different definitions as some of the studies followed the old terminology (NAFLD, NASH), while others followed the new terminology (MASLD, MASH). However, the studies that followed the old terminology included patients with metabolic risk factors such as diabetes. Thus, all the included studies involved patients with metabolic risk factors in addition to hepatic steatosis.

## 6. Clinical Implications

This systematic review and meta-analysis provided evidence on the efficacy and safety of GLP-1 agonists in patients with MASLD or MASH. Our analysis confirmed that GLP-1 receptor agonists (single or dual) had a significant benefit on the histological features of MASLD/MASH. The improvements in steatosis, steatohepatitis, and liver fibrosis suggest that GLP-1 agonists can be considered to address liver injury and fibrosis, and prevent further progression.

The observed improvements in weight, HbA1c, and liver enzymes demonstrate the efficacy of GLP-1 agonists in ameliorating the underlying metabolic dysfunction. However, these findings should be interpreted with caution, given the heterogeneity of the included studies. Furthermore, liver enzymes are biomarkers of liver injury that may be influenced by several factors, and their long-term relationship with clinical outcomes could not be fully assessed in the included studies. The finding that GLP-1 agonists did not significantly outperform lifestyle interventions in several metabolic and hepatic parameters suggests that GLP-1 agonists could be used for patients with difficulty adhering to lifestyle interventions and sustaining weight loss, rather than as a replacement, since they did not have superior effects to lifestyle interventions. The comparable efficacy to insulin in glycemic control and liver enzyme levels, combined with the additional benefits of weight loss and potential fibrosis improvement, suggests that in T2DM patients with MASLD or MASH, GLP-1 agonists could be a superior choice over insulin where clinically appropriate. However, the non-significant findings in patients without type 2 diabetes should be interpreted with caution given the limited number of studies and necessitate further investigation. The significant increase in gastrointestinal adverse events is a well-characterized effect and a key practical consideration. While serious adverse events were not increased, the high rate of nausea, vomiting, and diarrhea necessitates patient education and management.

## 7. Conclusions

Both single and dual GLP-1 agonists significantly improved weight loss, HbA1c, and liver enzymes in patients with MASLD/MASH. Moreover, they significantly increased the resolution of metabolic-associated steatohepatitis and improved liver fibrosis. However, they were associated with a significantly higher risk of gastrointestinal adverse events. GLP-1 agonists had comparable effects to insulin in HbA1c control. Compared to lifestyle interventions, they had comparable effects on weight reduction, HbA1c control, and liver enzymes. On the other hand, in patients without type 2 diabetes, the available data are limited and potentially underpowered to provide definitive conclusions regarding GLP-1 agonists’ effects on weight loss and hepatic outcomes. Thus, the lack of statistically significant results should be interpreted as a lack of sufficient evidence rather than as evidence of no effect, and further research is needed before clinical recommendations can be made for this subgroup.

## Figures and Tables

**Table 1:** Baseline characteristics.

Study ID	Groups	No of Participants	Age in Years Mean (SD)	Male (%)	BMI (kg/m^2^) Mean (SD)	Weight (KG) Mean (SD)	Type 2 Diabetes (%)	HbA1c % Mean (SD)	Hepatic Fat Content Mean (SD)	ALT (U/L) Mean (SD)	AST (U/L) Mean (SD)
Armstrong et al. 2016 [20]	Liraglutide	26	50 (11)	18 (69%)	34.2 (4.7)	101 (18)	9 (35%)	5.9 (0.7)	N/A	77 (34)	51 (22)
	Placebo	26	52 (12)	13 (50%)	37.7 (6.2)	108 (18)	8 (31%)	6.0 (0.9)	N/A	66 (42)	51 (27)
Bi et al. 2014 [21]	Exenatide	11	50.8 (13.3)	7 (63.6%)	25.1 (3.6)	71.1 (11.6)	11 (100%)	8.6 (1.3)	27.4 (18.2)	30.7 (17.6)	26.3 (9.3)
	Insulin	11	53.5 (8)	5 (45.5%)	24.5 (2)	65.2 (6.6)	11 (100%)	9.1 (1)	3.7 (16.6)	20.2 (6.6)	23.0 (7)
Dutour et al. 2016 [22]	Exenatide	22	51 (9.4)	13 (59%)	37.2 (8.4)	104 (23.5)	22 (100%)	N/A	N/A	48 (35)	33 (25)
	Reference treatment	22	52 (9.4)	8 (36%)	35 (5.6)	95 (14)	22 (100%)	N/A	N/A	48 (27)	30 (13)
Flint et al. 2021 [23]	Semaglutide	34	59.5 (10.1)	23 (67.6%)	N/A	105.1 (15.3)	7.3 (0.9%)	7.3 (0.9)	22.1 (15.6)	53.6 (47.8)	38.1 (27.2)
	Placebo	33	60.5 (8.5)	24 (72.7%)	N/A	102.3 (12.7)	7.4 (1%)	7.4 (1.0)	20.9 (14.2)	44.4 (34.7)	34.2 (21.5)
Guo et al. 2020 [24]	Liraglutide	31	53.1 (6.3)	16 (52%)	29.2 (4.2)	84.3 (10.8)	31 (100%)	7.5 (1.3)	26.4 (3.2)	33.2 (15.8)	29.6 (10.8)
	Insulin	30	52.0 (8.7)	18 (60%)	28.3 (3.8)	83.8 (11.2)	30 (100%)	7.4 (0.9)	25(4.3)	31.5 (12.6)	27.9 (12.1)
	Placebo	30	52.6 (3.9)	20 (67%)	28.6 (3.7)	82.2 (12.4)	30 (100%)	7.4 (1)	25.8 (4.5)	30.5 (13.4)	28.1 (12.6)
Harrison et al. 2025 [13]	Pemvidutide	70	49.2 (9.3)	34 (48.6%)	35.7 (4.8)	99.81 (17.7)	21 (30%)	6.8 (1.2)	21.2 (7.4)	35.6 (18.5)	25.8 (10)
	Placebo	24	47.9 (14)	10 (41.67%)	36.9 (4.7)	105.1 (20.8)	6 (5%)	6.2 (0.6)	23.8 (9.2)	39.5 (21.4)	23.8 (10.0)
Ji et al. 2025 [25]	Ecnoglutide	499	34.4 (7.6)	253 (50.7%)	32.5 (4.1)	91.4 (16.1)	0	5.3 (0.3)		28.5 (17)	20.6 (7.2)
	Placebo	165	33.8 (7.2)	82 (50%)	32.4 (4.1)	91.0 (16.3)	0	5.3 (0.4)		26 (3.3)	1 9 (1.5)
Khoo et al. 2019 [26]	Liraglutide	15	38.6 (8.2)	15 (100%)	34.3 (3.9)	102.7 (16.2)	0	N/A	31.4 (9.3)	87 (32)	45 (14)
	Lifestyle interventions	15	43.6 (9.9)	13 (87%)	32.2 (3.2)	89.6 (12.7)	0	N/A	30.8 (17.5)	88 (38)	52 (27)
Kuchay et al. 2020 [27]	Dulaglutide	32	46.6 (9.1)	23 (72%)	29.6 (3.6)	85.8 (13.3)	32 (100%)	8.4 (1)	17.9 (7.2)	70.1 (30.1)	49.9 (22.7)
	Usual care	32	48.1 (8.9)	22 (69%)	29.9 (3.9)	83.7 (13)	32 (100%)	8.4 (1)	17.1 (7.7)	68.1 (30.8)	46.1 (21.1)
Liu et al. 2020 [28]	Exentide	35	47.63 (10.14)	19 (54%)	28.49 (3.02)	79.28 (9.64)	35 (100%)	8.32 (0.94)	42.21 (16.83)	42.71 (23.19)	31.29 (17.32)
	Insulin	36	50.56 (11.78)	19 (53%)	27.84 (3.10)	77.63 (13.70)	36 (100%)	8.58 (0.91)	35.47 (13.78)	32.81 (22.37)	25.11 (14.09)
Loomba et al. 2023 [29]	Semaglutide	47	59.9 (7.1)	16 (34%)	34.6 (5.9)	95.2 (18.7)	35 (75%)	7.1 (1.3)	11.34 (5.04)	56.1 (39.4)	51.9 (24.2)
	Placebo	24	58.7 (9.7)	6 (25%)	35.5 (6.0)	98.6 (22.2)	18 (75%)	7.2 (1.2)	11.65 (5.23)	41.8 (23.5)	42.9 (20.3)
Loomba et al. 2024 [30]	Tirzepatide	142	54.7 (11.2)	60 (42.3%)	36.2 (6)	101.1 (21.4)	82 (57.7%)	6.5 (1.1)	18.5 (7.6)	62.6 (34.2)	50 (24.5)
	Placebo	48	53.5 (11.6)	21 (43.8%)	36 (6.7)	96 (21.6)	29 (60%)	6.8 (1.2)	18.2 (6.8)	59.7 (30.3)	52.3 (21.3)
Moolla et al. 2025 [31]	Liraglutide	15	48 (15.5)	8 (53.3%)	35.7 (6.6)	106.9 (21.7)	0	N/A	24.3 (8.9)	58 (31)	36 (11.6)
	Lifestyle interventions	14	48 (15)	7 (50%)	36.4 (5.6)	104.2 (21.7)	0	N/A	21.1 (9)	61 (29.9)	37 (15)
Newsome et al. 2021 [32]	Semaglutide	240	55.8 (10.4)	90 (37.5%)	35.7 (2.3)	97.4 (21)	149 (62.1%)	7.3 (1.2)	N/A	70.6 (59.5)	55.3 (43)
	Placebo	80	52.4 (10.8)	36 (45%)	36.2 (2.4)	101.3 (23.3)	50 (62%)	7.3 (1.2)	N/A	74.7 (68.8)	54.6 (45.3)
Sanyal et al. 2024 ^a^ [15]	Retatrutide	79	46.8 (12.3)	43 (87.8%)	38.4 (5.3)	110.1 (19.1)	N/A	5.5 (0.4)	20 (6.9)	33.2 (4)	24.2 (1.8)
	Placebo	19	45.5 (10.7)	9 (47.4%)	38.6 (4.6)	110.8 (16.5)	N/A	5.56 (0.33)	15.6 (5.8)	31.6 (2.1)	24.5 (1.2)
Sanyal et al. 2024 ^b^ [14]	Survodutide	219	50.1 (13.2)	108 (49.3%)	35.9 (6.4)	101.8 (22.9)	84 (38.4%)	6.9 (1)	19.5 (7.5)	57.9 (43.5)	45.9 (34.9)
	Placebo	74	53 (11.5)	30 (40.5%)	35.49 (6.44)	98.09 (20.78)	29 (39%)	7.08 (0.87)	19.62 (7.59)	57.3 (36.6)	51.3 (40.9)
Sanyal et al. 2025 [33]	Semaglutide	534	56.3 (11.4)	221 (41.3%)	34.3 (7.2)	95.4 (24.5)	296 (55.4%)	N/A	N/A	67.8 (42.3)	53.2 (28.6)
	Placebo	266	55.4 (12)	122 (45.8%)	35 (7.1)	97.6 (24.6)	296 (55.4%)	N/A	N/A	67.9 (44.7)	52.8 (33.1)
Shankar et al. 2024 [16]	Cotadutide	50	57.7 (11.2)	23 (46%)	37.2(6.2)	99.3(18.8)	29 (58%)	6.7 (1.2)	19.5 (7.5)	44.8(20.8)	35.1 (15.3)
	Placebo	24	52.2 (13.5)	10 (41.7%)	37.6 (5.1)	102.2 (18.1)	12 (50%)	6.8 (1.5)	19.1 (8.2)	48.8 (31.2)	38.4 (24.6)
Smits et al. 2016 [34]	Liraglutide	17	60.8 (7.4)	12 (70.6%)	32.8 (4.1)	103.2 (13.2)	17 (100%)	7.4 (0.8)	20.9 (14)	28.9 (12)	24.2 (7.8)
	Placebo	17	65.8 (5.8)	13 (76.5%)	30.6 (2.9)	95.8 (9.9)	17 (100%)	7.5 (0.8)	18.7 (11.1)	32 (21.4)	22.2 (7.4)
Yan et al. 2019 [35]	Liraglutide	24	43.1 (9.7)	17 (70.8%)	30.1 (3.3)	86.6 (12.9)	24 (100%)	7.8 (1.4)	15.4 (5.6)	43.2 (21.2)	31.1 (11.7)
	Insulin	24	45.6 (7.6)	14 (58.3%)	29.6 (3.5)	85.6 (14.2)	24 (100%)	7.7 (0.9)	14.9 (5.5)	39.5 (25.7)	33.2 (17.4)

ALT: Alanine transaminase. AST: Aspartate aminotransferase. BMI: Body mass index. HBA1c: Glycated hemoglobin. N/A: not available. Superscript letters (a, b) indicate multiple publications by the same first author within the same year.

**Table 2:** Summary of the included studies.

Study ID	Country	NCT	Population	T2DM	NASH	Sample	Intervention	Dose and Frequency	Control	MRI or Biopsy	Follow up Duration
Armstrog et al. 2016 [20]	United Kingdom	NCT01237119	Patients with biopsy-confirmed non-alcoholic steatohepatitis	Both patients with and without diabetes	Yes	52	Liraglutide	1.8 mg daily	Placebo	Biopsy	48 weeks
Bi 2014 [21]	China	NCT01147627	Drug-naive T2DM patients	Yes	No	33	Exentide	10 lg twice daily	Insulin, pioglitazone	MRI	24 weeks
Dutour et al. 2016 [22]	France	NR	Obese subjects with T2D and glycated hemoglobin (HbA1c) levels of 6.5–10%	Yes	No	44	Exenatide	10 µg twice daily	Reference treatment	MRI	26 weeks
Flint et al. 2021 [23]	Germany	NCT03357380	Subjects aged 18–75 years, with a BMI of 25–40 kg/m^2^ and liver stiffness of 2.50–4.63 kPa measured by MRE and >4.0 kPa	Both patients with and without diabetes	No	67	Semaglutide	0.4 mg/day	Placebo	MRI	48 weeks
Guo et al. 2020 [24]	China	hiCTR2000035091	Patients with type 2 diabetes and NAFL	Yes	No	96	Liraglutide	1.8 mg	Insulin, placebo	MRI	26 weeks
Harrison et al. 2025 [13]	United States	NCT05006885	Patients with a BMI >−28.0 kg/m^2^ and LFC >−10%	Both patients with and without diabetes	No	94	Pemvidutide	1.2, 1.8, and 2.4 mg	Placebo	MRI	12 weeks
Ji et al. 2025 [25]	China	NCT05813795	Adults with overweight or obesity	No	No	664	Ecnoglutide	1.2, 1.8, and 2.4 mg	Placebo	MRI	40 weeks
Khoo et al. 2019 [26]	Singapore	NR	Obese adults with non-alcoholic fatty liver disease	No	No	30	Liraglutide	3 mg	Lifestyle intervention	MRI	26 weeks
Kuchay et al. 2020 [27]	India	NCT03590626	Patients with type 2 diabetes and NAFLD	Yes	No	64	Dulaglutide	Dulaglutide 1.5 mg weekly	Usual care	MRI	24 weeks
Liu et al. 2020 [28]	China	NCT02303730	Type 2 diabetes mellitus (T2DM) and non-alcoholic fatty liver disease	Yes	No	71	Exenatide	Subcutaneous exenatide 10 μg twice daily for 20 weeks	Insulin	MRI	24 weeks
Loomba et al. 2023 [29]	Europe and the USA	NCT03987451	Patients with NASH and compensated cirrhosis.	Both patients with and without diabetes	Yes	71	Semaglutide	Once-weekly subcutaneous 2.4 mg	Placebo	Both	48 weeks
Loomba et al. 2024 [30]	Multicenter (10 countries)	NCT04166773	Participants with biopsy-confirmed MASH and stage F2 or F3 (moderate or severe) fibrosis	Both patients with and without diabetes	Yes	190	Tirzepatide	Doses of 5 mg, 10 mg, or 15 mg once-weekly	Placebo	Biopsy	52 weeks
Moolla et al. 2025 [31]	United Kingdom	EudraCT (2016-002045-36)	Participants with MASLD, without type 2 diabetes	No	Included a subpopulation	29	Liraglutide	Liraglutide treatment 1.8 mg/day	Lifestyle interventions	MRI	12 weeks
Newsome et al. 2021 [32]	Multicenter (16 countries)	NCT02970942	Patients with biopsy- confirmed NASH and liver fibrosis of stage F1, F2, or F3.	Both patients with and without diabetes	Yes	320	Semaglutide	Once-daily subcutaneous semaglutide at a dose of 0.1, 0.2, or 0.4 mg	Placebo	Biopsy	72 weeks
Sanyal et al. 2024 ^a^ [15]	United States	NCT04881760	Participants with MASLD	No	NR	98	Retatrutide	Retatrutide 1 mg, 4 mg, 8 mg, or 12 mg administered once-weekly	Placebo	MRI	24 weeks
Sanyal et al. 2024 ^b^ [14]	Multicenter (25 countries)	NCT04771273	Adults with biopsy-confirmed MASH and fibrosis	Both patients with and without diabetes	Yes	293	Survodutide	Once-weekly subcutaneous injections of survodutide at a dose of 2.4, 4.8, or 6.0 mg	Placebo	Both	48 weeks
Sanyal et al. 2025 [33]	Multicenter (253 clinical sites in 37 countries)	NCT04822181	Patients with biopsy-defined MASH and fibrosis	Both patients with and without diabetes	Yes	800	Semaglutide	Once-weekly subcutaneous semaglutide at a dose of 2.4 mg	Placebo	Biopsy	72 weeks
Shankar et al. 2024 [16]	23 sites across the United States and Puerto Rico	NCT04019561	Participants with biopsy- proven noncirrhotic metabolic dysfunction-associated steatohepatitis (MASH) with fibrosis.	Both patients with and without diabetes	Yes	74	Cotadutide	Subcutaneous once-daily cotadutide 300 mg, cotadutide 600 mg20:K20	Placebo	Both	19 weeks
Smits et al. 2016 [34]	Netherlands	NCT01744236	Overweight patients with type 2 diabetes	Yes	No	51	Liraglutide	Liraglutide 1.8 mg once daily	Placebo, sitagliptin	MRI	12 weeks
Yan et al. 2019 [35]	China	NCT02147925	Patients with type 2 diabetes mellitus and non-alcoholic fatty liver disease	Yes	No	75	Liraglutide	Subcutaneous 1.8 mg once daily	Sitagliptin and insulin	MRI	26-week

MASLD: Metabolic-associated liver dysfunction. MRE: Magnetic resonance elastography. MRI: Magnetic resonance imaging. MASH: Metabolic dysfunction-associated steatohepatitis. NASH: Non-alcoholic steatohepatitis. NAFLD: Non-alcoholic fatty liver disease. LFC: Liver fat content. T2DM: Type 2 diabetes mellitus. NR: Not reported. F1: Mild fibrosis. F2: Moderate fibrosis. F3: Severe fibrosis. NCT: National clinical trial. Superscript letters (a, b) indicate multiple publications by the same first author within the same year.

**Table 3:** Effect estimates (SMD/RR) and heterogeneity (I^2^) for key outcomes in the main meta-analysis and leave-one-out sensitivity analyses.

Main Analysis							Sensitivity Analysis (Leave-One-Out Test)					
Outcome	Comparison	No. of studies	Effect Estimate		Heterogeneity		Exclusion of	No. of studies	Effect estimate		Heterogeneity	
			SMD, 95%CI/RR, 95%CI	*p* value	*p* value	I^2^			SMD, 95%CI/RR, 95%CI	*p* value	*p* value	I^2^
Resolution of MASH without worsening fibrosis	Overall population (GLP-1 agonist vs. control)	6	3.03 [1.80, 5.11]	Test for overall effect: (*p* < 0.0001)	(*p* = 0.003)	I^2^ = 72%	Not resolved	N/A				
Improvement of at least one stage of liver fibrosis without worsening of MASH	Overall population (GLP-1 agonist vs. placebo)	5	1.45 [1.05, 1.99]	(*p* = 0.02)	(*p* = 0.05)	I^2^ = 59%	Loomba et al. 2023 [29]	4	1.61 [1.34, 1.93]	(*p* < 0.00001)	*p* = 0.55	I^2^ = 0%
Resolution of MASH and improvement in fibrosis	Overall population (Semaglutide vs. placebo)	3	2.01 [1.55, 2.62]	(*p* < 0.00001)	(*p* = 0.18);	I^2^ = 41%	Not performed since there is no significant heterogeneity	N/A				
Weight	Overall population (GLP-1 agonist vs. control)	18	−1.11 [−1.57, −0.66]	(*p* < 0.00001)	(*p* < 0.00001)	I^2^ = 95%	Not resolved	N/A				
HBA1c	Overall population (GLP-1 agonist vs. control)	15	−0.81 [−1.16, −0.45]	(*p* < 0.00001)	(*p* < 0.00001)	I^2^ = 90%	Not resolved	N/A				
Liver fat	Overall population (GLP-1 agonist vs. control)	17	−0.72 [−0.99, −0.45]	(*p* < 0.00001)	(*p* < 0.00001)	I^2^ = 78%	Not resolved	N/A				
AST	Overall population (GLP-1 agonist vs. control)	16	−0.48 [−0.83, −0.13]	(*p* = 0.008)	(*p* < 0.00001);	I^2^ = 91%	Not resolved	N/A				
ALT	Overall population (GLP-1 agonist vs. control)	17	−0.54 [−0.85, −0.23]	(*p* = 0.0008)	(*p* < 0.00001)	I^2^ = 89%	Not resolved	N/A				
CRP	Overall population (GLP-1 agonist vs. control)	4	−0.54 [−0.90, −0.18]	(*p* = 0.004)	(*p* = 0.05)	I^2^ = 62%	Harrison et al. 2025 [13]	3	−0.67 [−1.03, −0.31]	(*p* = 0.0003)	(*p* = 0.17)	I^2^ = 44%
Quality of life SF-36 (physical component)	Overall population GLP-1 agonists vs. placebo)	2	0.35 [0.12, 0.58]	(*p* = 0.003)	(*p* = 0.99)	I^2^ = 0%	Not performed since there is no significant heterogeneity	N/A				
Quality of life SF-36 (mental component)	Overall population GLP-1 agonists vs. placebo)	2	0.14 [−0.09, 0.38]	(*p* = 0.23)	(*p* = 0.73)	I^2^ = 0%	Not performed since there is no significant heterogeneity	N/A				
Liver fat reduction ≥30%	Overall population (GLP-1 agonist vs. control)	6	3.32 [1.89, 5.83]	(*p* < 0.0001)	(*p* = 0.05)	I^2^ = 54%	Harrison et al. 2025 [13]	5	2.95 [1.88, 4.63]	(*p* < 0.00001)	(*p* = 0.20)	I^2^ = 33%
							Flint et al. 2021 [23]	5	3.85 [2.05, 7.23]	(*p* < 0.0001)	(*p* = 0.15)	I^2^ = 40%
Liver fat reduction ≥70%	Overall population (GLP-1 agonist vs. control)	2	10.18 [2.32, 44.68]	(*p* = 0.002)	(*p* = 0.11)	I^2^ = 62%	Not performed since there is no significant heterogeneity	N/A				
Adverse events	Overall population (GLP-1 agonist vs. control)	11	1.10 [1.05, 1.14]	(*p* < 0.00001)	(*p* = 0.23)	I^2^ = 22%	Not performed since there is no significant heterogeneity	N/A				
Serious adverse events	Overall population (GLP-1 agonist vs. control)	12	1.13 [0.88, 1.44]	(*p* = 0.35)	(*p* = 0.98)	I^2^ = 0%	Not performed since there is no significant heterogeneity	N/A				
Hypoglycemia event	Overall population (GLP-1 agonist vs. control)	5	1.08 [0.72, 1.61]	(*p* = 0.71)	(*p* = 0.45)	I^2^ = 0%	Not performed since there is no significant heterogeneity	N/A				
Gastrointestinal side effects	Overall population (GLP-1 agonist vs. control)	5	1.51 [1.37, 1.67]	(*p* < 0.00001)	(*p* = 0.44)	I^2^ = 0%	Not performed since there is no significant heterogeneity	N/A				
Diarrhea	Overall population (GLP-1 agonist vs. control)	13	2.02 [1.67, 2.43]	(*p* < 0.00001)	(*p* = 0.70)	I^2^ = 0%	Not performed since there is no significant heterogeneity	N/A				
Nausea	Overall population (GLP-1 agonist vs. control)	13	2.98 [2.49, 3.58]	(*p* < 0.00001)	(*p* = 0.32)	I^2^ = 13%	Not performed since there is no significant heterogeneity	N/A				
Vomiting	Overall population (GLP-1 agonist vs. control)	12	4.76 [3.40, 6.66]	(*p* < 0.00001)	(*p* = 0.63)	I^2^ = 0%	Not performed since there is no significant heterogeneity	N/A				
Fatigue	Overall population (GLP-1 agonist vs. control)	7	1.52 [1.10, 2.10]	(*p* = 0.01)	(*p* = 0.80)	I^2^ = 0%	Not performed since there is no significant heterogeneity	N/A				
Dizziness	Overall population (GLP-1 agonist vs. control)	6	1.00 [0.47, 2.12]	(*p* = 0.99)	(*p* = 0.06)	I^2^ = 52%	Sanyal 2024 a [15]	5	0.99 [0.67, 1.46]	(*p* = 0.97)	(*p* = 0.43)	I^2^ = 0%
Injection site reaction	Overall population (GLP-1 agonist vs. control)	4	1.01 [0.67, 1.51]	(*p* = 0.98)	(*p* = 0.76)	I^2^ = 0%	Not performed since there is no significant heterogeneity	N/A				
Gallbladder events	Overall population (GLP-1 agonist vs. control)	4	1.75 [0.87, 3.54]	(*p* = 0.12)	(*p* = 0.90)	I^2^ = 0%	Not performed since there is no significant	N/A				

MASH: Metabolic dysfunction-associated steatohepatitis. CRP: C-reactive protein. ALT: Alanine transaminase. AST: Aspartate aminotransferase. SMD: Standarized mean difference. RR: Risk ratio.

## Data Availability

No new data were created or analyzed in this study.

## Supplementary Materials

The following supporting information can be downloaded at https://www.mdpi.com/article/10.3390/pharmaceutics18010086/s1, Reference [53] is cited in the Supplementary Material.
