# Supplementary material for: The Effect of GLP-1 Agonists on Patients with Metabolic-Associated Steatotic Liver Disease: A Systematic Review and Meta-Analysis
Source: Pharmaceutics. 2026 Jan 9;18(1):86. doi: 10.3390/pharmaceutics18010086 (PMC12844962; doi:10.3390/pharmaceutics18010086)
Supplement: Supplementary file 1 [file pharmaceutics-18-00086-s001.zip › pharmaceutics-4065940-supplementary.pdf]

Search strategy:-

Search date: 14/10/2025

PubMed:

("Glucagon-Like Peptide-1 Receptor Agonists"[Mesh] OR "Glucagon Like Peptide 1 Receptor Agonists" OR "GLP-1 Agonists" OR "GLP 1 Agonists" OR "GLP-1 Receptor Agonists" OR "GLP 1 Receptor Agonists" OR "GLP1 Agonists" OR "GLP-1R Agonists" OR "GLP 1R Agonists" OR "GLP1R Agonists" OR "incretin Mimetics" OR "GLP-1 Analogs" OR "GLP 1 Analogs" OR exenatide OR liraglutide OR benaglutide OR beinaglutide OR lixisenatide OR dulaglutide OR loxenatide OR efinopegdutide OR semaglutide OR tirzepatide OR albiglutide) AND ("Non-alcoholic Fatty Liver Disease"[Mesh] OR "Non alcoholic Fatty Liver Disease" OR "Nonalcoholic Fatty Liver" OR "Nonalcoholic Fatty Livers" OR NAFLD OR "Nonalcoholic Fatty Liver Disease" OR "Nonalcoholic Steatohepatitis" OR "Nonalcoholic Steatohepatitides" OR "Metabolic associated fatty liver disease" OR MAFLD OR "Metabolic-dysfunction associated fatty liver disease" OR "Metabolic-dysfunction associated steatotic liver disease" OR MASLD OR "Fatty Liver" OR "Nonalcoholic Fatty Liver" OR NASH OR "Metabolic dysfunction-associated Steatohepatitis" OR MASH OR "liver fibrosis" OR "hepatic steatosis")

Results: 1,097

Search fields: All fields

WOS:

("Glucagon Like Peptide 1 Receptor Agonists" OR "GLP-1 Agonists" OR "GLP 1 Agonists" OR "GLP-1 Receptor Agonists" OR "GLP 1 Receptor Agonists" OR "GLP1 Agonists" OR "GLP-1R Agonists" OR "GLP 1R Agonists" OR "GLP1R Agonists" OR "incretin Mimetics" OR "GLP-1 Analogs" OR "GLP 1 Analogs" OR exenatide OR liraglutide OR benaglutide OR beinaglutide OR lixisenatide OR dulaglutide OR loxenatide OR efinopegdutide OR semaglutide OR tirzepatide OR albiglutide) AND ("Non alcoholic Fatty Liver Disease" OR "Nonalcoholic Fatty Liver" OR "Nonalcoholic Fatty Livers" OR NAFLD OR "Nonalcoholic Fatty Liver Disease" OR "Nonalcoholic Steatohepatitis" OR "Nonalcoholic Steatohepatitides" OR "Metabolic associated fatty liver disease" OR MAFLD OR "Metabolic-dysfunction associated fatty liver disease" OR "Metabolic-dysfunction associated steatotic liver disease" OR MASLD OR "Fatty Liver" OR "Nonalcoholic Fatty Liver" OR NASH OR "Metabolic dysfunction-associated Steatohepatitis" OR MASH OR "liver fibrosis" OR "hepatic steatosis")

Results: 1,372
Search fields: Topic

Scopus :

("Glucagon Like Peptide 1 Receptor Agonists" OR "GLP-1 Agonists" OR "GLP 1 Agonists" OR "GLP-1 Receptor Agonists" OR "GLP 1 Receptor Agonists" OR "GLP1 Agonists" OR "GLP-1R Agonists" OR "GLP 1R Agonists" OR "GLP1R Agonists" OR "incretin Mimetics" OR "GLP-1 Analogs" OR "GLP 1 Analogs" OR exenatide OR liraglutide OR benaglutide OR beinaglutide OR lixisenatide OR dulaglutide OR loxenatide OR efinopegdutide OR semaglutide OR tirzepatide OR albiglutide) AND ("Non alcoholic Fatty Liver Disease" OR "Nonalcoholic Fatty Liver" OR "Nonalcoholic Fatty Livers" OR NAFLD OR "Nonalcoholic Fatty Liver Disease" OR "Nonalcoholic Steatohepatitis" OR "Nonalcoholic Steatohepatitides" OR "Metabolic associated fatty liver disease" OR MAFLD OR "Metabolic-dysfunction associated fatty liver disease" OR "Metabolic-dysfunction associated steatotic liver disease" OR MASLD OR "Fatty Liver" OR "Nonalcoholic Fatty Liver" OR NASH OR "Metabolic dysfunction-associated Steatohepatitis" OR MASH OR "liver fibrosis" OR "hepatic steatosis")

Results: 2,721
Search fields: : TITLE-ABS-KEY

Supplementary Table S1. Subgroup analysis based on the drug class

Main analysis							Sensitivity analysis (leave-one-out test)					
outcome	subgroup based on intervention	no. Of studies	Effect estimate		Heterogeneity		Exclusion of	no. Of studies	Effect estimate		Heterogeneity	
			SMD, 95%CI /RR, 95%CI	(P value)	P value	I2			SMD, 95% CI /RR, 95% CI	P value	P value	I2
resolution of MASH without worsening fibrosis	Overall population (GLP 1 agonist vs control)	6	3.03 [1.80, 5.11]	(P < 0.0001)	(P = 0.003)	I^2 = 72 %	not resolved	_				
	single agonists	4	1.91 [1.61, 2.25]	(P < 0.00001)	(P = 0.43)	I^2 = 0%	not performed since theres no significant heterogeneity	_				
	Dual or triple agonists	2	6.25 [3.31, 11.79]	(P < 0.00001)	(P = 0.51)	I^2 = 0%	not performed since theres no significant heterogeneity	_				
improvement of at least one stage of liver fbrosis without worsening of MASH	Overall population (GLP 1 agonist vs placebo)	5	1.45 [1.05, 1.99]	(P = 0.02)	(P = 0.05)	I^2 = 59 %	Loomba et al. 2023	4	1.61 [1.34, 1.93]	(P < 0.00001)	P = 0.55	I^2 = 0%
	single agonists	3	1.14 [0.66, 1.96]	(P = 0.64)	(P = 0.02)	I^2 = 75 %	Loomba et al. 2023	2	1.51 [1.20, 1.91]	(P = 0.0005)	(P = 0.28)	I^2 = 13%
	Dual or triple agonists	2	1.86 [1.31, 2.63]	(P = 0.0005)	(P = 0.81)	I^2 = 0%	not performed since theres no significant heterogeneity	_				
Weight	Overall population (GLP 1	18	-1.11 [-	(P < 0.00001)	(P < 0.00001)	I^2 = 95 %	not resolved	_				

	agonist vs control)		1.57, -0.66]									
	single agonists	14	-0.63 [-0.90, -0.37]	(P < 0.00001)	(P < 0.00001)	I^2 = 79 %	not resolved	_				
	Dual or triple agonists	4	-2.96 [-4.90, -1.02]	(P = 0.003)	(P < 0.00001)	I^2 = 98 %	not resolved	_				
HBA1c	Overall population (GLP 1 agonist vs control)	15	-0.81 [-1.16, -0.45]	(P < 0.00001)	(P < 0.00001)	I^2 = 90 %	not resolved	_				
	single agonists	12	-0.86 [-1.31, -0.41]	(P = 0.0002)	(P < 0.00001)	I^2 = 91 %	not resolved	_				
	Dual or triple agonists	3	-0.76 [-1.42, -0.10]	(P = 0.02)	(P < 0.0001)	I^2 = 90 %	Harrison et al. 2025	2	-1.13 [-1.36, -0.91]	(P < 0.00001)	(P = 0.44)	I^2 = 0%
Liver fat	Overall population (GLP 1 agonist vs control)	17	-0.72 [-0.99, -0.45]	(P < 0.00001)	(P < 0.00001)	I^2 = 78 %	not resolved	_				
	single agonists	12	-0.67 [-1.08, -0.26]	(P = 0.001)	(P < 0.00001)	I^2 = 83 %	not resolved	_				
	Dual or triple agonists	5	-0.81 [-1.07, -0.56]	(P < 0.00001)	(P = 0.10)	I^2 = 49 %	not performed since theres no significant heterogeneity	_				
AST	Overall population (GLP 1 agonist vs control)	16	-0.48 [-0.83, -0.13]	(P = 0.008)	(P < 0.00001)	I^2 = 91 %	not resolved	_				

	single agonists	13	-0.42 [-0.87, 0.03]	(P = 0.07)	(P < 0.00001)	I^2 = 92 %	not resolved	_				
	Dual or triple agonists	3	-0.82 [-1.16, -0.47]	(P < 0.00001)	(P = 0.08)	I^2 = 61 %	:oomba et al. 2024	3	-0.67 [-1.02, -0.32]	(P = 0.0002)	(P = 0.22)	I^2 = 35%
							Sanyal 2024 b	3	-0.95 [-1.26, -0.63]	(P < 0.00001)	(P = 0.17)	I^2 = 47%
ALT	Overall population (GLP 1 agonist vs control)	17	-0.54 [-0.85, -0.23]	(P = 0.0008)	(P < 0.00001)	I^2 = 89 %	not resolved	_				
	single agonists	13	-0.46 [-0.87, -0.05]	(P = 0.03)	(P < 0.00001)	I^2 = 91 %	Newsome eta l. 2021	12	-0.37 [-0.52, -0.23]	(P < 0.00001)	(P = 0.32)	I^2 = 13%
	Dual or triple agonists	4	-0.76 [-1.17, -0.35]	(P = 0.0003)	(P = 0.005)	I^2 = 77 %	Loomba et al. 2024	3	-0.59 [-0.92, -0.26]	(P = 0.0004)	(P = 0.15)	I^2 = 47%

MASH: metabolic dysfunction-associated steatohepatitis. GLP 1:Glucagon-like peptide-1 . ALT :alanine transaminase. AST: aspartate aminotransferase. SMD : Standarized mean difference. RR: Risk ratio

Supplementary Table S2. Subgroup analysis based on diabetes status

Main analysis					Sensitivity analysis (leave-one-out test)			
outcome	subgroup based on	no. Of	Effect estimate	Heterogeneity	Exclusion of	no. Of	Effect estimate	Heterogeneity

	interventon	studies	SMD, 95%CI /RR, 95%CI	(P value)	P value	I2		studies	SMD, 95% CI /RR, 95% CI	P value	P value	I2
resolution of MASH without worsening fibrosis	Overall population (GLP 1 agonist vs control)	6	3.03 [1.80, 5.11]	(P < 0.0001)	(P = 0.003)	I^2 = 72 %	not resolved	_				
	patients with T2DM	3	1.83 [1.47, 2.28]	(P < 0.00001)	(P = 0.38)	I^2 = 0%	not performed since there is no significant heterogeneity	_				
	patients without T2DM	3	2.11 [1.63, 2.72]	(P < 0.00001)	(P = 0.80)	I^2 = 0%	not performed since there is no significant heterogeneity	_				
Improvement of at least one stage of liver fbrosis	Overall population (GLP 1 agonist vs control)	5	1.45 [1.05, 1.99]	(P = 0.02)	(P = 0.05)	I^2 = 59 %	Loomba et al. 2023	4	1.61 [1.34, 1.93]	(P < 0.00001)	P = 0.55	I^2 = 0%
	patients with T2DM	2	1.36 [1.04, 1.78]	(P = 0.02)	(P = 0.79)	I^2 = 0%	not performed since there is no significant heterogeneity	_				
	patients without T2DM	2	1.54 [0.75, 3.15]	(P = 0.24)	(P = 0.03)	I^2 = 78 %	not performed	_				
Weight	Overall population (GLP 1 agonist vs control)	18	-1.11 [-1.57, -0.66]	(P < 0.00001)	(P < 0.00001)	I^2 = 95 %	not resolved	_				

	patients with T2DM	8	-0.90 [-1.29, -0.50]	(P < 0.00001)	(P = 0.0002)	I^2 = 76 %	not resolved	_				
	patients without T2DM	4	-0.97 [-2.18, 0.25]	(P = 0.12)	(P < 0.00001)	I^2 = 92 %	not resolved	_				
Liver fat	Overall population (GLP 1 agonist vs control)	17	-0.72 [-0.99, -0.45]	(P < 0.00001)	(P < 0.00001)	I^2 = 78 %	not resolved	_				
	patients with T2DM	7	-0.55 [-0.77, -0.33]	(P < 0.00001)	(P = 0.73)	I^2 = 0%	not performed since there is no significant heterogeneity	_				
	patients without T2DM	4	-0.86 [-2.24, 0.52]	(P = 0.22)	(P < 0.00001);	I^2 = 94 %	Ji et al. 2025	3	-0.18 [-0.66, 0.29]	(P = 0.45)	(P = 0.19);	I^2 = 39 %
AST	Overall population (GLP 1 agonist vs control)	16	-0.48 [-0.83, -0.13]	(P = 0.008)	(P < 0.00001);	I^2 = 91 %	not resolved	_				
	patients with T2DM	7	-0.24 [-0.46, -0.02]	(P = 0.03)	(P = 0.23)	I^2 = 26 %	not performed since there is no significant heterogeneity	_				
	patients without T2DM	2	-0.29 [-0.70, 0.13]	(P = 0.17)	(P = 0.35)	I^2 = 0%	not performed since there is no significant heterogeneity	_				
ALT	Overall population (GLP 1 agonist vs control)	17	-0.54 [-0.85, -0.23]	(P = 0.0008)	Test for overall effect: Z =	I^2 = 89 %	not resolved	_				

					1.36 (P = 0.17)			
	patients with T2DM	7	-0.34 [-0.56, -0.12]	(P = 0.002)	(P = 0.44)	I² = 0%	not performed since there is no significant heterogeneity	_
	patients without T2DM	2	-0.20 [-0.62, 0.21]	(P = 0.33)	(P = 0.41)	I² = 0%	not performed since there is no significant heterogeneity	_

MASH: metabolic dysfunction-associated steatohepatitis. GLP 1:Glucagon-like peptide-1 . ALT :alanine transaminase. AST: aspartate aminotransferase. SMD : Standarized mean difference. RR: Risk ratio

Supplementary Table S3. Sungroup analysis based on the control group

Main analysis							Sensitivity analysis (leave-one-out test)					
outcome	subgroup based on control	no. Of studies	Effect estimate		Heterogeneity		Exclusion of	no. Of studies	Effect estimate		Heterogeneity	
			SMD, 95%CI /RR, 95%CI	(P value)	P value	I2			SMD, 95%CI /RR, 95%CI	P value	P value	I2
Weight	Overall population (GLP 1 agonist vs control)	18	-1.11 [-1.57, -0.66]	(P < 0.00001)	(P < 0.00001)	I² = 95%	not resolved	_				
	GLP1 agonists vs Control group (placebo)	11	-1.49 [-2.13, -0.85]	(P < 0.00001)	(P < 0.00001)	I² = 97%	not resolved	_				

	GLP1 agonists vs Control group (insulin)	4	-0.80 [-1.13, -0.46]	(P < 0.00001)	(P = 0.27);	I² = 23%	not performed since there is no significant heterogeneity	_				
	GLP1 agonists vs Control group (usual care or lifestyle intervention)	4	-0.46 [-1.06, 0.14]	(P = 0.13)	(P = 0.02)	I² = 70%	Dutour et al. 2016	3	-0.23 [-0.81, 0.35]	(P = 0.43)	(P = 0.10);	I² = 56%
HBA1c	Overall population (GLP 1 agonist vs control)	15	-0.81 [-1.16, -0.45]	(P < 0.00001)	(P < 0.00001)	I² = 90%	not resolved	_				
	GLP1 agonists vs Control group (placebo)	10	-1.14 [-1.59, -0.68]	(P < 0.00001)	(P < 0.00001)	I² = 92%	not resolved	_				
	GLP1 agonists vs Control group (insulin)	4	-0.28 [-0.56, -0.00]	(P = 0.05)	(P = 0.86)	I² = 0%	not performed since there is no significant heterogeneity	_				
	GLP1 agonists vs Control group (usual care or lifestyle intervention)	2	-0.16 [-0.56, 0.24]	(P = 0.44)	(P = 0.47)	I² = 0%	not performed since there is no significant heterogeneity	_				
Liver fat	Overall population (GLP 1 agonist vs control)	17	-0.72 [-0.99, -0.45]	(P < 0.00001)	(P < 0.00001)	I² = 78%	not resolved	_				
	GLP1 agonists vs Control group (placebo)	10	-1.00 [-1.42, -0.57]	(P < 0.00001)	(P < 0.00001)	I² = 88%	not resolved	_				

	GLP1 agonists vs Control group (insulin)	4	-0.58 [-0.87, -0.30]	(P < 0.0001)	(P = 0.93)	I² = 0%	not performed since there is no significant heterogeneity	_
	GLP1 agonists vs Control group (usual care or lifestyle intervention)	4	-0.34 [-0.80, 0.11]	(P = 0.14)	(P = 0.12)	I² = 49%	not performed since there is no significant heterogeneity	_
AST	Overall population (GLP 1 agonist vs control)	16	-0.48 [-0.83, -0.13]	(P = 0.008)	(P < 0.00001);	I² = 91%	not resolved	_
	GLP1 agonists vs Control group (placebo)	9	-0.76 [-1.24, -0.27]	(P = 0.002)	(P < 0.00001)	I² = 94%	not resolved	_
	GLP1 agonists vs Control group (insulin)	4	-0.26 [-0.63, 0.12]	(P = 0.18)	(P = 0.16)	I² = 41%	not performed since there is no significant heterogeneity	_
	GLP1 agonists vs Control group (usual care or lifestyle intervention)	4	-0.02 [-0.41, 0.38]	(P = 0.94)	(P = 0.20)	I² = 35%	not performed since there is no significant heterogeneity	_
ALT	Overall population (GLP 1 agonist vs control)	17	-0.54 [-0.85, -0.23]	(P = 0.0008)	(P < 0.00001)	I² = 89%	not resolved	_
	GLP1 agonists vs Control group (placebo)	10	-0.72 [-1.15, -0.29]	(P = 0.001)	(P < 0.00001)	I² = 93%	not resolved	_

	GLP1 agonists vs Control group (insulin)	4	-0.52 [-0.79, -0.26]	(P = 0.0001)	(P = 0.75)	I² = 0%	not performed since there is no significant heterogeneity	_
	GLP1 agonists vs Control group (usual care or lifestyle intervention)	4	-0.08 [-0.42, 0.26]	(P = 0.66)	(P = 0.33)	I² = 12%	not performed since there is no significant heterogeneity	_

MASH: metabolic dysfunction-associated steatohepatitis. GLP 1:Glucagon-like peptide-1 . ALT :alanine transaminase. AST: aspartate aminotransferase. SMD : Standarized mean difference. RR: Risk ratio

----

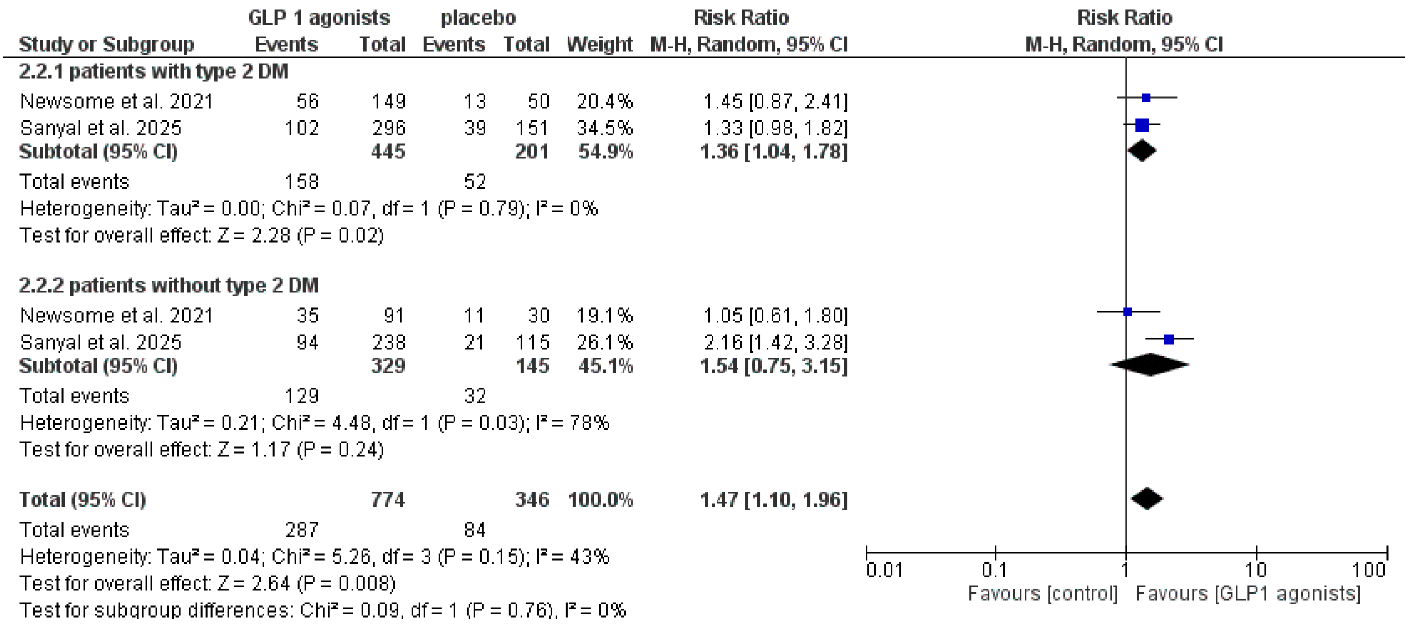

Supplementary Figure S1. Forest plot of improvement of at least one stage of liver fibrosis without worsening of MASH in patients with and without type 2 diabetes.

Study or Subgroup	Glp1 agonists Events	Glp1 agonists Total	placebo Events	placebo Total	Weight	Risk Ratio M-H, Random, 95% CI
**5.1.1 48 weeks**						
Armstrong et al. 2016	9	26	2	26	8.9%	4.50 [1.07, 18.85]
Loomba et al. 2023	16	47	5	24	15.3%	1.63 [0.68, 3.92]
Sanyal et al. 2024 b	94	219	4	74	14.0%	7.94 [3.02, 20.85]
**Subtotal (95% CI)**		**292**		**124**	**38.2%**	**3.77 [1.23, 11.55]**
Total events	119		11			
Heterogeneity: Tau² = 0.67; Chi² = 6.61, df = 2 (P = 0.04); I² = 70%						
Test for overall effect: Z = 2.33 (P = 0.02)						
**5.1.2 52 weeks**						
Loomba et al. 2024	77	142	5	48	15.8%	5.21 [2.24, 12.10]
**Subtotal (95% CI)**		**142**		**48**	**15.8%**	**5.21 [2.24, 12.10]**
Total events	77		5			
Heterogeneity: Not applicable						
Test for overall effect: Z = 3.83 (P = 0.0001)						
**5.1.3 72 weeks**						
Newsome et al. 2021	77	172	10	58	20.0%	2.60 [1.44, 4.67]
Sanyal et al. 2025	336	534	91	266	26.0%	1.84 [1.54, 2.20]
**Subtotal (95% CI)**		**706**		**324**	**46.0%**	**1.95 [1.51, 2.51]**
Total events	413		101			
Heterogeneity: Tau² = 0.01; Chi² = 1.24, df = 1 (P = 0.27); I² = 19%						
Test for overall effect: Z = 5.14 (P < 0.00001)						
**Total (95% CI)**		**1140**		**496**	**100.0%**	**3.03 [1.80, 5.11]**
Total events	609		117			
Heterogeneity: Tau² = 0.27; Chi² = 17.89, df = 5 (P = 0.003); I² = 72%						
Test for overall effect: Z = 4.16 (P < 0.0001)						
Test for subgroup differences: Chi² = 5.78, df = 2 (P = 0.06), I² = 65.4%						

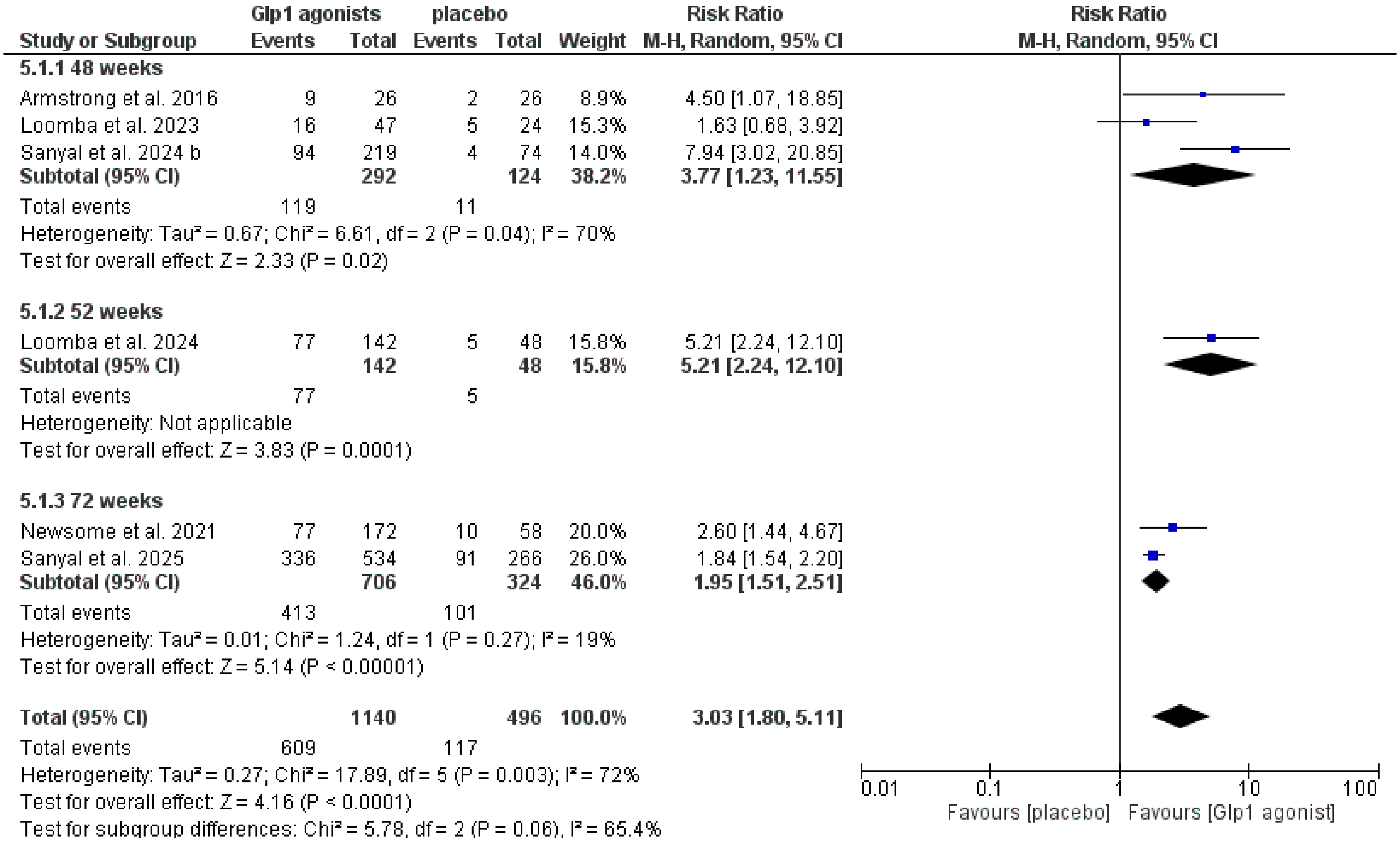

Supplementary Figure S2. Forest plot showing subgroup analysis based on treatment duration in the resolution of AMSH without worsening of fibrosis outcome.

Study or Subgroup	Glp1 agonists Events	Glp1 agonists Total	placebo Events	placebo Total	Weight	Risk Ratio M-H, Random, 95% CI
**5.1.1 48 weeks**						
Armstrong et al. 2016	9	26	2	26	11.5%	4.50 [1.07, 18.85]
Loomba et al. 2023	16	47	5	24	0.0%	1.63 [0.68, 3.92]
Sanyal et al. 2024 b	94	219	4	74	17.2%	7.94 [3.02, 20.85]
**Subtotal (95% CI)**		**245**		**100**	**28.7%**	**6.65 [2.99, 14.81]**
Total events	103		6			
Heterogeneity: Tau² = 0.00; Chi² = 0.44, df = 1 (P = 0.51); I² = 0%						
Test for overall effect: Z = 4.64 (P < 0.00001)						
**5.1.2 52 weeks**						
Loomba et al. 2024	77	142	5	48	19.1%	5.21 [2.24, 12.10]
**Subtotal (95% CI)**		**142**		**48**	**19.1%**	**5.21 [2.24, 12.10]**
Total events	77		5			
Heterogeneity: Not applicable						
Test for overall effect: Z = 3.83 (P = 0.0001)						
**5.1.3 72 weeks**						
Newsome et al. 2021	77	172	10	58	23.3%	2.60 [1.44, 4.67]
Sanyal et al. 2025	336	534	91	266	28.8%	1.84 [1.54, 2.20]
**Subtotal (95% CI)**		**706**		**324**	**52.2%**	**1.95 [1.51, 2.51]**
Total events	413		101			
Heterogeneity: Tau² = 0.01; Chi² = 1.24, df = 1 (P = 0.27); I² = 19%						
Test for overall effect: Z = 5.14 (P < 0.00001)						
**Total (95% CI)**		**1093**		**472**	**100.0%**	**3.47 [1.86, 6.46]**
Total events	593		112			
Heterogeneity: Tau² = 0.34; Chi² = 17.71, df = 4 (P = 0.001); I² = 77%						
Test for overall effect: Z = 3.92 (P < 0.0001)						
Test for subgroup differences: Chi² = 12.01, df = 2 (P = 0.002), I² = 83.3%						

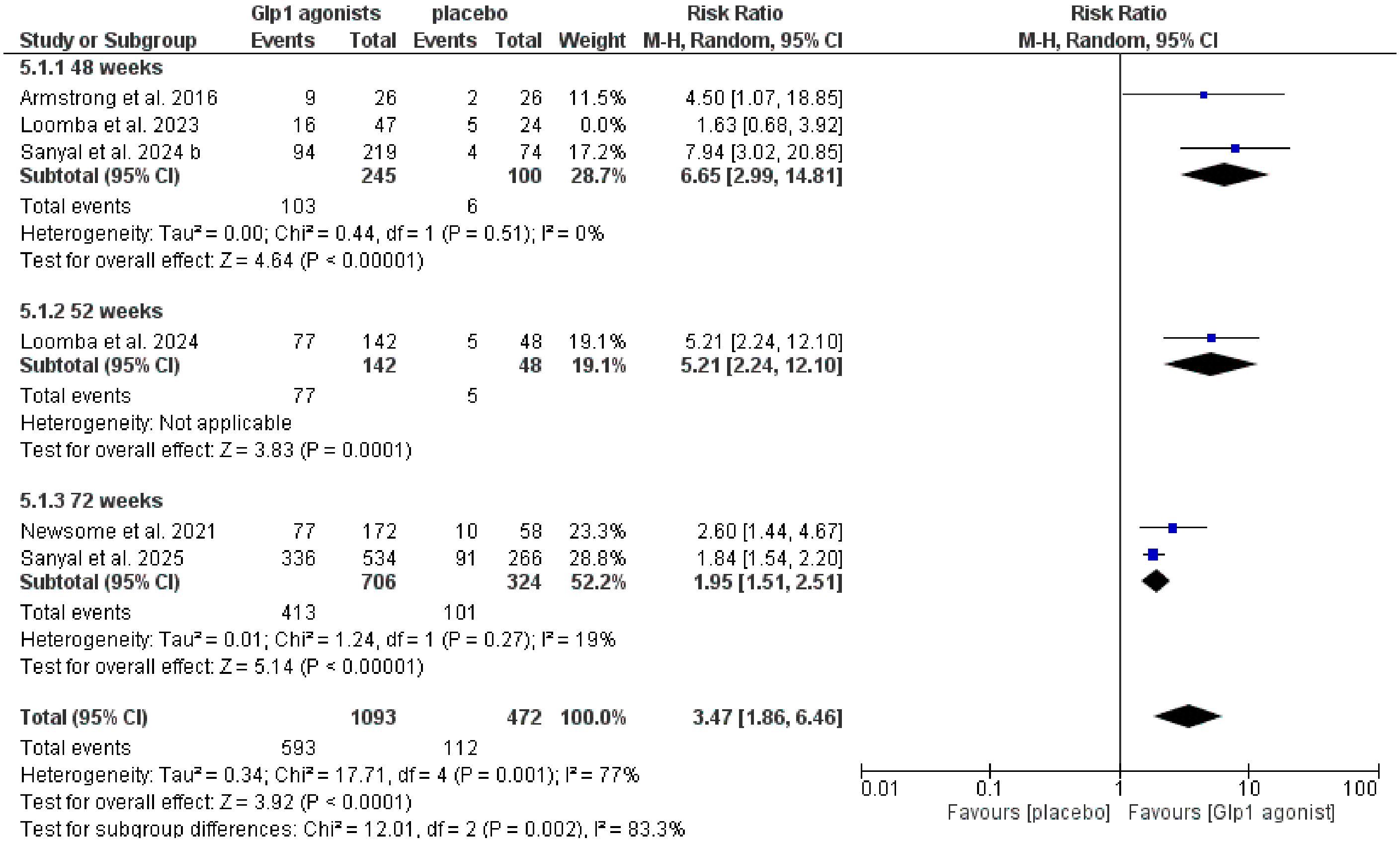

Supplementary Figure S3. Forest plot showing subgroup analysis based on treatment duration in the resolution of AMSH without worsening of fibrosis outcome after excluding Loomba et al. 2023.

Study or Subgroup	Glp1 agonists Events	Glp1 agonists Total	placebo Events	placebo Total	Weight	Risk Ratio M-H, Random, 95% CI
**5.2.1 48 weeks**						
Loomba et al. 2023	5	47	7	24	7.6%	0.36 [0.13, 1.03]
Sanyal et al. 2024 b	75	219	13	74	18.5%	1.95 [1.15, 3.30]
**Subtotal (95% CI)**		**266**		**98**	**26.1%**	**0.90 [0.17, 4.63]**
Total events	80		20			
Heterogeneity: Tau² = 1.23; Chi² = 8.01, df = 1 (P = 0.005); I² = 88%						
Test for overall effect: Z = 0.13 (P = 0.90)						
**5.2.2 52 weeks**						
Loomba et al. 2024	74	142	14	48	20.7%	1.79 [1.12, 2.85]
**Subtotal (95% CI)**		**142**		**48**	**20.7%**	**1.79 [1.12, 2.85]**
Total events	74		14			
Heterogeneity: Not applicable						
Test for overall effect: Z = 2.43 (P = 0.02)						
**5.2.3 72 weeks**						
Newsome et al. 2021	71	172	19	58	23.0%	1.26 [0.84, 1.90]
Sanyal et al. 2025	197	534	60	266	30.2%	1.64 [1.28, 2.10]
**Subtotal (95% CI)**		**706**		**324**	**53.3%**	**1.51 [1.20, 1.91]**
Total events	268		79			
Heterogeneity: Tau² = 0.00; Chi² = 1.14, df = 1 (P = 0.28); I² = 13%						
Test for overall effect: Z = 3.46 (P = 0.0005)						
**Total (95% CI)**		**1114**		**470**	**100.0%**	**1.45 [1.05, 1.99]**
Total events	422		113			
Heterogeneity: Tau² = 0.07; Chi² = 9.72, df = 4 (P = 0.05); I² = 59%						
Test for overall effect: Z = 2.26 (P = 0.02)						
Test for subgroup differences: Chi² = 0.82, df = 2 (P = 0.66), I² = 0%						

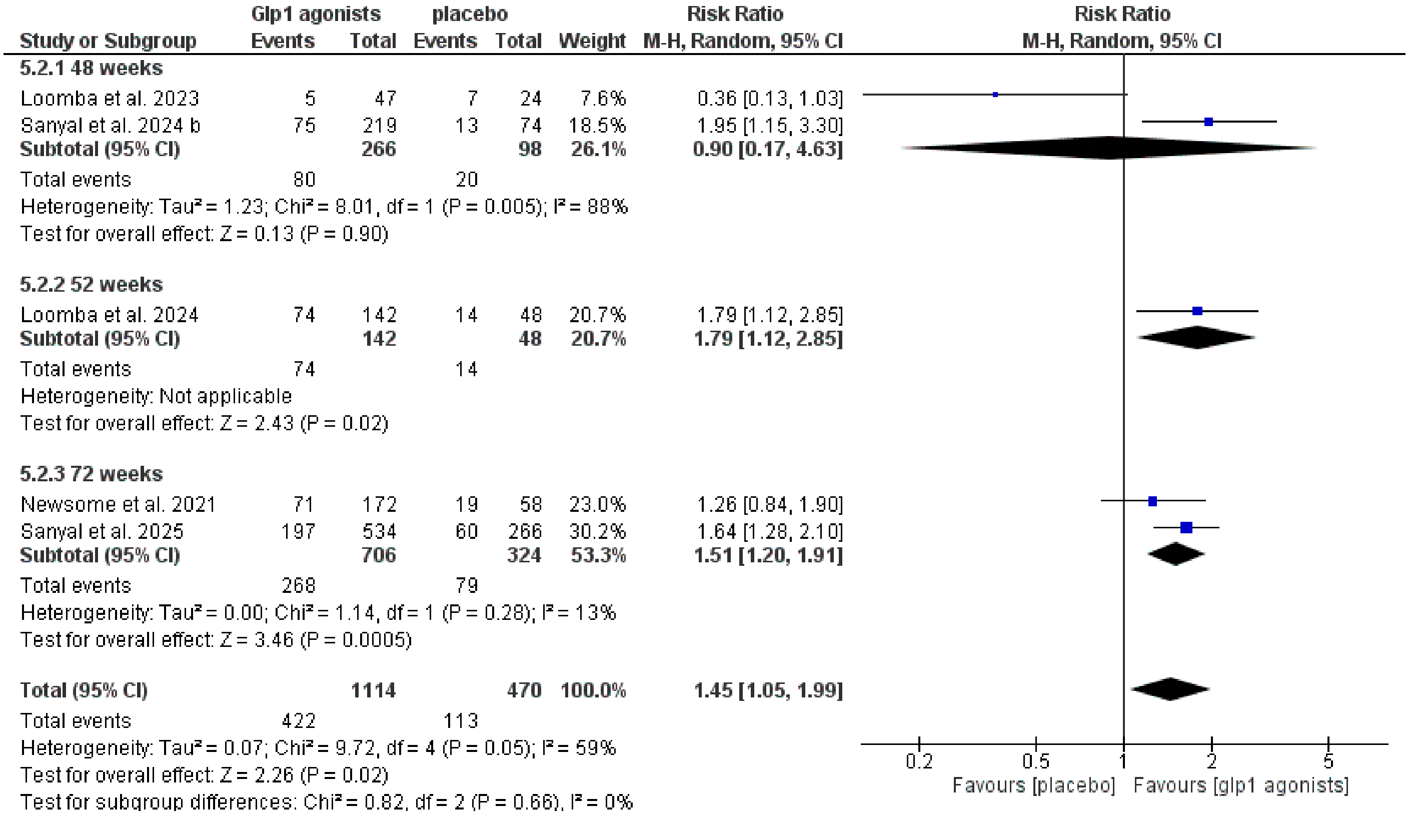

Supplementary Figure S4. Forest plot showing subgroup analysis based on treatment duration in improvement of at least one stage of liver fibrosis without worsening of MASH outcome .

Study or Subgroup	Glp1 agonists Events	Glp1 agonists Total	placebo Events	placebo Total	Weight	Risk Ratio M-H, Fixed, 95% CI
Loomba et al. 2023	3	47	3	24	5.3%	0.51 [0.11, 2.34]
Newsome et al. 2021	64	172	9	58	18.0%	2.40 [1.28, 4.51]
Sanyal et al. 2025	175	534	43	266	76.7%	2.03 [1.50, 2.73]
**Total (95% CI)**		**753**		**348**	**100.0%**	**2.01 [1.55, 2.62]**
Total events	242		55			
Heterogeneity: Chi² = 3.41, df = 2 (P = 0.18); I² = 41%						
Test for overall effect: Z = 5.18 (P < 0.00001)						

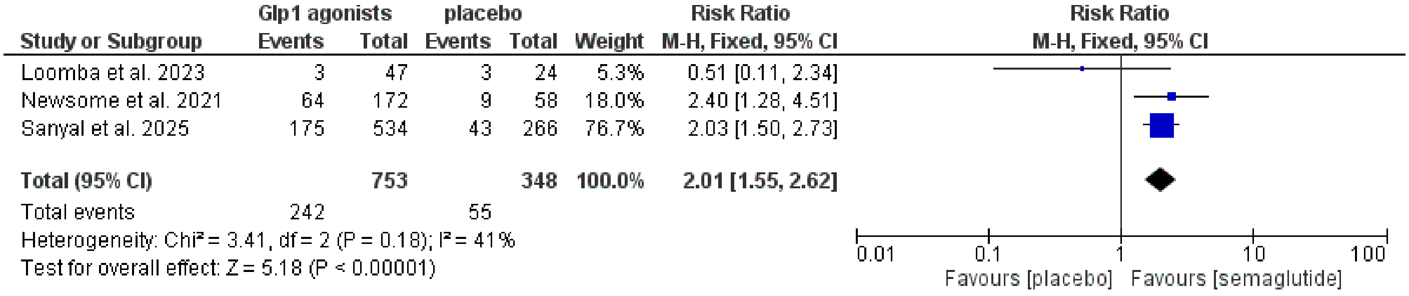

Supplementary Figure S5. Forest plot of resolution of MASH and improvement in fibrosis

Study or Subgroup	Glp1 agonists Events	Glp1 agonists Total	Control Events	Control Total	Weight	Risk Ratio M-H, Random, 95% CI
Flint et al 2021	25	34	11	33	26.6%	2.21 [1.31, 3.72]
Harrison et al. 2025	48	59	1	24	6.9%	19.53 [2.86, 133.53]
Loomba et al. 2023	23	47	3	24	15.0%	3.91 [1.31, 11.74]
Sanyal et al. 2024 a	30	35	2	8	13.4%	3.43 [1.02, 11.47]
Sanyal et al. 2024 b	136	219	10	74	25.2%	4.60 [2.56, 8.25]
Shankar et al. 2024	8	50	3	24	13.0%	1.28 [0.37, 4.40]
**Total (95% CI)**		**444**		**187**	**100.0%**	**3.32 [1.89, 5.83]**
Total events	270		30			

Heterogeneity: Tau² = 0.24; Chi² = 10.90, df = 5 (P = 0.05); I² = 54%
Test for overall effect: Z = 4.17 (P < 0.0001)

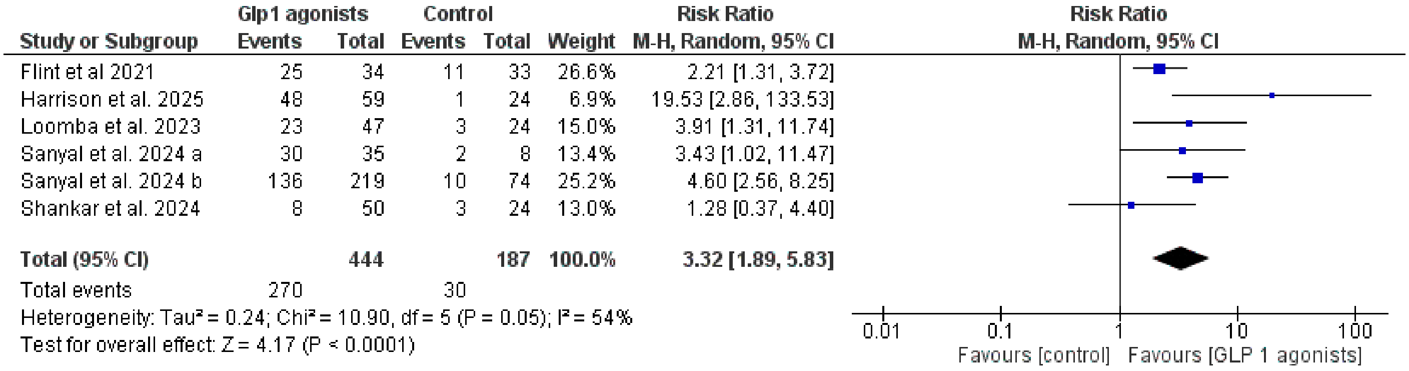

Supplementary Figure S6. Forest plot of patients with liver fat reduction ≥30%.

Study or Subgroup	Glp1 agonists Events	Glp1 agonists Total	Control Events	Control Total	Weight	Risk Ratio M-H, Random, 95% CI
Flint et al 2021	25	34	11	33	0.0%	2.21 [1.31, 3.72]
Harrison et al. 2025	48	59	1	24	8.9%	19.53 [2.86, 133.53]
Loomba et al. 2023	23	47	3	24	20.1%	3.91 [1.31, 11.74]
Sanyal et al. 2024 a	30	35	2	8	17.9%	3.43 [1.02, 11.47]
Sanyal et al. 2024 b	136	219	10	74	35.8%	4.60 [2.56, 8.25]
Shankar et al. 2024	8	50	3	24	17.3%	1.28 [0.37, 4.40]
**Total (95% CI)**		**410**		**154**	**100.0%**	**3.85 [2.05, 7.23]**
Total events	245		19			

Heterogeneity: Tau² = 0.20; Chi² = 6.66, df = 4 (P = 0.15); I² = 40%
Test for overall effect: Z = 4.19 (P < 0.0001)

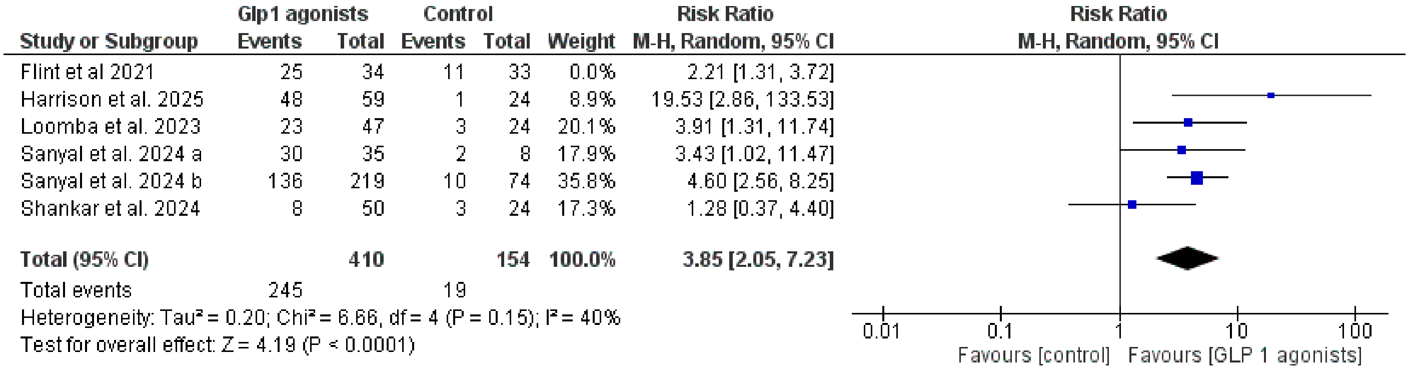

Supplementary Figure S7. Forest plot of patients with liver fat reduction ≥30% after excluding Flint et al. 2021

Study or Subgroup	Glp1 agonists Events	Glp1 agonists Total	Control Events	Control Total	Weight	Risk Ratio M-H, Random, 95% CI
Flint et al 2021	25	34	11	33	33.9%	2.21 [1.31, 3.72]
Harrison et al. 2025	48	59	1	24	0.0%	19.53 [2.86, 133.53]
Loomba et al. 2023	23	47	3	24	13.3%	3.91 [1.31, 11.74]
Sanyal et al. 2024 a	30	35	2	8	11.4%	3.43 [1.02, 11.47]
Sanyal et al. 2024 b	136	219	10	74	30.4%	4.60 [2.56, 8.25]
Shankar et al. 2024	8	50	3	24	11.0%	1.28 [0.37, 4.40]
**Total (95% CI)**		**385**		**163**	**100.0%**	**2.95 [1.88, 4.63]**
Total events	222		29			

Heterogeneity: Tau² = 0.09; Chi² = 5.98, df = 4 (P = 0.20); I² = 33%
Test for overall effect: Z = 4.69 (P < 0.00001)

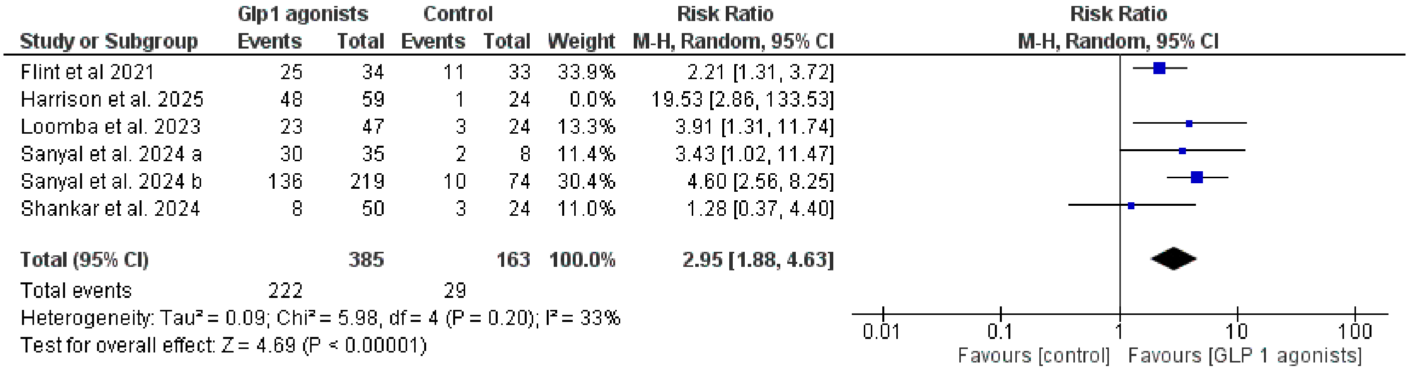

Supplementary Figure S8. Forest plot of patients with liver fat reduction ≥30% after excluding Harrison et al. 2025.

Study or Subgroup	Glp1 agonists Events	Glp1 agonists Total	Control Events	Control Total	Weight	Risk Ratio M-H, Fixed, 95% CI
Harrison et al. 2025	35	59	0	24	28.0%	29.58 [1.89, 463.68]
Sanyal et al. 2024 a	26	79	1	8	72.0%	2.63 [0.41, 16.92]
**Total (95% CI)**		**138**		**32**	**100.0%**	**10.18 [2.32, 44.68]**
Total events	61		1			

Heterogeneity: Chi² = 2.61, df = 1 (P = 0.11); I² = 62%
Test for overall effect: Z = 3.07 (P = 0.002)

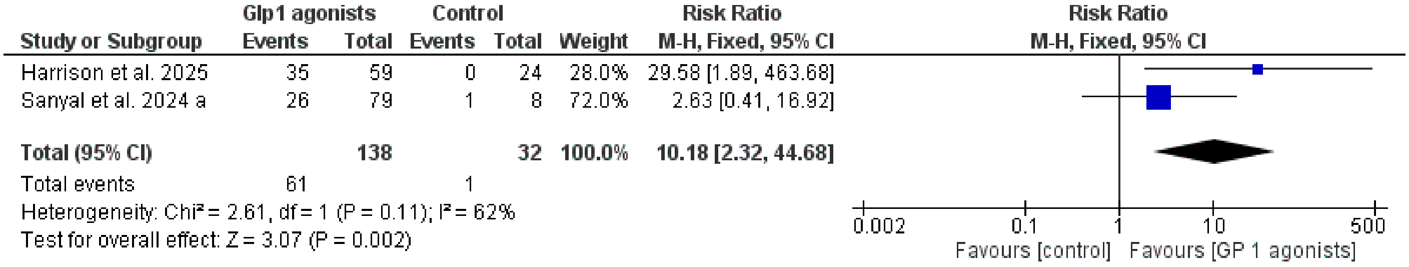

Supplementary Figure S9. Forest plot of patients with liver fat reduction ≥70% .

Study or Subgroup	Glp1 agonists Mean	SD	Total	Control Mean	SD	Total	Weight	Std. Mean Difference IV, Random, 95% CI
Bi 2014	-21.1	18.8	11	-14.6	19.2	11	4.4%	-0.33 [-1.17, 0.51]
Dutour et al. 2016	-23.8	44.6	22	12.5	45	22	5.6%	-0.80 [-1.41, -0.18]
Flint et al 2021	-14.4	15.7	34	-5.78	14.7	33	6.3%	-0.56 [-1.05, -0.07]
Guo et al. 2020	-6.3	2.9	31	-1.75	4.2	60	6.4%	-1.18 [-1.65, -0.72]
Harrison et al. 2025	-11.6	8.6	70	-0.2	7.7	24	6.2%	-1.35 [-1.85, -0.84]
Ji et al. 2025	-7	2.1	53	-0.9	1.5	22	5.1%	-3.10 [-3.81, -2.39]
Khoo et al. 2019	-7	7.1	15	-8.1	13.2	15	5.1%	0.10 [-0.62, 0.82]
Kuchay et al. 2020	-5.8	5.2	27	-2.3	6	25	5.9%	-0.62 [-1.17, -0.06]
Liu et al. 2020	-17.55	12.93	35	-10.49	11.38	36	6.3%	-0.57 [-1.05, -0.10]
Loomba et al. 2023	-3.5	7.2	47	-0.57	6.8	24	6.2%	-0.41 [-0.91, 0.09]
Loomba et al. 2024	-8.9	10.1	142	-1.8	10.3	48	7.0%	-0.70 [-1.03, -0.36]
Moolla et al. 2025	-6.2	11.6	15	-7.5	12	14	5.0%	0.11 [-0.62, 0.84]
Sanyal et al. 2024 a	-13.3	23.6	79	0.05	23.8	19	6.2%	-0.56 [-1.07, -0.05]
Sanyal et al. 2024 b	-11.4	11.1	162	-1.1	10.9	74	7.2%	-0.93 [-1.22, -0.64]
Shankar et al. 2024	-3	6.2	50	0.2	6.4	24	6.2%	-0.51 [-1.00, -0.01]
Smits et al. 2016	-2.1	19.5	17	-1.8	15.7	17	5.3%	-0.02 [-0.69, 0.66]
Yan et al. 2019	-4	4.5	24	-0.8	5.3	24	5.8%	-0.64 [-1.22, -0.06]
**Total (95% CI)**			**834**			**492**	**100.0%**	**-0.72 [-0.99, -0.45]**

Heterogeneity: Tau² = 0.24; Chi² = 73.25, df = 16 (P < 0.00001); I² = 78%
Test for overall effect: Z = 5.24 (P < 0.00001)

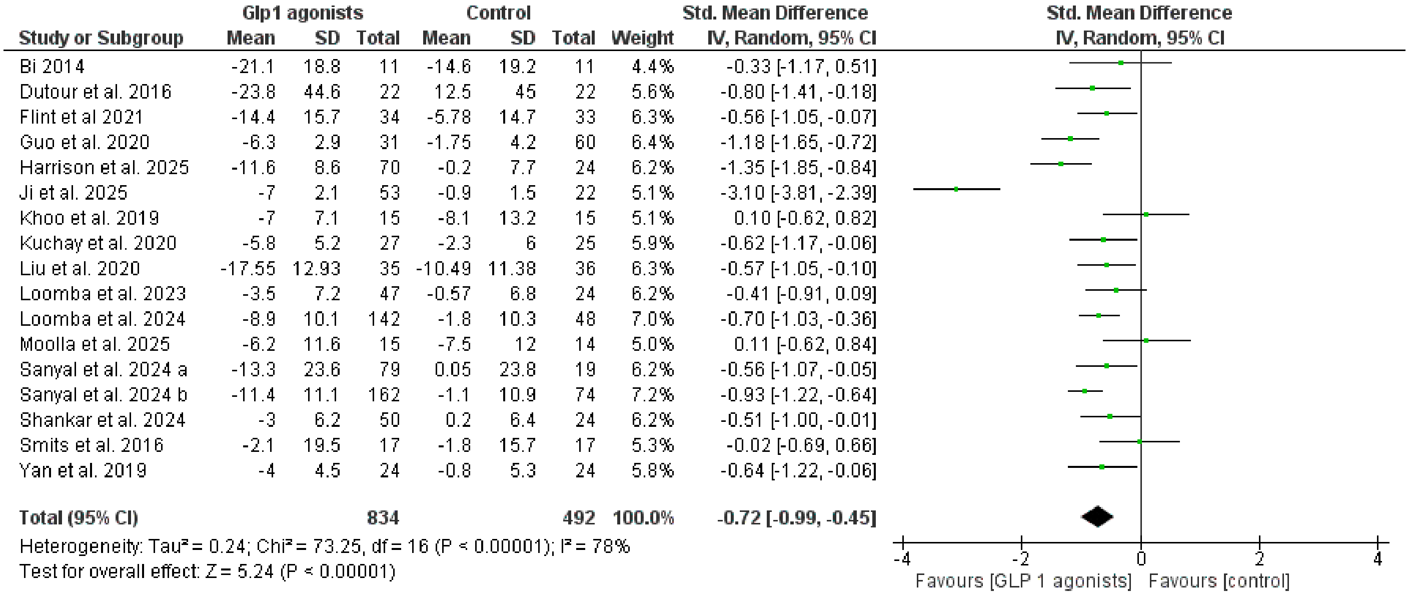

Supplementary Figure S10. Forest plot of liver fat content in the overall population .

Study or Subgroup	Glp1 agonists Mean	SD	Total	Control Mean	SD	Total	Weight	Std. Mean Difference IV, Random, 95% CI
**3.6.1 Single agonists**								
Bi 2014	-21.1	18.8	11	-14.6	19.2	11	4.4%	-0.33 [-1.17, 0.51]
Dutour et al. 2016	-23.8	44.6	22	12.5	45	22	5.6%	-0.80 [-1.41, -0.18]
Flint et al 2021	-14.4	15.7	34	-5.78	14.7	33	6.3%	-0.56 [-1.05, -0.07]
Guo et al. 2020	-6.3	2.9	31	-1.75	4.2	60	6.4%	-1.18 [-1.65, -0.72]
Ji et al. 2025	-7	2.1	53	-0.9	1.5	22	5.1%	-3.10 [-3.81, -2.39]
Khoo et al. 2019	-7	7.1	15	-8.1	13.2	15	5.1%	0.10 [-0.62, 0.82]
Kuchay et al. 2020	-5.8	5.2	27	-2.3	6	25	5.9%	-0.62 [-1.17, -0.06]
Liu et al. 2020	-17.55	12.93	35	-10.49	11.38	36	6.3%	-0.57 [-1.05, -0.10]
Loomba et al. 2023	-3.5	7.2	47	-0.57	6.8	24	6.2%	-0.41 [-0.91, 0.09]
Moolla et al. 2025	-6.2	11.6	15	-7.5	12	14	5.0%	0.11 [-0.62, 0.84]
Smits et al. 2016	-2.1	19.5	17	-1.8	15.7	17	5.3%	-0.02 [-0.69, 0.66]
Yan et al. 2019	-4	4.5	24	-0.8	5.3	24	5.8%	-0.64 [-1.22, -0.06]
**Subtotal (95% CI)**			**331**			**303**	**67.2%**	**-0.67 [-1.08, -0.26]**
Heterogeneity: Tau² = 0.43; Chi² = 64.09, df = 11 (P < 0.00001); I² = 83%								
Test for overall effect: Z = 3.20 (P = 0.001)								
**3.6.2 Dual or triple agonists**								
Harrison et al. 2025	-11.6	8.6	70	-0.2	7.7	24	6.2%	-1.35 [-1.85, -0.84]
Loomba et al. 2024	-8.9	10.1	142	-1.8	10.3	48	7.0%	-0.70 [-1.03, -0.36]
Sanyal et al. 2024 a	-13.3	23.6	79	0.05	23.8	19	6.2%	-0.56 [-1.07, -0.05]
Sanyal et al. 2024 b	-11.4	11.1	162	-1.1	10.9	74	7.2%	-0.93 [-1.22, -0.64]
Shankar et al. 2024	-3	6.2	50	0.2	6.4	24	6.2%	-0.51 [-1.00, -0.01]
**Subtotal (95% CI)**			**503**			**189**	**32.8%**	**-0.81 [-1.07, -0.56]**
Heterogeneity: Tau² = 0.04; Chi² = 7.88, df = 4 (P = 0.10); I² = 49%								
Test for overall effect: Z = 6.21 (P < 0.00001)								
**Total (95% CI)**			**834**			**492**	**100.0%**	**-0.72 [-0.99, -0.45]**

Heterogeneity: Tau² = 0.24; Chi² = 73.25, df = 16 (P < 0.00001); I² = 78%
Test for overall effect: Z = 5.24 (P < 0.00001)
Test for subgroup differences: Chi² = 0.32, df = 1 (P = 0.57), I² = 0%

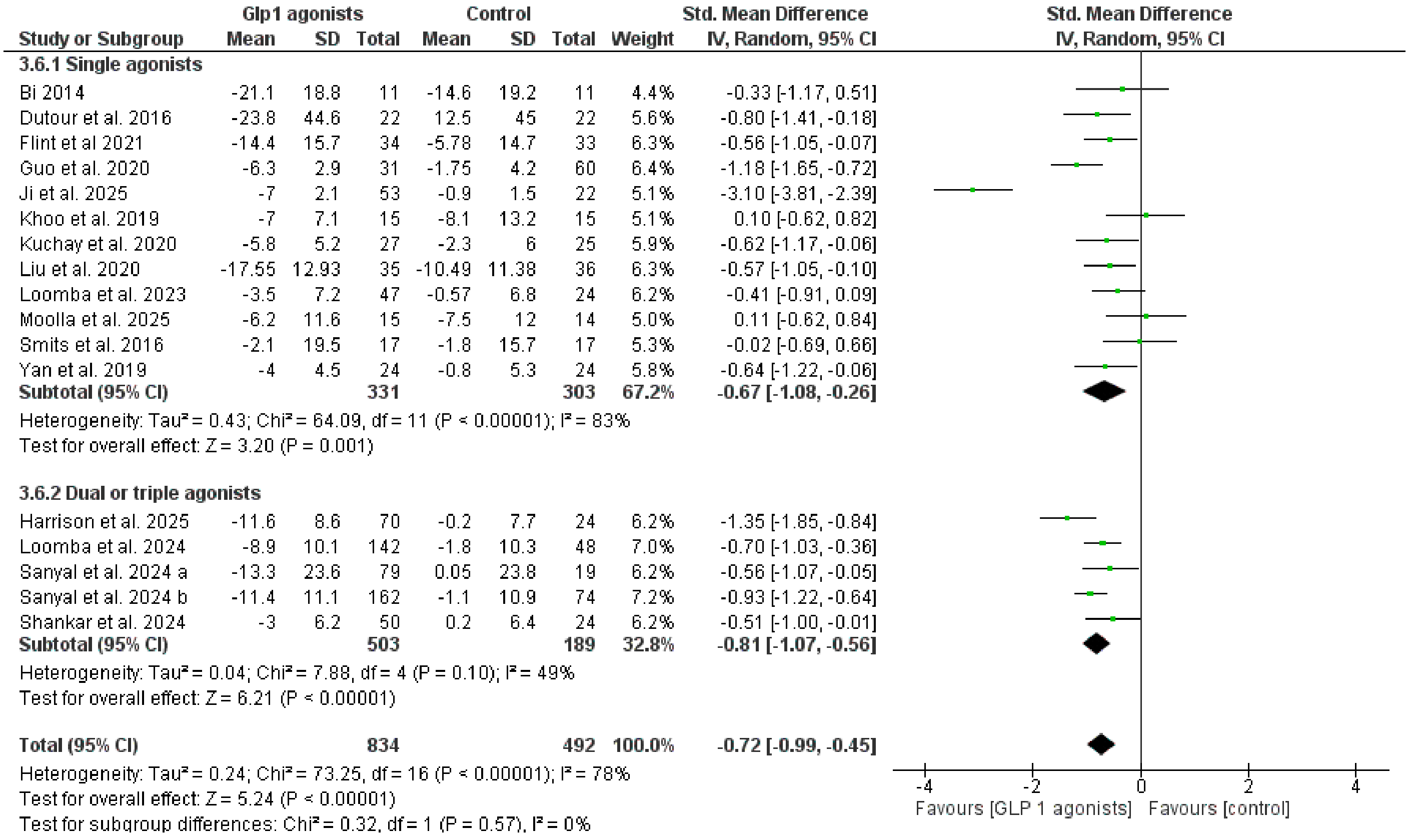

Supplementary Figure S11. Forest plot of liver fat content in patients receiving single or dual or triple agonists.

Study or Subgroup	GLP 1 agonists Mean	SD	Total	Control Mean	SD	Total	Weight	Std. Mean Difference IV, Random, 95% CI
**2.4.1 patients with type 2 DM**								
Bi 2014	-21.1	18.8	11	-14.6	19.2	11	8.0%	-0.33 [-1.17, 0.51]
Dutour et al. 2016	-23.8	44.6	22	12.5	45	22	9.2%	-0.80 [-1.41, -0.18]
Guo et al. 2020	-6.3	2.9	31	-3.4	5.6	30	9.7%	-0.65 [-1.16, -0.13]
Kuchay et al. 2020	-5.8	5.2	27	-2.3	6	25	9.5%	-0.62 [-1.17, -0.06]
Liu et al. 2020	-17.55	12.93	35	-10.49	11.38	36	9.9%	-0.57 [-1.05, -0.10]
Smits et al. 2016	-2.1	19.5	17	-1.8	15.7	17	8.9%	-0.02 [-0.69, 0.66]
Yan et al. 2019	-4	4.5	24	-0.8	5.3	24	9.4%	-0.64 [-1.22, -0.06]
**Subtotal (95% CI)**			**167**			**165**	**64.4%**	**-0.55 [-0.77, -0.33]**
Heterogeneity: Tau² = 0.00; Chi² = 3.58, df = 6 (P = 0.73); I² = 0%								
Test for overall effect: Z = 4.94 (P < 0.00001)								
**2.4.2 patients without type 2 DM**								
Ji et al. 2025	-7	2.1	53	-0.9	1.5	22	8.7%	-3.10 [-3.81, -2.39]
Khoo et al. 2019	-7	7.1	15	-8.1	13.2	15	8.6%	0.10 [-0.62, 0.82]
Moolla et al. 2025	-6.2	11.6	15	-7.5	12	14	8.6%	0.11 [-0.62, 0.84]
Sanyal et al. 2024 a	-13.3	23.6	79	0.05	23.8	19	9.7%	-0.56 [-1.07, -0.05]
**Subtotal (95% CI)**			**162**			**70**	**35.6%**	**-0.86 [-2.24, 0.52]**
Heterogeneity: Tau² = 1.87; Chi² = 53.01, df = 3 (P < 0.00001); I² = 94%								
Test for overall effect: Z = 1.22 (P = 0.22)								
**Total (95% CI)**			**329**			**235**	**100.0%**	**-0.64 [-1.09, -0.20]**
Heterogeneity: Tau² = 0.46; Chi² = 58.30, df = 10 (P < 0.00001); I² = 83%								
Test for overall effect: Z = 2.84 (P = 0.005)								
Test for subgroup differences: Chi² = 0.19, df = 1 (P = 0.67), I² = 0%								

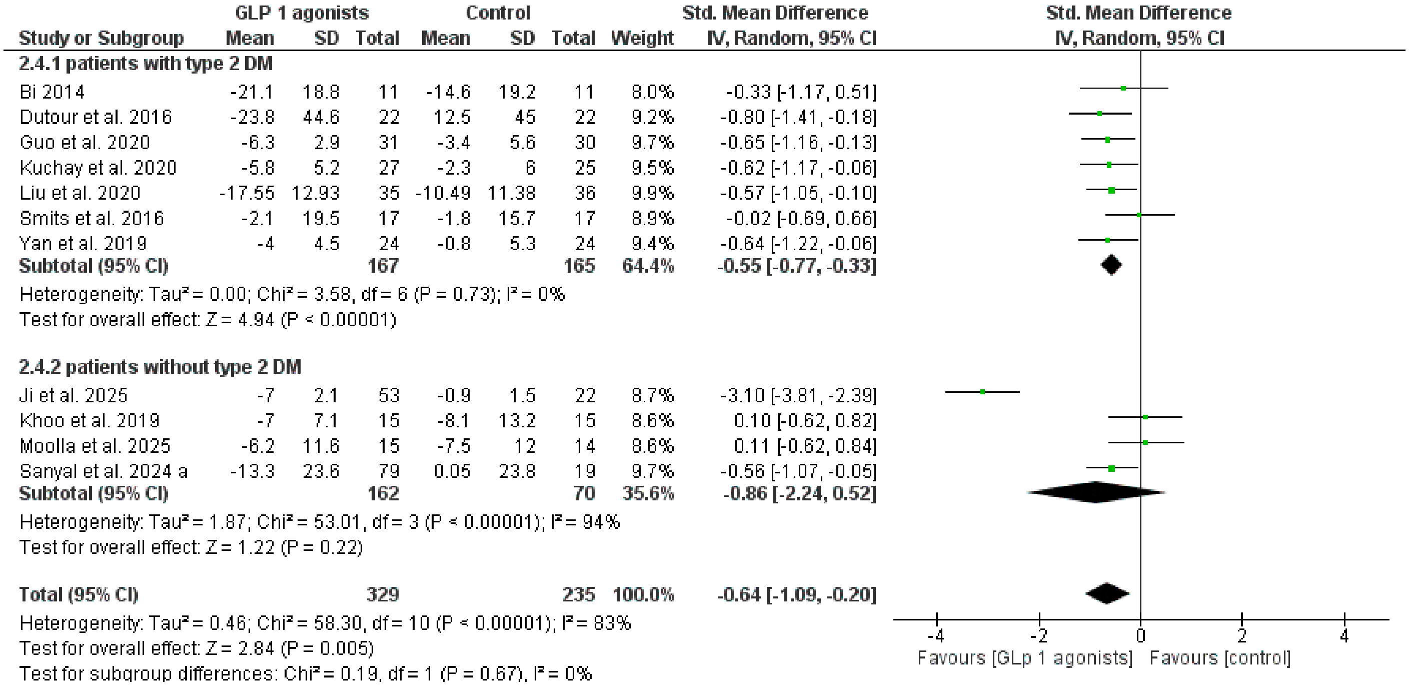

Supplementary Figure S12. Forest plot of liver fat content in patients with and without type 2 diabetes.

Study or Subgroup	GLP 1 agonists Mean	SD	Total	Control Mean	SD	Total	Weight	Std. Mean Difference IV, Random, 95% CI
**2.4.1 patients with type 2 DM**								
Bi 2014	-21.1	18.8	11	-14.6	19.2	11	5.0%	-0.33 [-1.17, 0.51]
Dutour et al. 2016	-23.8	44.6	22	12.5	45	22	9.3%	-0.80 [-1.41, -0.18]
Guo et al. 2020	-6.3	2.9	31	-3.4	5.6	30	13.2%	-0.65 [-1.16, -0.13]
Kuchay et al. 2020	-5.8	5.2	27	-2.3	6	25	11.3%	-0.62 [-1.17, -0.06]
Liu et al. 2020	-17.55	12.93	35	-10.49	11.38	36	15.5%	-0.57 [-1.05, -0.10]
Smits et al. 2016	-2.1	19.5	17	-1.8	15.7	17	7.8%	-0.02 [-0.69, 0.66]
Yan et al. 2019	-4	4.5	24	-0.8	5.3	24	10.5%	-0.64 [-1.22, -0.06]
**Subtotal (95% CI)**			**167**			**165**	**72.7%**	**-0.55 [-0.77, -0.33]**
Heterogeneity: Tau² = 0.00; Chi² = 3.58, df = 6 (P = 0.73); I² = 0%								
Test for overall effect: Z = 4.94 (P < 0.00001)								
**2.4.2 patients without type 2 DM**								
Ji et al. 2025	-7	2.1	53	-0.9	1.5	22	0.0%	-3.10 [-3.81, -2.39]
Khoo et al. 2019	-7	7.1	15	-8.1	13.2	15	6.9%	0.10 [-0.62, 0.82]
Moolla et al. 2025	-6.2	11.6	15	-7.5	12	14	6.7%	0.11 [-0.62, 0.84]
Sanyal et al. 2024 a	-13.3	23.6	79	0.05	23.8	19	13.7%	-0.56 [-1.07, -0.05]
**Subtotal (95% CI)**			**109**			**48**	**27.3%**	**-0.18 [-0.66, 0.29]**
Heterogeneity: Tau² = 0.07; Chi² = 3.27, df = 2 (P = 0.19); I² = 39%								
Test for overall effect: Z = 0.75 (P = 0.45)								
**Total (95% CI)**			**276**			**213**	**100.0%**	**-0.47 [-0.65, -0.28]**
Heterogeneity: Tau² = 0.00; Chi² = 9.12, df = 9 (P = 0.43); I² = 1%								
Test for overall effect: Z = 4.82 (P < 0.00001)								
Test for subgroup differences: Chi² = 1.96, df = 1 (P = 0.16), I² = 49.0%								

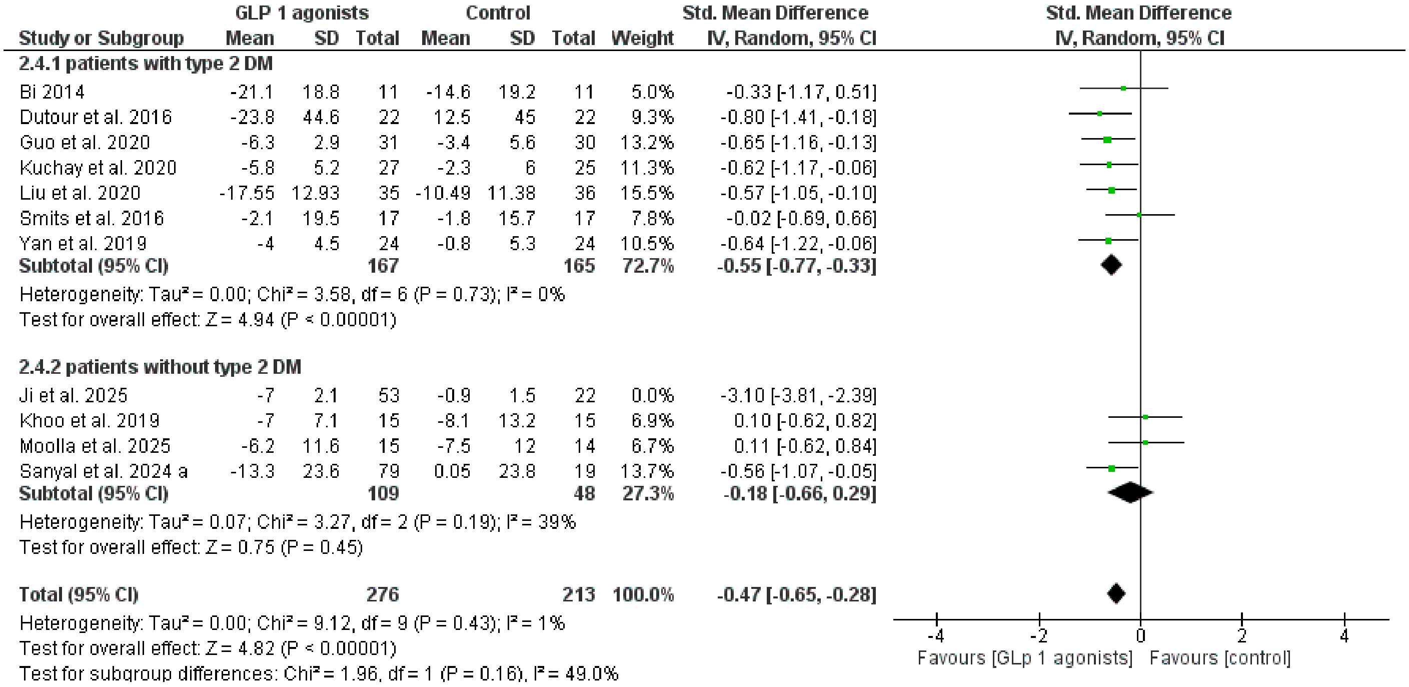

Supplementary Figure S13. Forest plot of liver fat content in patients with and without type 2 diabetes after leaving Ji et al.

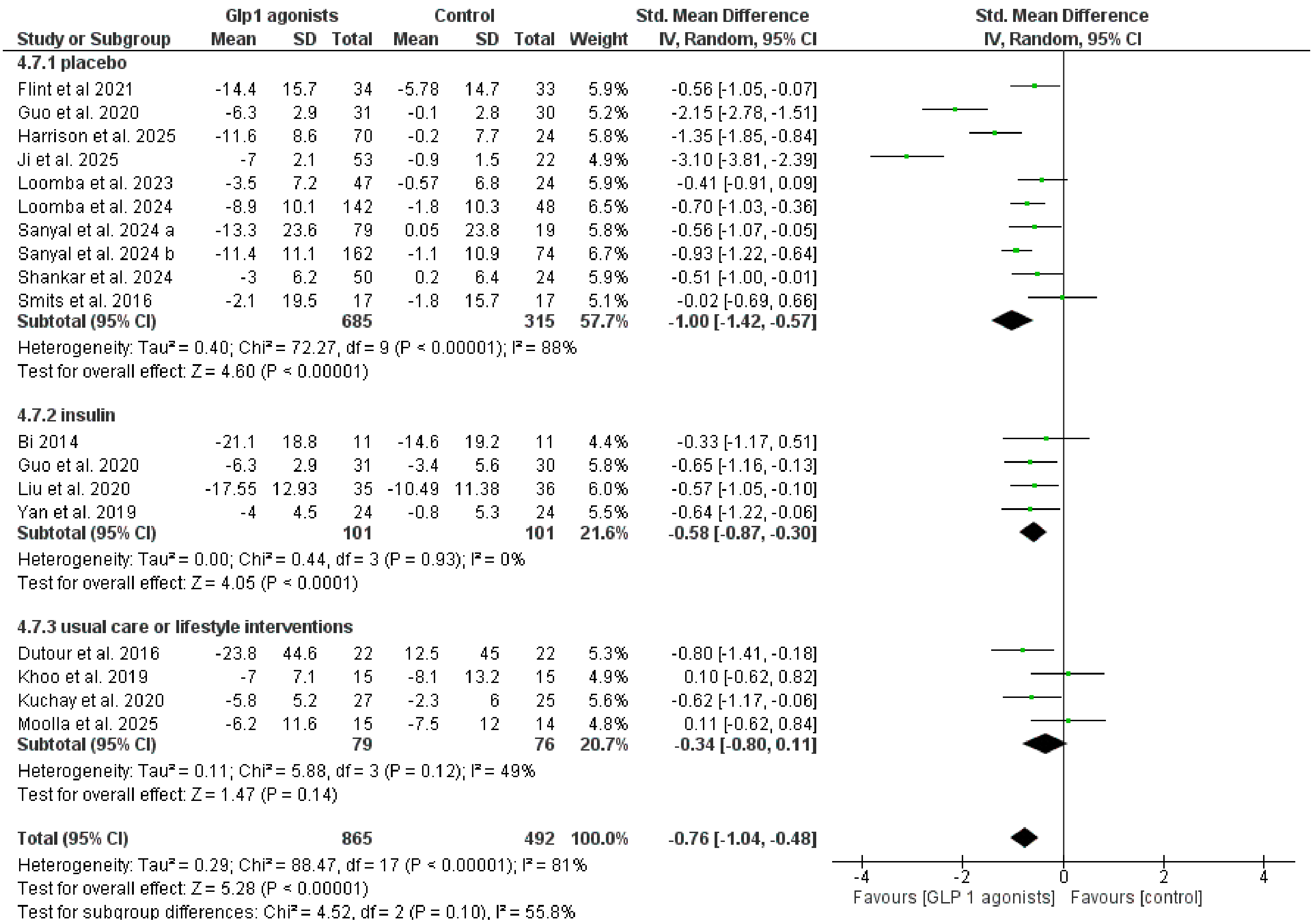

Study or Subgroup	Glp1 agonists Mean	SD	Total	Control Mean	SD	Total	Weight	Std. Mean Difference IV, Random, 95% CI
**4.7.1 placebo**								
Flint et al 2021	-14.4	15.7	34	-5.78	14.7	33	5.9%	-0.56 [-1.05, -0.07]
Guo et al. 2020	-6.3	2.9	31	-0.1	2.8	30	5.2%	-2.15 [-2.78, -1.51]
Harrison et al. 2025	-11.6	8.6	70	-0.2	7.7	24	5.8%	-1.35 [-1.85, -0.84]
Ji et al. 2025	-7	2.1	53	-0.9	1.5	22	4.9%	-3.10 [-3.81, -2.39]
Loomba et al. 2023	-3.5	7.2	47	-0.57	6.8	24	5.9%	-0.41 [-0.91, 0.09]
Loomba et al. 2024	-8.9	10.1	142	-1.8	10.3	48	6.5%	-0.70 [-1.03, -0.36]
Sanyal et al. 2024 a	-13.3	23.6	79	0.05	23.8	19	5.8%	-0.56 [-1.07, -0.05]
Sanyal et al. 2024 b	-11.4	11.1	162	-1.1	10.9	74	6.7%	-0.93 [-1.22, -0.64]
Shankar et al. 2024	-3	6.2	50	0.2	6.4	24	5.9%	-0.51 [-1.00, -0.01]
Smits et al. 2016	-2.1	19.5	17	-1.8	15.7	17	5.1%	-0.02 [-0.69, 0.66]
**Subtotal (95% CI)**			**685**			**315**	**57.7%**	**-1.00 [-1.42, -0.57]**
Heterogeneity: Tau² = 0.40; Chi² = 72.27, df = 9 (P < 0.00001); I² = 88%								
Test for overall effect: Z = 4.60 (P < 0.00001)								
**4.7.2 insulin**								
Bi 2014	-21.1	18.8	11	-14.6	19.2	11	4.4%	-0.33 [-1.17, 0.51]
Guo et al. 2020	-6.3	2.9	31	-3.4	5.6	30	5.8%	-0.65 [-1.16, -0.13]
Liu et al. 2020	-17.55	12.93	35	-10.49	11.38	36	6.0%	-0.57 [-1.05, -0.10]
Yan et al. 2019	-4	4.5	24	-0.8	5.3	24	5.5%	-0.64 [-1.22, -0.06]
**Subtotal (95% CI)**			**101**			**101**	**21.6%**	**-0.58 [-0.87, -0.30]**
Heterogeneity: Tau² = 0.00; Chi² = 0.44, df = 3 (P = 0.93); I² = 0%								
Test for overall effect: Z = 4.05 (P < 0.0001)								
**4.7.3 usual care or lifestyle interventions**								
Dutour et al. 2016	-23.8	44.6	22	12.5	45	22	5.3%	-0.80 [-1.41, -0.18]
Khoo et al. 2019	-7	7.1	15	-8.1	13.2	15	4.9%	0.10 [-0.62, 0.82]
Kuchay et al. 2020	-5.8	5.2	27	-2.3	6	25	5.6%	-0.62 [-1.17, -0.06]
Moolla et al. 2025	-6.2	11.6	15	-7.5	12	14	4.8%	0.11 [-0.62, 0.84]
**Subtotal (95% CI)**			**79**			**76**	**20.7%**	**-0.34 [-0.80, 0.11]**
Heterogeneity: Tau² = 0.11; Chi² = 5.88, df = 3 (P = 0.12); I² = 49%								
Test for overall effect: Z = 1.47 (P = 0.14)								
**Total (95% CI)**			**865**			**492**	**100.0%**	**-0.76 [-1.04, -0.48]**
Heterogeneity: Tau² = 0.29; Chi² = 88.47, df = 17 (P < 0.00001); I² = 81%								
Test for overall effect: Z = 5.28 (P < 0.00001)								
Test for subgroup differences: Chi² = 4.52, df = 2 (P = 0.10), I² = 55.8%								

Supplementary Figure S14. Forest plot of liver fat content in based on the control group.

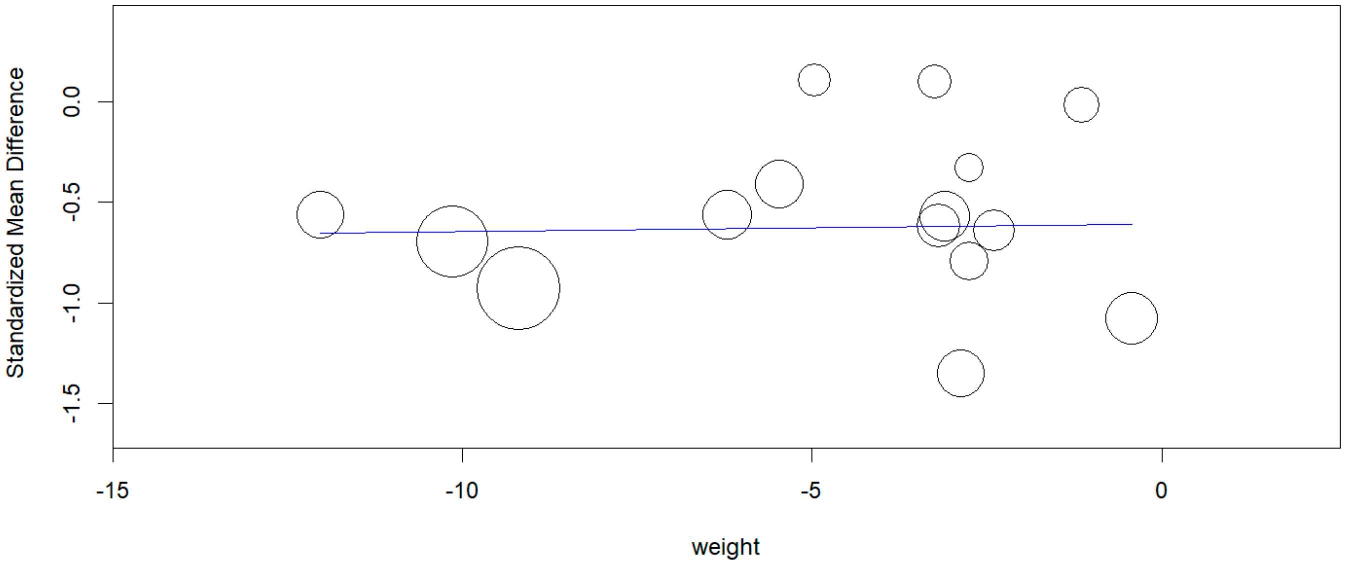

Supplementary Figure S15. Meta-regression showing no significant association between weight reduction and GLP-1 agonists reduction of liver fat content.

Study or Subgroup	Glp1 agonists Mean	SD	Total	Control Mean	SD	Total	Weight	Std. Mean Difference IV, Random, 95% CI
Armstrong et al. 2016	-5.3	4.7	23	-0.6	4.4	22	5.5%	-1.01 [-1.64, -0.39]
Bi 2014	-4.6	14.9	11	-0.9	9.8	11	5.0%	-0.28 [-1.12, 0.56]
Dutour et al. 2016	-5.3	4.7	22	-0.2	3.8	22	5.4%	-1.17 [-1.82, -0.53]
Flint et al 2021	-11.1	12.3	34	-1.2	12	33	5.7%	-0.81 [-1.30, -0.31]
Guo et al. 2020	-5.1	4.3	31	-0.75	3	60	5.7%	-1.23 [-1.71, -0.76]
Harrison et al. 2025	-3.8	3.1	70	-0.2	3.1	24	5.7%	-1.15 [-1.64, -0.66]
Khoo et al. 2019	-3	2.2	15	-3.5	3.3	15	5.3%	0.17 [-0.54, 0.89]
Kuchay et al. 2020	-4.3	2.6	27	-2	3.5	25	5.6%	-0.74 [-1.30, -0.18]
Liu et al. 2020	-5	3.55	35	-1.25	3.73	36	5.7%	-1.02 [-1.51, -0.52]
Loomba et al. 2023	-8.3	25.3	47	0.1	30.3	24	5.7%	-0.31 [-0.80, 0.19]
Loomba et al. 2024	-13.3	2	142	-0.8	0.3	48	5.1%	-7.16 [-7.96, -6.36]
Moolla et al. 2025	-5.2	30.1	15	-4.7	29.6	14	5.3%	-0.02 [-0.74, 0.71]
Newsome et al. 2021	-8.9	31.1	240	-1.38	32.8	80	6.0%	-0.24 [-0.49, 0.02]
Sanyal et al. 2024 a	-14.9	7.5	79	-0.11	3.38	19	5.5%	-2.12 [-2.71, -1.54]
Sanyal et al. 2024 b	-12.1	8.5	219	-0.6	3.71	74	5.9%	-1.51 [-1.80, -1.22]
Sanyal et al. 2025	-10	7.13	534	-2	7.3	266	6.0%	-1.11 [-1.27, -0.96]
Smits et al. 2016	-2.2	18.4	17	-0.1	14.3	17	5.4%	-0.12 [-0.80, 0.55]
Yan et al. 2019	-3.6	4.9	24	-1.2	4.2	24	5.5%	-0.52 [-1.09, 0.06]
**Total (95% CI)**			**1585**			**814**	**100.0%**	**-1.11 [-1.57, -0.66]**

Heterogeneity: Tau² = 0.89; Chi² = 331.52, df = 17 (P < 0.00001); I² = 95%

Test for overall effect: Z = 4.80 (P < 0.00001)

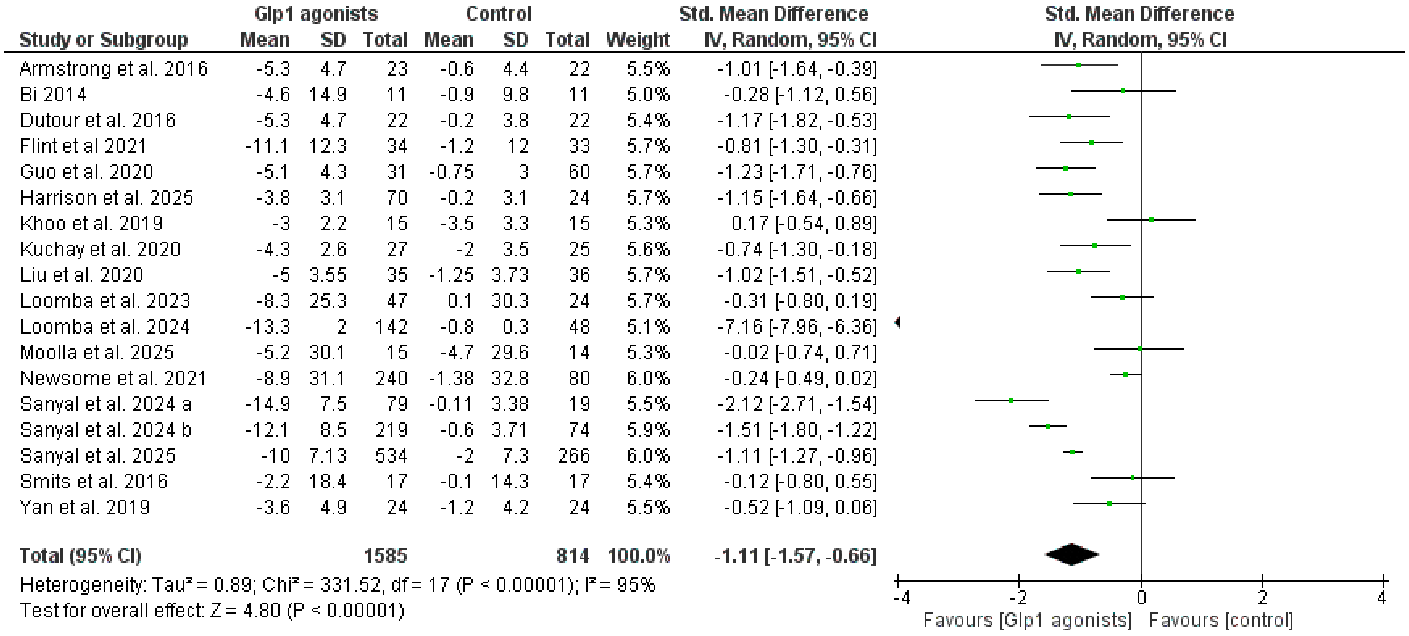

Supplementary Figure S16. Forest plot of change in weight in the overall population.

Study or Subgroup	Glp1 agonists Mean	SD	Total	Control Mean	SD	Total	Weight	Std. Mean Difference IV, Random, 95% CI
Armstrong et al. 2016	-0.53	0.64	23	0	0.8	22	6.6%	-0.72 [-1.33, -0.12]
Bi 2014	-2.9	1.3	11	-2.8	1.2	11	5.6%	-0.08 [-0.91, 0.76]
Dutour et al. 2016	-0.7	1.4	22	-0.7	1.8	22	6.6%	0.00 [-0.59, 0.59]
Flint et al 2021	-1.03	0.1	34	-0.03	0.1	33	2.6%	-9.88 [-11.67, -8.09]
Guo et al. 2020	-0.7	1.1	31	-0.3	0.8	60	7.2%	-0.43 [-0.87, 0.00]
Harrison et al. 2025	0.2	1.5	70	0.2	1.7	24	7.1%	0.00 [-0.46, 0.46]
Kuchay et al. 2020	-1.6	1	27	-1.3	1	25	6.8%	-0.30 [-0.84, 0.25]
Liu et al. 2020	-2.28	1.01	35	-1.82	1.09	36	7.1%	-0.43 [-0.90, 0.04]
Loomba et al. 2023	-0.98	3.7	47	0.6	1.9	24	7.0%	-0.49 [-0.98, 0.01]
Loomba et al. 2024	-1.4	1.3	125	-0.1	1.2	38	7.4%	-1.01 [-1.39, -0.63]
Newsome et al. 2021	-1	1.2	149	-0.01	1.2	50	7.6%	-0.82 [-1.15, -0.49]
Sanyal et al. 2024 b	-0.7	0.7	219	0.14	0.7	74	7.7%	-1.20 [-1.48, -0.92]
Sanyal et al. 2025	-0.42	0.58	534	0.11	0.58	266	8.0%	-0.91 [-1.07, -0.76]
Smits et al. 2016	-0.7	1.1	17	0.5	1.4	17	6.1%	-0.93 [-1.64, -0.22]
Yan et al. 2019	-1	0.9	24	-0.7	1.3	24	6.7%	-0.26 [-0.83, 0.30]
**Total (95% CI)**			**1368**			**726**	**100.0%**	**-0.81 [-1.16, -0.45]**

Heterogeneity: Tau² = 0.40; Chi² = 144.59, df = 14 (P < 0.00001); I² = 90%

Test for overall effect: Z = 4.45 (P < 0.00001)

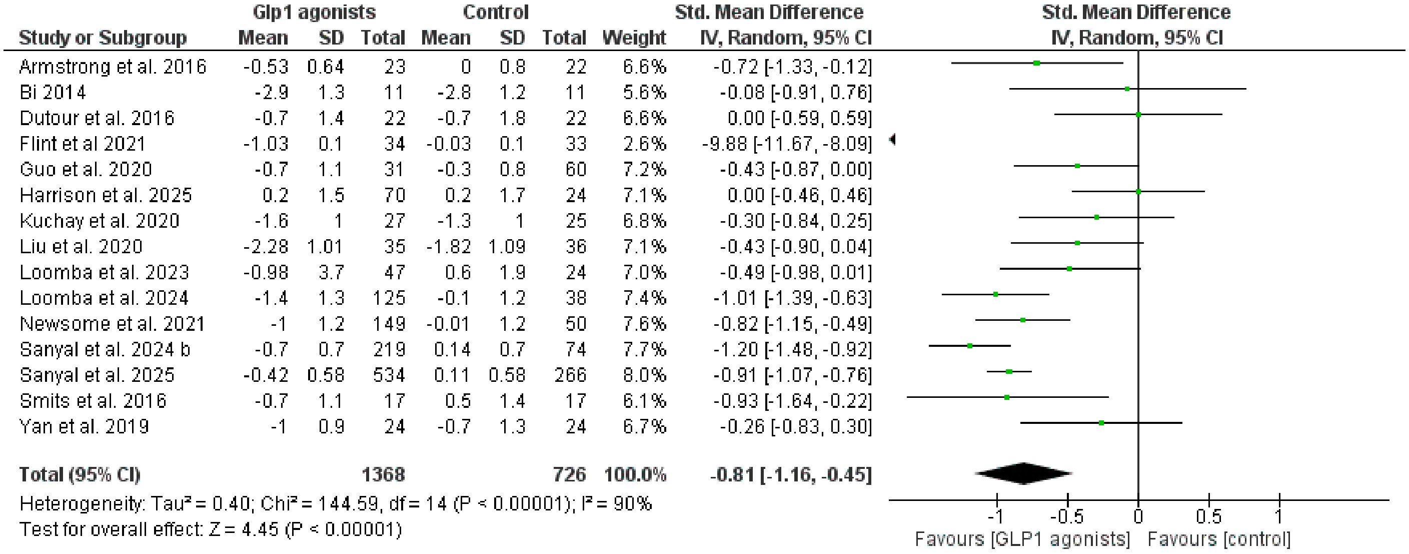

Supplementary Figure S17. Forest plot of change in HbA1c in the overall population.

	GLP 1 agonists			placebo				Std. Mean Difference
Study or Subgroup	Mean	SD	Total	Mean	SD	Total	Weight	IV, Random, 95% CI
**2.3.1 patients with type 2 DM**								
Armstrong et al. 2024	-11.1	6.2	108	-0.8	6.2	88	9.7%	-1.65 [-1.98, -1.33]
Bi 2014	-4.6	14.9	11	-0.9	9.8	11	7.2%	-0.28 [-1.12, 0.56]
Dutour et al. 2016	-5.3	4.7	22	-0.2	3.8	22	8.3%	-1.17 [-1.82, -0.53]
Guo et al. 2020	-5.1	4.3	31	-0.6	2.3	30	8.7%	-1.28 [-1.84, -0.73]
Kuchay et al. 2020	-4.3	2.6	27	-2	3.5	25	8.7%	-0.74 [-1.30, -0.18]
Liu et al. 2020	-5	3.55	35	-1.25	3.73	36	9.0%	-1.02 [-1.51, -0.52]
Smits et al. 2016	-2.2	18.4	17	-0.1	14.3	17	8.1%	-0.12 [-0.80, 0.55]
Yan et al. 2019	-3.6	4.9	24	-1.2	4.2	24	8.6%	-0.52 [-1.09, 0.06]
**Subtotal (95% CI)**			**275**			**253**	**68.3%**	**-0.90 [-1.29, -0.50]**
Heterogeneity: Tau² = 0.24; Chi² = 28.83, df = 7 (P = 0.0002); I² = 76%								
Test for overall effect: Z = 4.43 (P < 0.00001)								
**2.3.2 patients without type 2 diabetes**								
Armstrong et al. 2024	-12.7	6	21	-1.1	6	15	7.4%	-1.89 [-2.70, -1.08]
Khoo et al. 2019	-3	2.2	15	-3.5	3.3	15	7.9%	0.17 [-0.54, 0.89]
Moolla et al. 2025	-5.2	30.1	15	-4.7	29.6	14	7.8%	-0.02 [-0.74, 0.71]
Sanyal et al. 2024 a	-14.9	7.5	79	-0.11	3.38	19	8.6%	-2.12 [-2.71, -1.54]
**Subtotal (95% CI)**			**130**			**63**	**31.7%**	**-0.97 [-2.18, 0.25]**
Heterogeneity: Tau² = 1.41; Chi² = 35.93, df = 3 (P < 0.00001); I² = 92%								
Test for overall effect: Z = 1.56 (P = 0.12)								
**Total (95% CI)**			**405**			**316**	**100.0%**	**-0.91 [-1.32, -0.50]**
Heterogeneity: Tau² = 0.43; Chi² = 64.78, df = 11 (P < 0.00001); I² = 83%								
Test for overall effect: Z = 4.32 (P < 0.0001)								
Test for subgroup differences: Chi² = 0.01, df = 1 (P = 0.91), I² = 0%								

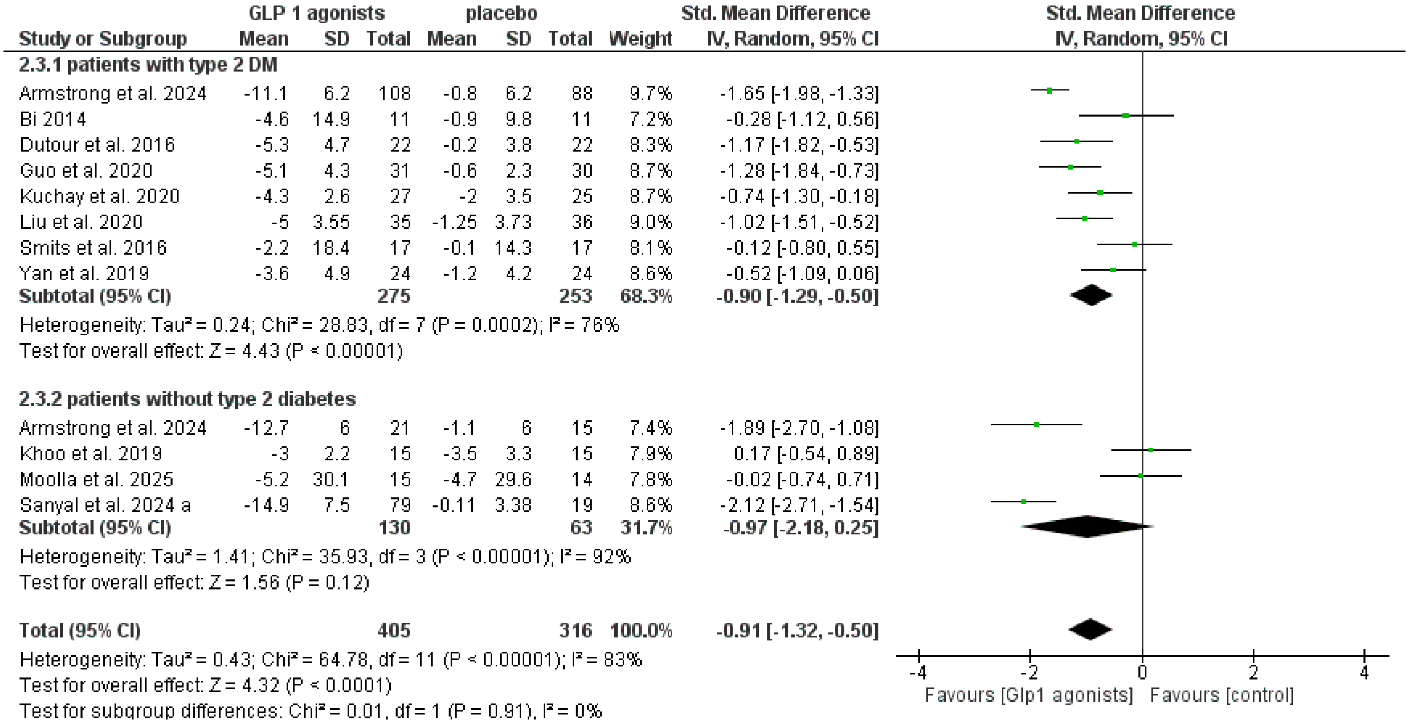

Supplementary Figure S18. Forest plot of change in weight in patients with and without type 2 diabetes.

Study or Subgroup	Glp1 agonists Mean	SD	Total	Control Mean	SD	Total	Weight	Std. Mean Difference IV, Random, 95% CI
**4.4.1 usual care or lifestyle interventions**								
Dutour et al. 2016	-5.3	4.7	22	-0.2	3.8	22	5.1%	-1.17 [-1.82, -0.53]
Khoo et al. 2019	-3	2.2	15	-3.5	3.3	15	5.0%	0.17 [-0.54, 0.89]
Kuchay et al. 2020	-4.3	2.6	27	-2	3.5	25	5.3%	-0.74 [-1.30, -0.18]
Moolla et al. 2025	-5.2	30.1	15	-4.7	29.6	14	5.0%	-0.02 [-0.74, 0.71]
**Subtotal (95% CI)**			**79**			**76**	**20.4%**	**-0.46 [-1.06, 0.14]**
Heterogeneity: Tau² = 0.26; Chi² = 9.94, df = 3 (P = 0.02); I² = 70%								
Test for overall effect: Z = 1.51 (P = 0.13)								
**4.4.2 placebo**								
Armstrong et al. 2016	-5.3	4.7	23	-0.6	4.4	22	5.2%	-1.01 [-1.64, -0.39]
Flint et al 2021	-11.1	12.3	34	-1.2	12	33	5.4%	-0.81 [-1.30, -0.31]
Guo et al. 2020	-5.1	4.3	31	-0.6	2.3	30	5.3%	-1.28 [-1.84, -0.73]
Harrison et al. 2025	-3.8	3.1	70	-0.2	3.1	24	5.4%	-1.15 [-1.64, -0.66]
Loomba et al. 2023	-8.3	25.3	47	0.1	30.3	24	5.4%	-0.31 [-0.80, 0.19]
Loomba et al. 2024	-13.3	2	142	-0.8	0.3	48	4.9%	-7.16 [-7.96, -6.36]
Newsome et al. 2021	-8.9	31.1	240	-1.38	32.8	80	5.7%	-0.24 [-0.49, 0.02]
Sanyal et al. 2024 a	-14.9	7.5	79	-0.11	3.38	19	5.2%	-2.12 [-2.71, -1.54]
Sanyal et al. 2024 b	-12.1	8.5	219	-0.6	3.71	74	5.6%	-1.51 [-1.80, -1.22]
Sanyal et al. 2025	-10	7.13	534	-2	7.3	266	5.7%	-1.11 [-1.27, -0.96]
Smits et al. 2016	-2.2	18.4	17	-0.1	14.3	17	5.1%	-0.12 [-0.80, 0.55]
**Subtotal (95% CI)**			**1436**			**637**	**58.9%**	**-1.49 [-2.13, -0.85]**
Heterogeneity: Tau² = 1.10; Chi² = 305.22, df = 10 (P < 0.00001); I² = 97%								
Test for overall effect: Z = 4.56 (P < 0.00001)								
**4.4.3 insulin**								
Bi 2014	-4.6	14.9	11	-0.9	9.8	11	4.8%	-0.28 [-1.12, 0.56]
Guo et al. 2020	-5.1	4.3	31	-0.9	3.6	30	5.3%	-1.04 [-1.58, -0.51]
Liu et al. 2020	-5	3.55	35	-1.25	3.73	36	5.4%	-1.02 [-1.51, -0.52]
Yan et al. 2019	-3.6	4.9	24	-1.2	4.2	24	5.3%	-0.52 [-1.09, 0.06]
**Subtotal (95% CI)**			**101**			**101**	**20.7%**	**-0.80 [-1.13, -0.46]**
Heterogeneity: Tau² = 0.03; Chi² = 3.91, df = 3 (P = 0.27); I² = 23%								
Test for overall effect: Z = 4.65 (P < 0.00001)								
**Total (95% CI)**			**1616**			**814**	**100.0%**	**-1.11 [-1.55, -0.68]**
Heterogeneity: Tau² = 0.86; Chi² = 331.59, df = 18 (P < 0.00001); I² = 95%								
Test for overall effect: Z = 4.99 (P < 0.00001)								
Test for subgroup differences: Chi² = 5.53, df = 2 (P = 0.06), I² = 63.8%								

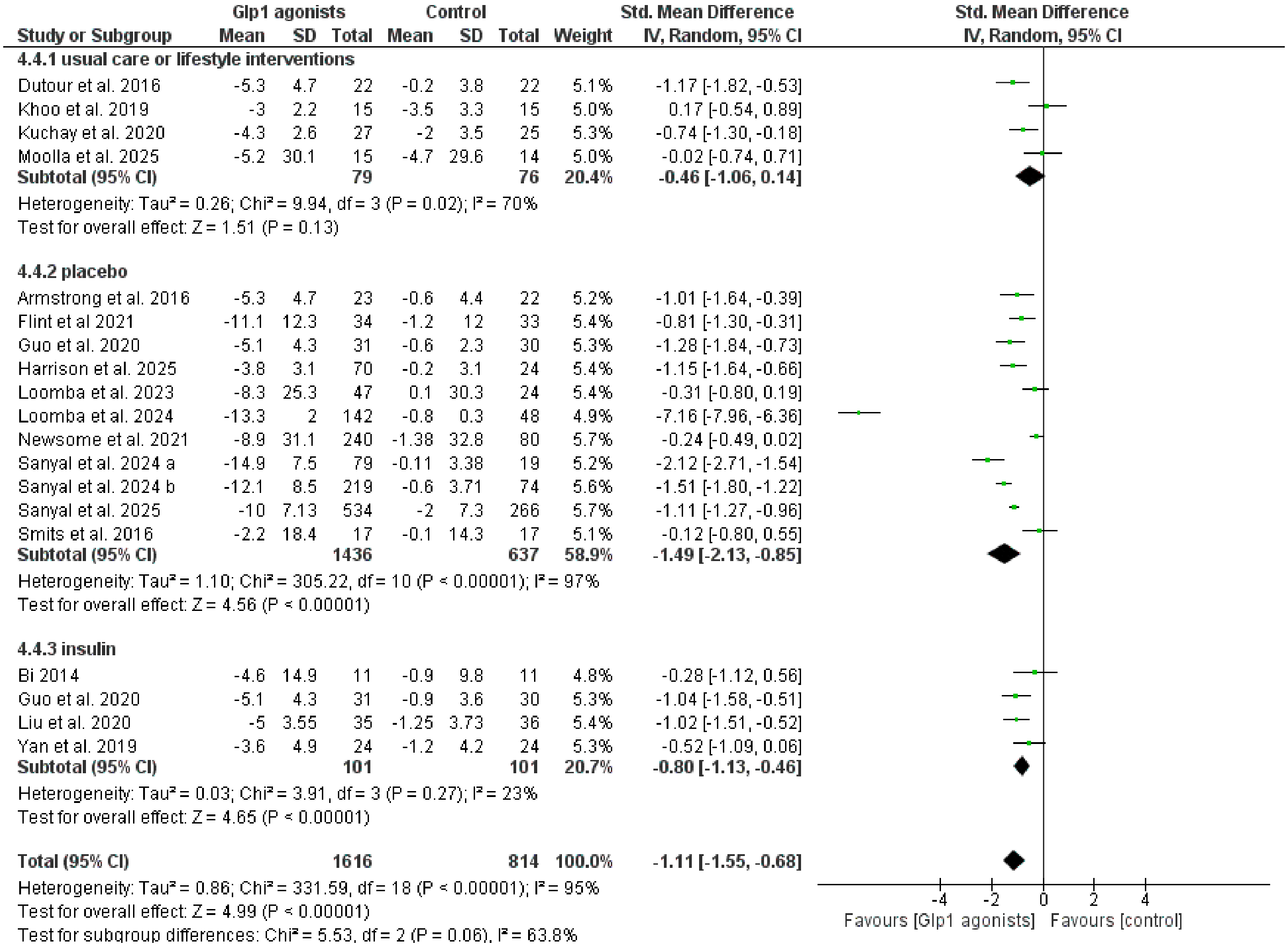

Supplementary Figure S19. Forest plot of change in weight based on the control group.

	Glp1 agonists			Control				Std. Mean Difference
Study or Subgroup	Mean	SD	Total	Mean	SD	Total	Weight	IV, Random, 95% CI
**4.4.1 usual care or lifestyle interventions**								
Dutour et al. 2016	-5.3	4.7	22	-0.2	3.8	22		Not estimable
Khoo et al. 2019	-3	2.2	15	-3.5	3.3	15	5.3%	0.17 [-0.54, 0.89]
Kuchay et al. 2020	-4.3	2.6	27	-2	3.5	25	5.6%	-0.74 [-1.30, -0.18]
Moolla et al. 2025	-5.2	30.1	15	-4.7	29.6	14	5.3%	-0.02 [-0.74, 0.71]
**Subtotal (95% CI)**			**57**			**54**	**16.1%**	**-0.23 [-0.81, 0.35]**
Heterogeneity: Tau² = 0.15; Chi² = 4.58, df = 2 (P = 0.10); I² = 56%								
Test for overall effect: Z = 0.78 (P = 0.43)								
**4.4.2 placebo**								
Armstrong et al. 2016	-5.3	4.7	23	-0.6	4.4	22	5.5%	-1.01 [-1.64, -0.39]
Flint et al 2021	-11.1	12.3	34	-1.2	12	33	5.7%	-0.81 [-1.30, -0.31]
Guo et al. 2020	-5.1	4.3	31	-0.6	2.3	30	5.6%	-1.28 [-1.84, -0.73]
Harrison et al. 2025	-3.8	3.1	70	-0.2	3.1	24	5.7%	-1.15 [-1.64, -0.66]
Loomba et al. 2023	-8.3	25.3	47	0.1	30.3	24	5.7%	-0.31 [-0.80, 0.19]
Loomba et al. 2024	-13.3	2	142	-0.8	0.3	48	5.1%	-7.16 [-7.96, -6.36]
Newsome et al. 2021	-8.9	31.1	240	-1.38	32.8	80	6.0%	-0.24 [-0.49, 0.02]
Sanyal et al. 2024 a	-14.9	7.5	79	-0.11	3.38	19	5.5%	-2.12 [-2.71, -1.54]
Sanyal et al. 2024 b	-12.1	8.5	219	-0.6	3.71	74	5.9%	-1.51 [-1.80, -1.22]
Sanyal et al. 2025	-10	7.13	534	-2	7.3	266	6.0%	-1.11 [-1.27, -0.96]
Smits et al. 2016	-2.2	18.4	17	-0.1	14.3	17	5.4%	-0.12 [-0.80, 0.55]
**Subtotal (95% CI)**			**1436**			**637**	**62.0%**	**-1.49 [-2.13, -0.85]**
Heterogeneity: Tau² = 1.10; Chi² = 305.22, df = 10 (P < 0.00001); I² = 97%								
Test for overall effect: Z = 4.56 (P < 0.00001)								
**4.4.3 insulin**								
Bi 2014	-4.6	14.9	11	-0.9	9.8	11	5.0%	-0.28 [-1.12, 0.56]
Guo et al. 2020	-5.1	4.3	31	-0.9	3.6	30	5.6%	-1.04 [-1.58, -0.51]
Liu et al. 2020	-5	3.55	35	-1.25	3.73	36	5.7%	-1.02 [-1.51, -0.52]
Yan et al. 2019	-3.6	4.9	24	-1.2	4.2	24	5.5%	-0.52 [-1.09, 0.06]
**Subtotal (95% CI)**			**101**			**101**	**21.9%**	**-0.80 [-1.13, -0.46]**
Heterogeneity: Tau² = 0.03; Chi² = 3.91, df = 3 (P = 0.27); I² = 23%								
Test for overall effect: Z = 4.65 (P < 0.00001)								
**Total (95% CI)**			**1594**			**792**	**100.0%**	**-1.11 [-1.56, -0.65]**
Heterogeneity: Tau² = 0.89; Chi² = 331.33, df = 17 (P < 0.00001); I² = 95%								
Test for overall effect: Z = 4.77 (P < 0.00001)								
Test for subgroup differences: Chi² = 8.10, df = 2 (P = 0.02), I² = 75.3%								

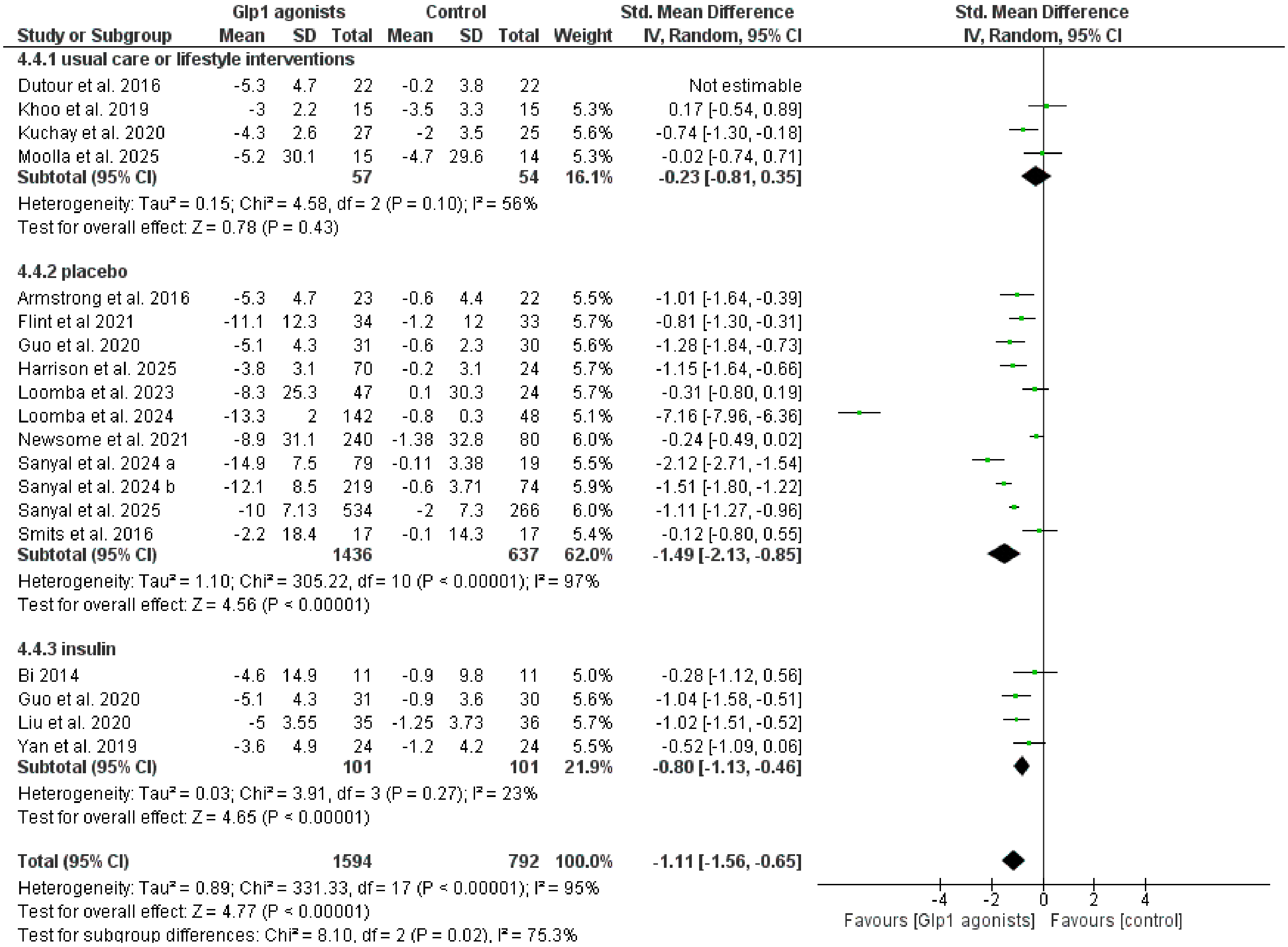

Supplementary Figure S20. Forest plot of change in weight based on the control group after leaving Dutour et al.

Study or Subgroup	Glp1 agonists Mean	SD	Total	Control Mean	SD	Total	Weight	Std. Mean Difference IV, Random, 95% CI
**4.6.1 placebo**								
Armstrong et al. 2016	-0.53	0.64	23	0	0.8	22	6.1%	-0.72 [-1.33, -0.12]
Flint et al 2021	-1.03	0.1	34	-0.03	0.1	33	2.5%	-9.88 [-11.67, -8.09]
Guo et al. 2020	-0.7	1.1	31	-0.1	0.6	30	6.5%	-0.67 [-1.18, -0.15]
Harrison et al. 2025	0.2	1.5	70	0.2	1.7	24	6.7%	0.00 [-0.46, 0.46]
Loomba et al. 2023	-0.98	3.7	47	0.6	1.9	24	6.5%	-0.49 [-0.98, 0.01]
Loomba et al. 2024	-1.4	1.3	125	-0.1	1.2	38	7.0%	-1.01 [-1.39, -0.63]
Newsome et al. 2021	-1	1.2	149	-0.01	1.2	50	7.1%	-0.82 [-1.15, -0.49]
Sanyal et al. 2024 b	-0.7	0.7	219	0.14	0.7	74	7.2%	-1.20 [-1.48, -0.92]
Sanyal et al. 2025	-0.42	0.58	534	0.11	0.58	266	7.5%	-0.91 [-1.07, -0.76]
Smits et al. 2016	-0.7	1.1	17	0.5	1.4	17	5.7%	-0.93 [-1.64, -0.22]
**Subtotal (95% CI)**			**1249**			**578**	**62.8%**	**-1.14 [-1.59, -0.68]**
Heterogeneity: Tau² = 0.45; Chi² = 119.87, df = 9 (P < 0.00001); I² = 92%								
Test for overall effect: Z = 4.91 (P < 0.00001)								
**4.6.2 Insulin**								
Bi 2014	-2.9	1.3	11	-2.8	1.2	11	5.2%	-0.08 [-0.91, 0.76]
Guo et al. 2020	-0.7	1.1	31	-0.5	1	30	6.5%	-0.19 [-0.69, 0.32]
Liu et al. 2020	-2.28	1.01	35	-1.82	1.09	36	6.6%	-0.43 [-0.90, 0.04]
Yan et al. 2019	-1	0.9	24	-0.7	1.3	24	6.3%	-0.26 [-0.83, 0.30]
**Subtotal (95% CI)**			**101**			**101**	**24.7%**	**-0.28 [-0.56, -0.00]**
Heterogeneity: Tau² = 0.00; Chi² = 0.76, df = 3 (P = 0.86); I² = 0%								
Test for overall effect: Z = 1.97 (P = 0.05)								
**4.6.3 usual care or lifestyle intervention**								
Dutour et al. 2016	-0.7	1.4	22	-0.7	1.8	22	6.2%	0.00 [-0.59, 0.59]
Kuchay et al. 2020	-1.6	1	27	-1.3	1	25	6.4%	-0.30 [-0.84, 0.25]
**Subtotal (95% CI)**			**49**			**47**	**12.6%**	**-0.16 [-0.56, 0.24]**
Heterogeneity: Tau² = 0.00; Chi² = 0.52, df = 1 (P = 0.47); I² = 0%								
Test for overall effect: Z = 0.78 (P = 0.44)								
**Total (95% CI)**			**1399**			**726**	**100.0%**	**-0.78 [-1.12, -0.44]**
Heterogeneity: Tau² = 0.40; Chi² = 147.77, df = 15 (P < 0.00001); I² = 90%								
Test for overall effect: Z = 4.48 (P < 0.00001)								
Test for subgroup differences: Chi² = 12.20, df = 2 (P = 0.002), I² = 83.6%								

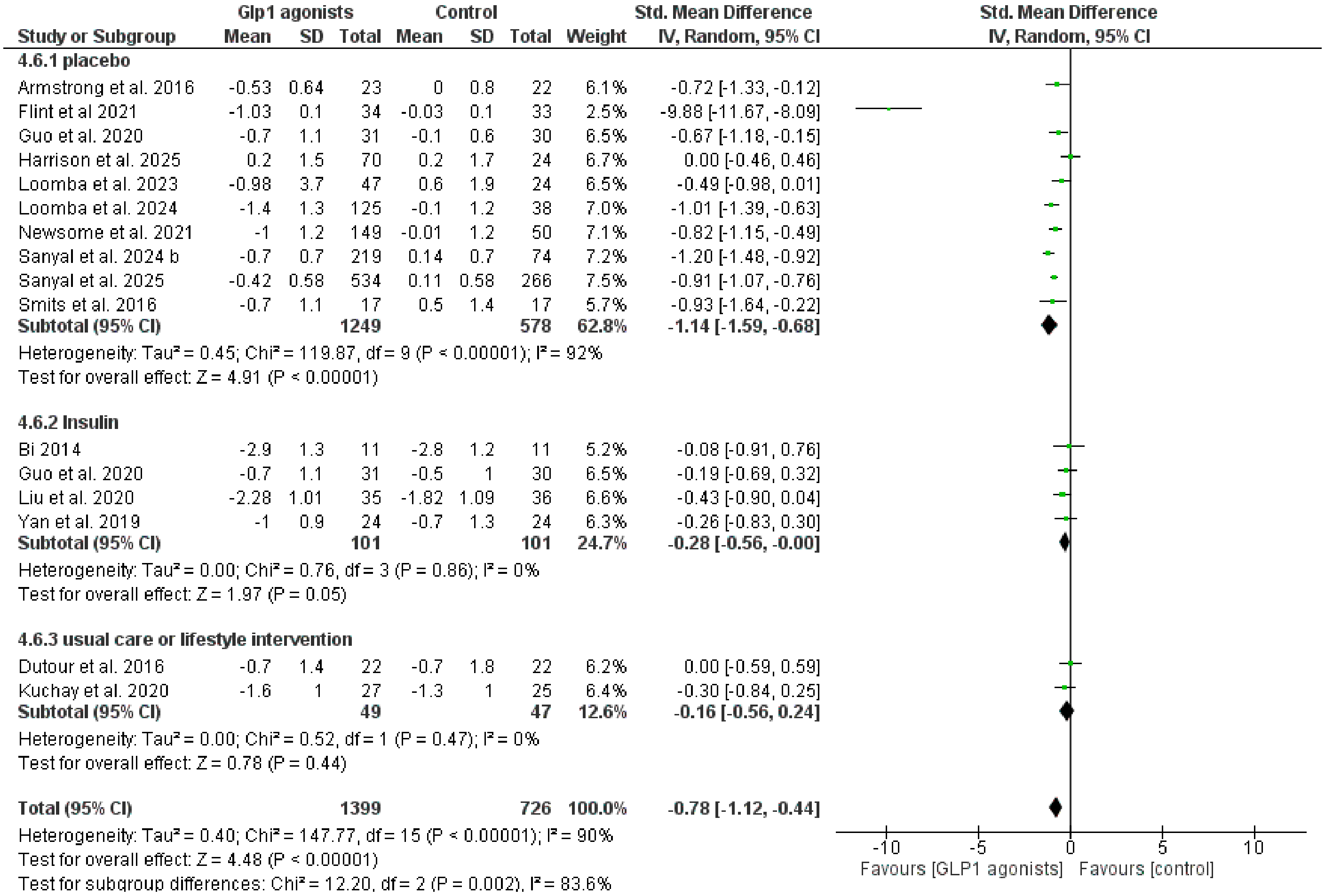

Supplementary Figure S21. Forest plot of change in HbA1c based on the control group.

Study or Subgroup	Glp1 agonists Mean	SD	Total	Control Mean	SD	Total	Weight	Std. Mean Difference IV, Random, 95% CI
**3.4.1 Single agonists**								
Armstrong et al. 2016	-5.3	4.7	23	-0.6	4.4	22	5.5%	-1.01 [-1.64, -0.39]
Bi 2014	-4.6	14.9	11	-0.9	9.8	11	5.0%	-0.28 [-1.12, 0.56]
Dutour et al. 2016	-5.3	4.7	22	-0.2	3.8	22	5.4%	-1.17 [-1.82, -0.53]
Flint et al 2021	-11.1	12.3	34	-1.2	12	33	5.7%	-0.81 [-1.30, -0.31]
Guo et al. 2020	-5.1	4.3	31	-0.75	3	60	5.7%	-1.23 [-1.71, -0.76]
Khoo et al. 2019	-3	2.2	15	-3.5	3.3	15	5.3%	0.17 [-0.54, 0.89]
Kuchay et al. 2020	-4.3	2.6	27	-2	3.5	25	5.6%	-0.74 [-1.30, -0.18]
Liu et al. 2020	-5	3.55	35	-1.25	3.73	36	5.7%	-1.02 [-1.51, -0.52]
Loomba et al. 2023	-8.3	25.3	47	0.1	30.3	24	5.7%	-0.31 [-0.80, 0.19]
Moolla et al. 2025	-5.2	30.1	15	-4.7	29.6	14	5.3%	-0.02 [-0.74, 0.71]
Newsome et al. 2021	-8.9	31.1	240	-1.38	32.8	80	6.0%	-0.24 [-0.49, 0.02]
Sanyal et al. 2025	-10	7.13	534	-2	7.3	266	6.0%	-1.11 [-1.27, -0.96]
Smits et al. 2016	-2.2	18.4	17	-0.1	14.3	17	5.4%	-0.12 [-0.80, 0.55]
Yan et al. 2019	-3.6	4.9	24	-1.2	4.2	24	5.5%	-0.52 [-1.09, 0.06]
**Subtotal (95% CI)**			**1075**			**649**	**77.7%**	**-0.63 [-0.90, -0.37]**
Heterogeneity: Tau² = 0.18; Chi² = 61.44, df = 13 (P < 0.00001); I² = 79%								
Test for overall effect: Z = 4.64 (P < 0.00001)								
**3.4.2 Dual or triple agonists**								
Harrison et al. 2025	-3.8	3.1	70	-0.2	3.1	24	5.7%	-1.15 [-1.64, -0.66]
Loomba et al. 2024	-13.3	2	142	-0.8	0.3	48	5.1%	-7.16 [-7.96, -6.36]
Sanyal et al. 2024 a	-14.9	7.5	79	-0.11	3.38	19	5.5%	-2.12 [-2.71, -1.54]
Sanyal et al. 2024 b	-12.1	8.5	219	-0.6	3.71	74	5.9%	-1.51 [-1.80, -1.22]
**Subtotal (95% CI)**			**510**			**165**	**22.3%**	**-2.96 [-4.90, -1.02]**
Heterogeneity: Tau² = 3.84; Chi² = 183.01, df = 3 (P < 0.00001); I² = 98%								
Test for overall effect: Z = 2.99 (P = 0.003)								
**Total (95% CI)**			**1585**			**814**	**100.0%**	**-1.11 [-1.57, -0.66]**
Heterogeneity: Tau² = 0.89; Chi² = 331.52, df = 17 (P < 0.00001); I² = 95%								
Test for overall effect: Z = 4.80 (P < 0.00001)								
Test for subgroup differences: Chi² = 5.40, df = 1 (P = 0.02), I² = 81.5%								

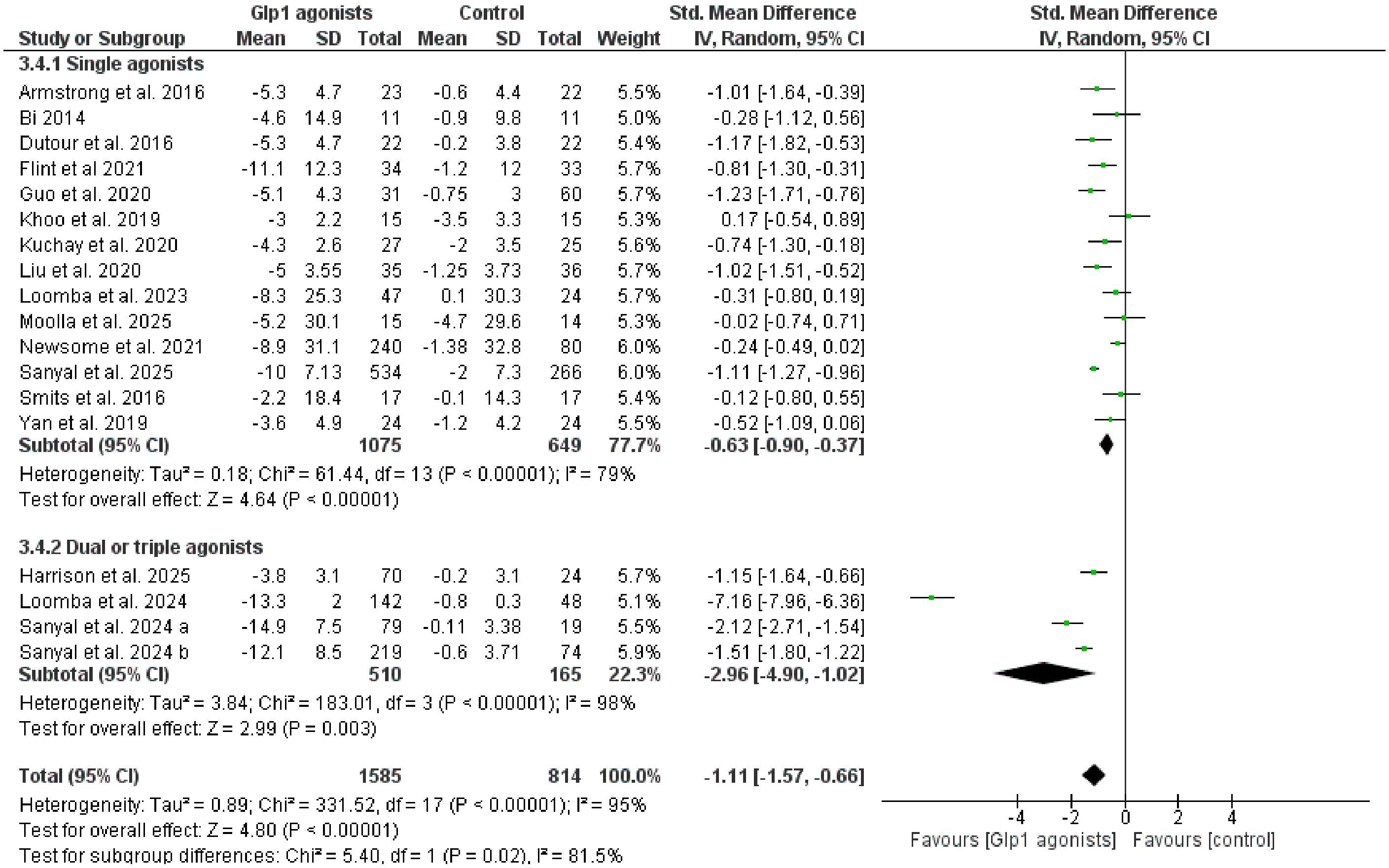

Supplementary Figure S22. Forest plot of change in weight based on the intervention group (single, or dual or triple agonists).

Study or Subgroup	Glp1 agonists Mean	SD	Total	Control Mean	SD	Total	Weight	Std. Mean Difference IV, Random, 95% CI
**3.5.1 Single agonists**								
Armstrong et al. 2016	-0.53	0.64	23	0	0.8	22	6.6%	-0.72 [-1.33, -0.12]
Bi 2014	-2.9	1.3	11	-2.8	1.2	11	5.6%	-0.08 [-0.91, 0.76]
Dutour et al. 2016	-0.7	1.4	22	-0.7	1.8	22	6.6%	0.00 [-0.59, 0.59]
Flint et al 2021	-1.03	0.1	34	-0.03	0.1	33	2.6%	-9.88 [-11.67, -8.09]
Guo et al. 2020	-0.7	1.1	31	-0.3	0.8	60	7.2%	-0.43 [-0.87, 0.00]
Kuchay et al. 2020	-1.6	1	27	-1.3	1	25	6.8%	-0.30 [-0.84, 0.25]
Liu et al. 2020	-2.28	1.01	35	-1.82	1.09	36	7.1%	-0.43 [-0.90, 0.04]
Loomba et al. 2023	-0.98	3.7	47	0.6	1.9	24	7.0%	-0.49 [-0.98, 0.01]
Newsome et al. 2021	-1	1.2	149	-0.01	1.2	50	7.6%	-0.82 [-1.15, -0.49]
Sanyal et al. 2025	-0.42	0.58	534	0.11	0.58	266	8.0%	-0.91 [-1.07, -0.76]
Smits et al. 2016	-0.7	1.1	17	0.5	1.4	17	6.1%	-0.93 [-1.64, -0.22]
Yan et al. 2019	-1	0.9	24	-0.7	1.3	24	6.7%	-0.26 [-0.83, 0.30]
**Subtotal (95% CI)**			**954**			**590**	**77.8%**	**-0.86 [-1.31, -0.41]**
Heterogeneity: Tau² = 0.52; Chi² = 123.68, df = 11 (P < 0.00001); I² = 91%								
Test for overall effect: Z = 3.77 (P = 0.0002)								
**3.5.2 Dual agonists**								
Harrison et al. 2025	0.2	1.5	70	0.2	1.7	24	7.1%	0.00 [-0.46, 0.46]
Loomba et al. 2024	-1.4	1.3	125	-0.1	1.2	38	7.4%	-1.01 [-1.39, -0.63]
Sanyal et al. 2024 b	-0.7	0.7	219	0.14	0.7	74	7.7%	-1.20 [-1.48, -0.92]
**Subtotal (95% CI)**			**414**			**136**	**22.2%**	**-0.76 [-1.42, -0.10]**
Heterogeneity: Tau² = 0.30; Chi² = 19.08, df = 2 (P < 0.0001); I² = 90%								
Test for overall effect: Z = 2.25 (P = 0.02)								
**Total (95% CI)**			**1368**			**726**	**100.0%**	**-0.81 [-1.16, -0.45]**
Heterogeneity: Tau² = 0.40; Chi² = 144.59, df = 14 (P < 0.00001); I² = 90%								
Test for overall effect: Z = 4.45 (P < 0.00001)								
Test for subgroup differences: Chi² = 0.06, df = 1 (P = 0.80), I² = 0%								

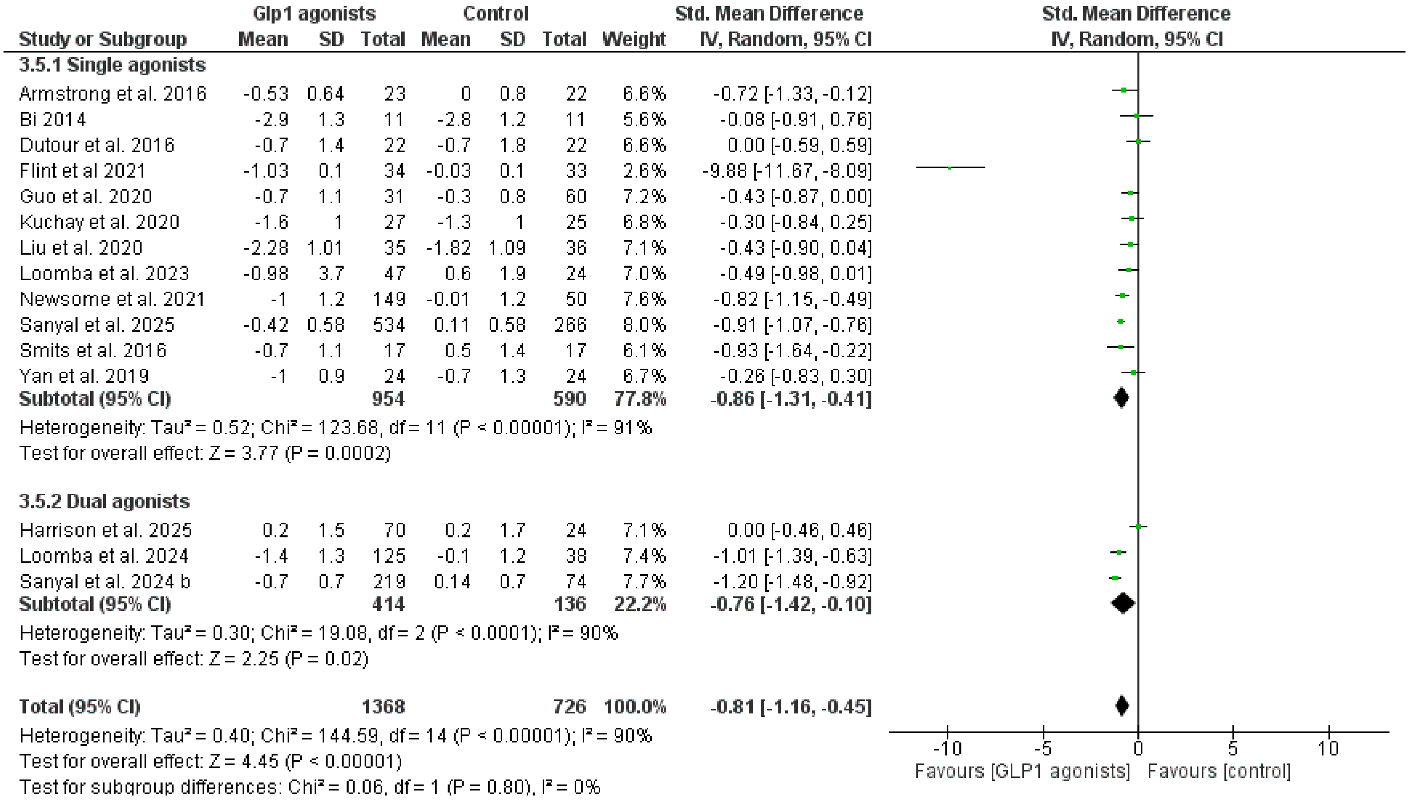

Supplementary Figure S23. Forest plot of change in HbA1c based on the intervention group (single, or dual or triple agonists).

Study or Subgroup	Glp1 agonists Mean	SD	Total	Control Mean	SD	Total	Weight	Std. Mean Difference IV, Random, 95% CI
**3.5.1 Single agonists**								
Armstrong et al. 2016	-0.53	0.64	23	0	0.8	22	7.0%	-0.72 [-1.33, -0.12]
Bi 2014	-2.9	1.3	11	-2.8	1.2	11	6.0%	-0.08 [-0.91, 0.76]
Dutour et al. 2016	-0.7	1.4	22	-0.7	1.8	22	7.1%	0.00 [-0.59, 0.59]
Flint et al 2021	-1.03	0.1	34	-0.03	0.1	33	2.8%	-9.88 [-11.67, -8.09]
Guo et al. 2020	-0.7	1.1	31	-0.3	0.8	60	7.8%	-0.43 [-0.87, 0.00]
Kuchay et al. 2020	-1.6	1	27	-1.3	1	25	7.3%	-0.30 [-0.84, 0.25]
Liu et al. 2020	-2.28	1.01	35	-1.82	1.09	36	7.6%	-0.43 [-0.90, 0.04]
Loomba et al. 2023	-0.98	3.7	47	0.6	1.9	24	7.5%	-0.49 [-0.98, 0.01]
Newsome et al. 2021	-1	1.2	149	-0.01	1.2	50	8.2%	-0.82 [-1.15, -0.49]
Sanyal et al. 2025	-0.42	0.58	534	0.11	0.58	266	8.6%	-0.91 [-1.07, -0.76]
Smits et al. 2016	-0.7	1.1	17	0.5	1.4	17	6.6%	-0.93 [-1.64, -0.22]
Yan et al. 2019	-1	0.9	24	-0.7	1.3	24	7.2%	-0.26 [-0.83, 0.30]
**Subtotal (95% CI)**			**954**			**590**	**83.7%**	**-0.86 [-1.31, -0.41]**
Heterogeneity: Tau^2 = 0.52; Chi^2 = 123.68, df = 11 ($P < 0.00001$); I^2 = 91%								
Test for overall effect: Z = 3.77 (P = 0.0002)								
**3.5.2 Dual agonists**								
Harrison et al. 2025	0.2	1.5	70	0.2	1.7	24	0.0%	0.00 [-0.46, 0.46]
Loomba et al. 2024	-1.4	1.3	125	-0.1	1.2	38	8.0%	-1.01 [-1.39, -0.63]
Sanyal et al. 2024 b	-0.7	0.7	219	0.14	0.7	74	8.3%	-1.20 [-1.48, -0.92]
**Subtotal (95% CI)**			**344**			**112**	**16.3%**	**-1.13 [-1.36, -0.91]**
Heterogeneity: Tau^2 = 0.00; Chi^2 = 0.58, df = 1 (P = 0.44); I^2 = 0%								
Test for overall effect: Z = 9.82 ($P < 0.00001$)								
**Total (95% CI)**			**1298**			**702**	**100.0%**	**-0.86 [-1.23, -0.50]**
Heterogeneity: Tau^2 = 0.40; Chi^2 = 132.86, df = 13 ($P < 0.00001$); I^2 = 90%								
Test for overall effect: Z = 4.65 ($P < 0.00001$)								
Test for subgroup differences: Chi^2 = 1.13, df = 1 (P = 0.29), I^2 = 11.8%								

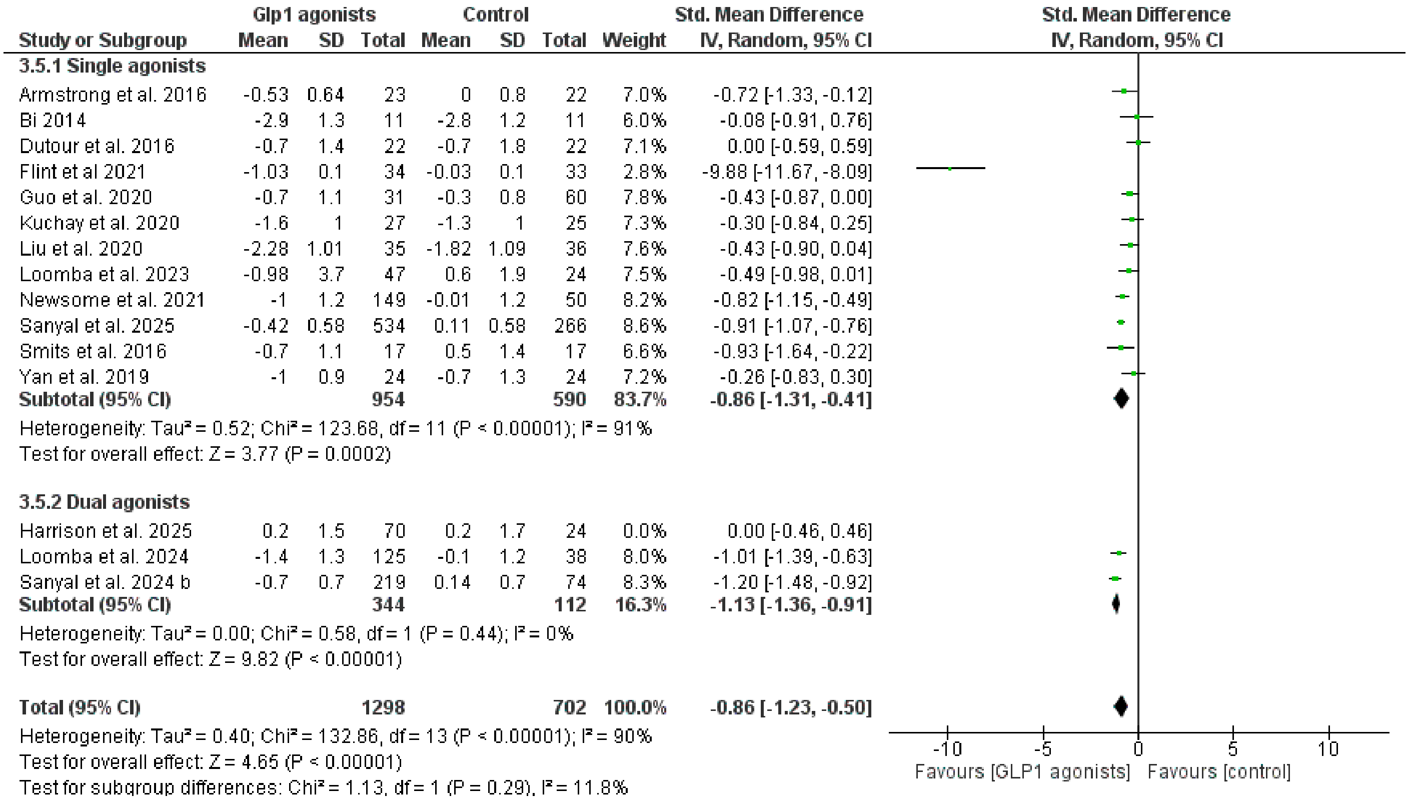

Supplementary Figure S24. Forest plot of change in HbA1c based on the intervention group (single, or dual or triple agonists) after leaving Harrison et al.

Study or Subgroup	Glp1 agonists Mean	SD	Total	Control Mean	SD	Total	Weight	Std. Mean Difference IV, Random, 95% CI
Dutour et al. 2016	-4.39	12.34	22	0.47	15.02	22	20.0%	-0.35 [-0.94, 0.25]
Harrison et al. 2025	4.8	8	70	5.8	7.5	24	25.4%	-0.13 [-0.59, 0.34]
Khoo et al. 2019	-1.39	1.2	15	-0.05	0.76	15	14.0%	-1.30 [-2.10, -0.50]
Sanyal et al. 2025	-53.83	53.6	534	-19.83	53.6	266	40.5%	-0.63 [-0.78, -0.48]
**Total (95% CI)**			**641**			**327**	**100.0%**	**-0.54 [-0.90, -0.18]**
Heterogeneity: Tau^2 = 0.08; Chi^2 = 7.83, df = 3 (P = 0.05); I^2 = 62%								
Test for overall effect: Z = 2.92 (P = 0.004)								

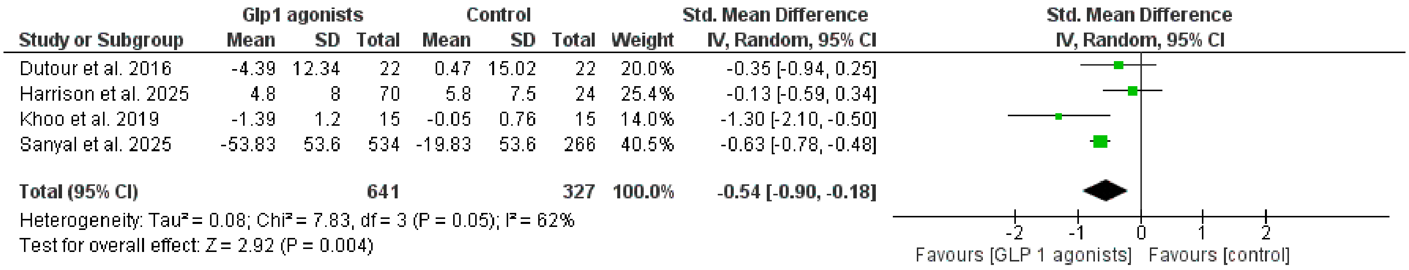

Supplementary Figure S25. Forest plot of change in CRP in the overall population .

Study or Subgroup	Glp1 agonists Mean	SD	Total	Control Mean	SD	Total	Weight	Std. Mean Difference IV, Random, 95% CI
Dutour et al. 2016	-4.39	12.34	22	0.47	15.02	22	23.7%	-0.35 [-0.94, 0.25]
Harrison et al. 2025	4.8	8	70	5.8	7.5	24	0.0%	-0.13 [-0.59, 0.34]
Khoo et al. 2019	-1.39	1.2	15	-0.05	0.76	15	15.6%	-1.30 [-2.10, -0.50]
Sanyal et al. 2025	-53.83	53.6	534	-19.83	53.6	266	60.7%	-0.63 [-0.78, -0.48]
**Total (95% CI)**			**571**			**303**	**100.0%**	**-0.67 [-1.03, -0.31]**
Heterogeneity: Tau^2 = 0.05; Chi^2 = 3.55, df = 2 (P = 0.17); I^2 = 44%								
Test for overall effect: Z = 3.66 (P = 0.0003)								

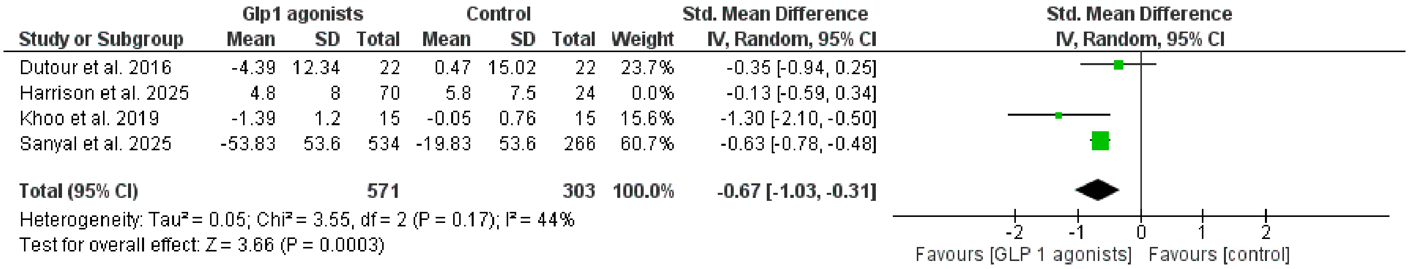

Supplementary Figure S26. Forest plot of the change in CRP in the overall population after leaving Harrison et al.

Study or Subgroup	Glp1 agonists Mean	SD	Total	Control Mean	SD	Total	Weight	Std. Mean Difference IV, Random, 95% CI
Armstrong et al. 2016	-15.8	21.8	23	-8.6	28.3	22	6.1%	-0.28 [-0.87, 0.31]
Bi 2014	3	14.4	11	1.7	9	11	5.2%	0.10 [-0.73, 0.94]
Dutour et al. 2016	5	51.5	22	-2	16.4	22	6.0%	0.18 [-0.41, 0.77]
Guo et al. 2020	-5.3	11.8	31	-2	10.75	60	6.6%	-0.29 [-0.73, 0.14]
Khoo et al. 2019	-14	15	15	-22	23	15	5.6%	0.40 [-0.32, 1.12]
Kuchay et al. 2020	-16.6	17.7	27	-7.3	19	25	6.2%	-0.50 [-1.05, 0.05]
Liu et al. 2020	-12.33	13.15	35	-2.66	14.26	36	6.4%	-0.70 [-1.18, -0.22]
Loomba et al. 2023	-16	28.7	47	1.5	33.2	24	6.4%	-0.57 [-1.07, -0.07]
Loomba et al. 2024	-22.7	13.9	142	-2	27.9	48	6.8%	-1.12 [-1.47, -0.77]
Moolla et al. 2025	-5	19.4	15	-5	24	14	5.6%	0.00 [-0.73, 0.73]
Newsome et al. 2021	-17	4.5	220	-6.53	3.15	74	6.9%	-2.48 [-2.82, -2.15]
Sanyal et al. 2024 a	-17.6	26	79	-7.2	13.9	19	6.3%	-0.43 [-0.93, 0.08]
Sanyal et al. 2024 b	-29.2	36.3	158	-2.4	24.9	63	6.9%	-0.80 [-1.10, -0.50]
Sanyal et al. 2025	-26	30.4	534	-9.4	37.12	266	7.2%	-0.51 [-0.66, -0.36]
Smits et al. 2016	-1.8	9.7	17	-0.6	10.2	17	5.8%	-0.12 [-0.79, 0.56]
Yan et al. 2019	-1.8	9.3	24	-2.9	23.4	24	6.1%	0.06 [-0.51, 0.63]
**Total (95% CI)**			**1400**			**740**	**100.0%**	**-0.48 [-0.83, -0.13]**

Heterogeneity: $Tau^2 = 0.44$; $Chi^2 = 164.29$, df = 15 (P < 0.00001); $I^2 = 91\%$
Test for overall effect: Z = 2.67 (P = 0.008)

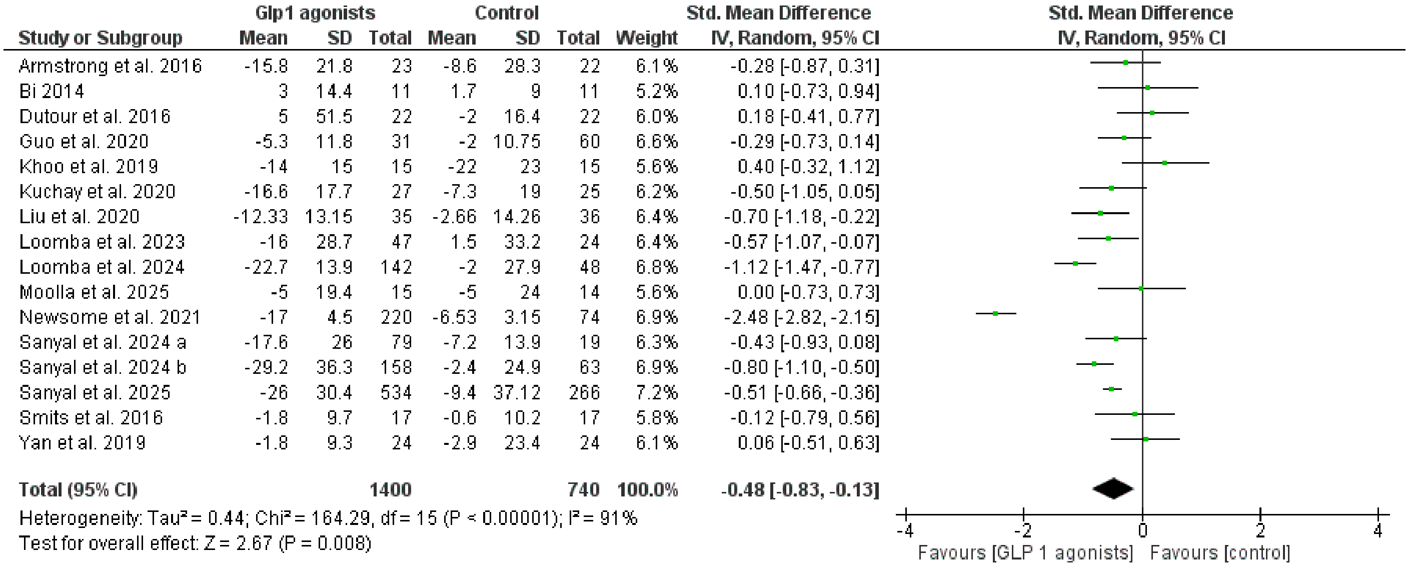

Supplementary Figure S27. Forest plot of the change in AST in the overall population.

Study or Subgroup	Glp1 agonists Mean	SD	Total	Control Mean	SD	Total	Weight	Std. Mean Difference IV, Random, 95% CI
**4.9.1 placebo**								
Armstrong et al. 2016	-15.8	21.8	23	-8.6	28.3	22	5.7%	-0.28 [-0.87, 0.31]
Guo et al. 2020	-5.3	11.8	31	-1.8	11	30	6.0%	-0.30 [-0.81, 0.20]
Loomba et al. 2023	-16	28.7	47	1.5	33.2	24	6.0%	-0.57 [-1.07, -0.07]
Loomba et al. 2024	-22.7	13.9	142	-2	27.9	48	6.4%	-1.12 [-1.47, -0.77]
Newsome et al. 2021	-17	4.5	220	-6.53	3.15	74	6.5%	-2.48 [-2.82, -2.15]
Sanyal et al. 2024 a	-17.6	26	79	-7.2	13.9	19	6.0%	-0.43 [-0.93, 0.08]
Sanyal et al. 2024 b	-29.2	36.3	158	-2.4	24.9	63	6.5%	-0.80 [-1.10, -0.50]
Sanyal et al. 2025	-26	30.4	534	-9.4	37.12	266	6.8%	-0.51 [-0.66, -0.36]
Smits et al. 2016	-1.8	9.7	17	-0.6	10.2	17	5.4%	-0.12 [-0.79, 0.56]
**Subtotal (95% CI)**			**1251**			**563**	**55.4%**	**-0.76 [-1.24, -0.27]**
Heterogeneity: $Tau^2 = 0.50$; $Chi^2 = 130.34$, df = 8 (P < 0.00001); $I^2 = 94\%$								
Test for overall effect: Z = 3.06 (P = 0.002)								
**4.9.2 insulin**								
Bi 2014	3	14.4	11	1.7	9	11	4.8%	0.10 [-0.73, 0.94]
Guo et al. 2020	-5.3	11.8	31	-2.3	10.5	30	6.0%	-0.26 [-0.77, 0.24]
Liu et al. 2020	-12.33	13.15	35	-2.66	14.26	36	6.1%	-0.70 [-1.18, -0.22]
Yan et al. 2019	-1.8	9.3	24	-2.9	23.4	24	5.8%	0.06 [-0.51, 0.63]
**Subtotal (95% CI)**			**101**			**101**	**22.7%**	**-0.26 [-0.63, 0.12]**
Heterogeneity: $Tau^2 = 0.06$; $Chi^2 = 5.10$, df = 3 (P = 0.16); $I^2 = 41\%$								
Test for overall effect: Z = 1.35 (P = 0.18)								
**4.9.3 usual care or lifestyle interventions**								
Dutour et al. 2016	5	51.5	22	-2	16.4	22	5.7%	0.18 [-0.41, 0.77]
Khoo et al. 2019	-14	15	15	-22	23	15	5.2%	0.40 [-0.32, 1.12]
Kuchay et al. 2020	-16.6	17.7	27	-7.3	19	25	5.8%	-0.50 [-1.05, 0.05]
Moolla et al. 2025	-5	19.4	15	-5	24	14	5.2%	0.00 [-0.73, 0.73]
**Subtotal (95% CI)**			**79**			**76**	**22.0%**	**-0.02 [-0.41, 0.38]**
Heterogeneity: $Tau^2 = 0.06$; $Chi^2 = 4.62$, df = 3 (P = 0.20); $I^2 = 35\%$								
Test for overall effect: Z = 0.07 (P = 0.94)								
**Total (95% CI)**			**1431**			**740**	**100.0%**	**-0.47 [-0.80, -0.13]**

Heterogeneity: $Tau^2 = 0.43$; $Chi^2 = 165.83$, df = 16 (P < 0.00001); $I^2 = 90\%$
Test for overall effect: Z = 2.72 (P = 0.006)
Test for subgroup differences: $Chi^2 = 5.42$, df = 2 (P = 0.07), $I^2 = 63.1\%$

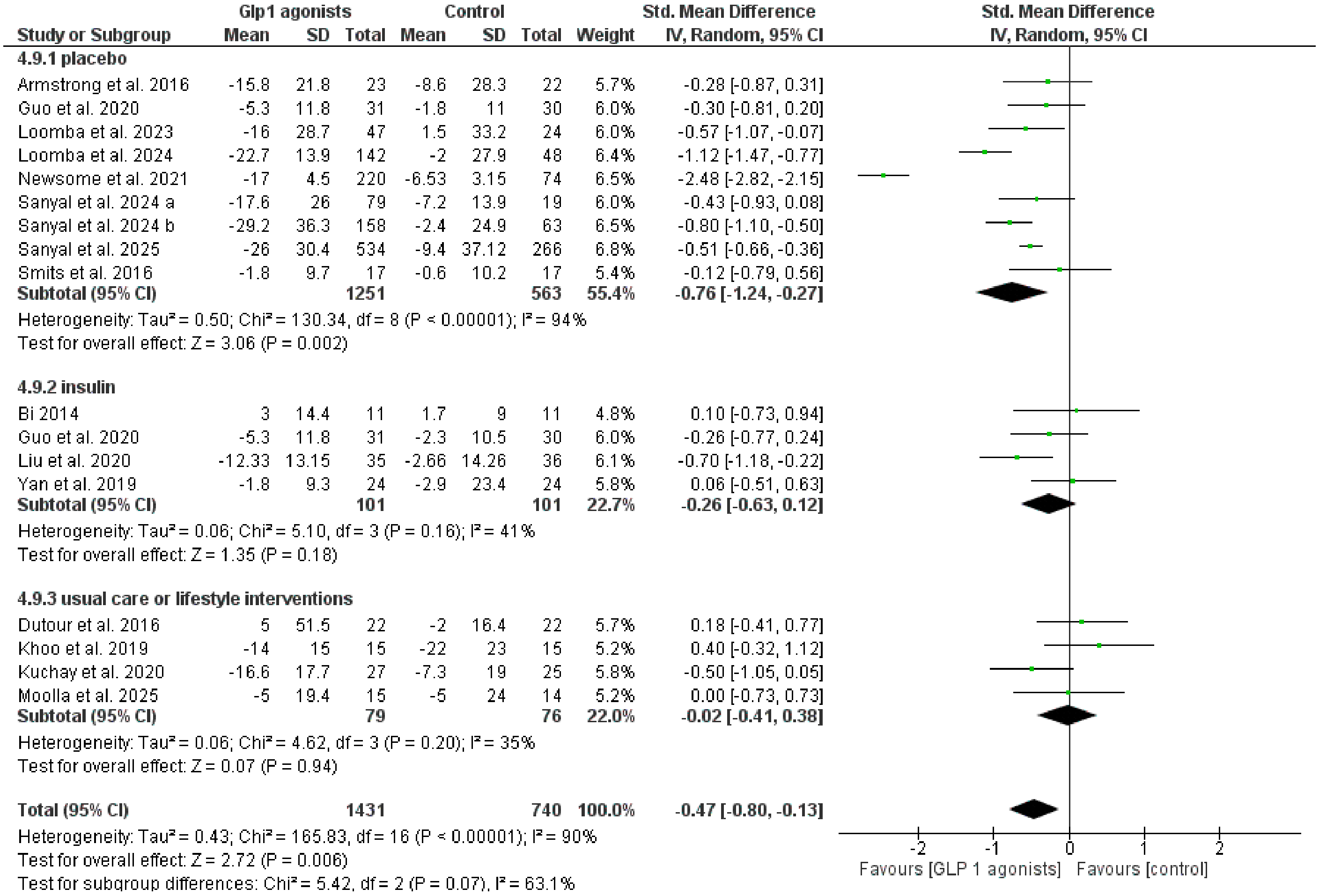

Supplementary Figure S28. Forest plot of the change in AST based on the control group.

Study or Subgroup	Glp1 agonists Mean	SD	Total	Control Mean	SD	Total	Weight	Std. Mean Difference IV, Fixed, 95% CI
**3.9.1 Single agonists**								
Armstrong et al. 2016	-15.8	21.8	23	-8.6	28.3	22	2.6%	-0.28 [-0.87, 0.31]
Bi 2014	3	14.4	11	1.7	9	11	1.3%	0.10 [-0.73, 0.94]
Dutour et al. 2016	5	51.5	22	-2	16.4	22	2.6%	0.18 [-0.41, 0.77]
Guo et al. 2020	-5.3	11.8	31	2	10.75	60	4.6%	-0.65 [-1.10, -0.21]
Khoo et al. 2019	-14	15	15	-22	23	15	1.7%	0.40 [-0.32, 1.12]
Kuchay et al. 2020	-16.6	17.7	27	-7.3	19	25	3.0%	-0.50 [-1.05, 0.05]
Liu et al. 2020	-12.33	13.15	35	-2.66	14.26	36	3.9%	-0.70 [-1.18, -0.22]
Loomba et al. 2023	-16	28.7	47	1.5	33.2	24	3.6%	-0.57 [-1.07, -0.07]
Moolla et al. 2025	-5	19.4	15	-5	24	14	1.7%	0.00 [-0.73, 0.73]
Newsome et al. 2021	-17	4.5	220	-6.53	3.15	74	8.2%	-2.48 [-2.82, -2.15]
Sanyal et al. 2025	-26	30.4	534	-9.4	37.12	266	40.8%	-0.51 [-0.66, -0.36]
Smits et al. 2016	-1.8	9.7	17	-0.6	10.2	17	2.0%	-0.12 [-0.79, 0.56]
Yan et al. 2019	-1.8	9.3	24	-2.9	23.4	24	2.8%	0.06 [-0.51, 0.63]
**Subtotal (95% CI)**			**1021**			**610**	**78.9%**	**-0.63 [-0.74, -0.52]**
Heterogeneity: Chi² = 152.95, df = 12 (P < 0.00001); I² = 92%								
Test for overall effect: Z = 11.55 (P < 0.00001)								
**3.9.2 Dual or triple agonists**								
Loomba et al. 2024	-22.7	13.9	142	-2	27.9	48	7.6%	-1.12 [-1.47, -0.77]
Sanyal et al. 2024 a	-17.6	26	79	-7.2	13.9	19	3.6%	-0.43 [-0.93, 0.08]
Sanyal et al. 2024 b	-29.2	36.3	158	-2.4	24.9	63	10.0%	-0.80 [-1.10, -0.50]
**Subtotal (95% CI)**			**379**			**130**	**21.1%**	**-0.85 [-1.06, -0.64]**
Heterogeneity: Chi² = 5.14, df = 2 (P = 0.08); I² = 61%								
Test for overall effect: Z = 8.04 (P < 0.00001)								
**Total (95% CI)**			**1400**			**740**	**100.0%**	**-0.68 [-0.77, -0.58]**
Heterogeneity: Chi² = 161.45, df = 15 (P < 0.00001); I² = 91%								
Test for overall effect: Z = 13.95 (P < 0.00001)								
Test for subgroup differences: Chi² = 3.36, df = 1 (P = 0.07), I² = 70.2%								

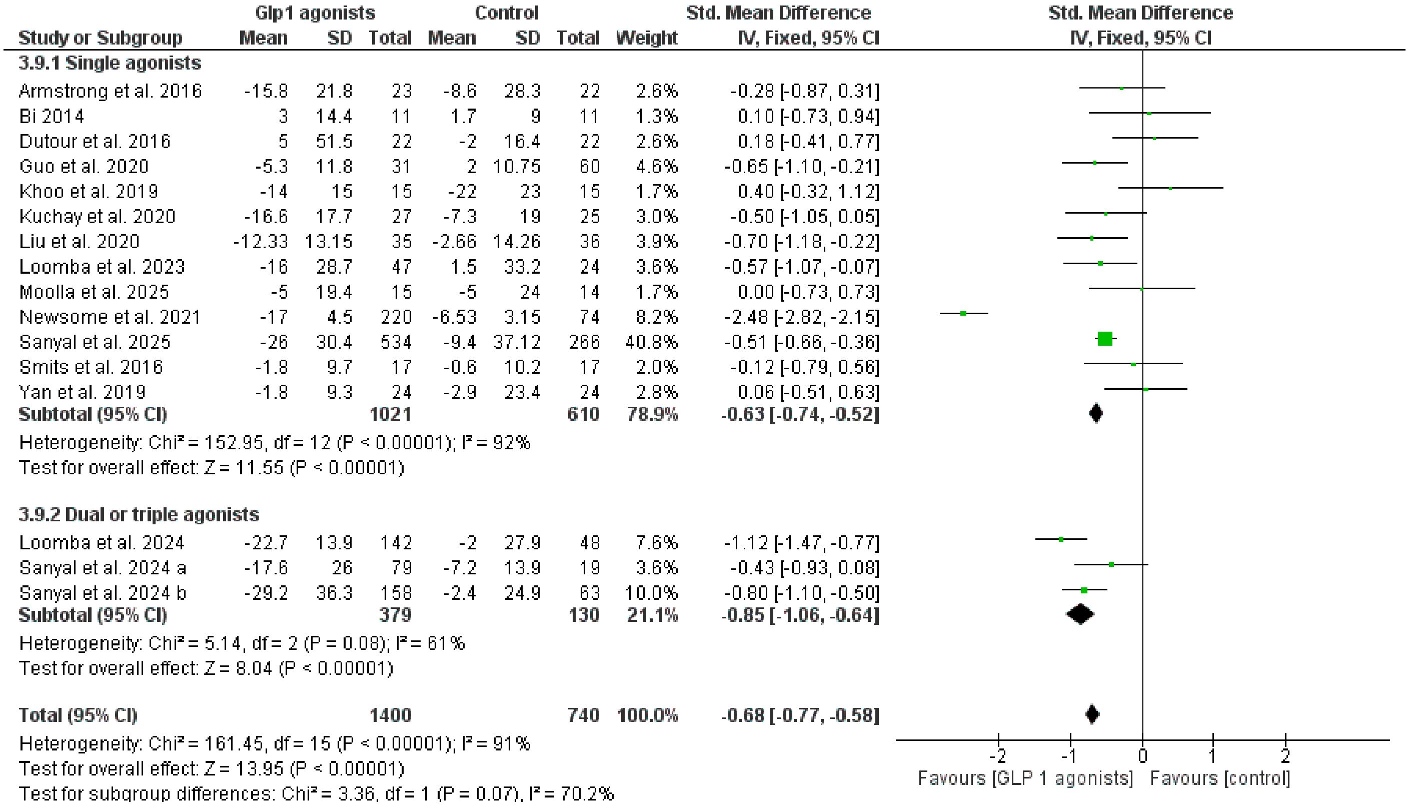

Supplementary Figure S29. Forest plot of the change in AST based on the intervention group (single, or dual or triple agonists).

Study or Subgroup	Glp1 agonists Mean	SD	Total	Control Mean	SD	Total	Weight	Std. Mean Difference IV, Fixed, 95% CI
**3.9.1 Single agonists**								
Armstrong et al. 2016	-15.8	21.8	23	-8.6	28.3	22	2.8%	-0.28 [-0.87, 0.31]
Bi 2014	3	14.4	11	1.7	9	11	1.4%	0.10 [-0.73, 0.94]
Dutour et al. 2016	5	51.5	22	-2	16.4	22	2.8%	0.18 [-0.41, 0.77]
Guo et al. 2020	-5.3	11.8	31	2	10.75	60	5.0%	-0.65 [-1.10, -0.21]
Khoo et al. 2019	-14	15	15	-22	23	15	1.9%	0.40 [-0.32, 1.12]
Kuchay et al. 2020	-16.6	17.7	27	-7.3	19	25	3.2%	-0.50 [-1.05, 0.05]
Liu et al. 2020	-12.33	13.15	35	-2.66	14.26	36	4.3%	-0.70 [-1.18, -0.22]
Loomba et al. 2023	-16	28.7	47	1.5	33.2	24	3.9%	-0.57 [-1.07, -0.07]
Moolla et al. 2025	-5	19.4	15	-5	24	14	1.8%	0.00 [-0.73, 0.73]
Newsome et al. 2021	-17	4.5	220	-6.53	3.15	74	8.9%	-2.48 [-2.82, -2.15]
Sanyal et al. 2025	-26	30.4	534	-9.4	37.12	266	44.1%	-0.51 [-0.66, -0.36]
Smits et al. 2016	-1.8	9.7	17	-0.6	10.2	17	2.2%	-0.12 [-0.79, 0.56]
Yan et al. 2019	-1.8	9.3	24	-2.9	23.4	24	3.1%	0.06 [-0.51, 0.63]
**Subtotal (95% CI)**			**1021**			**610**	**85.4%**	**-0.63 [-0.74, -0.52]**
Heterogeneity: Chi² = 152.95, df = 12 (P < 0.00001); I² = 92%								
Test for overall effect: Z = 11.55 (P < 0.00001)								
**3.9.2 Dual or triple agonists**								
Loomba et al. 2024	-22.7	13.9	142	-2	27.9	48	0.0%	-1.12 [-1.47, -0.77]
Sanyal et al. 2024 a	-17.6	26	79	-7.2	13.9	19	3.9%	-0.43 [-0.93, 0.08]
Sanyal et al. 2024 b	-29.2	36.3	158	-2.4	24.9	63	10.8%	-0.80 [-1.10, -0.50]
**Subtotal (95% CI)**			**237**			**82**	**14.6%**	**-0.70 [-0.96, -0.44]**
Heterogeneity: Chi² = 1.54, df = 1 (P = 0.22); I² = 35%								
Test for overall effect: Z = 5.30 (P < 0.00001)								
**Total (95% CI)**			**1258**			**692**	**100.0%**	**-0.64 [-0.74, -0.54]**
Heterogeneity: Chi² = 154.71, df = 14 (P < 0.00001); I² = 91%								
Test for overall effect: Z = 12.70 (P < 0.00001)								
Test for subgroup differences: Chi² = 0.23, df = 1 (P = 0.63), I² = 0%								

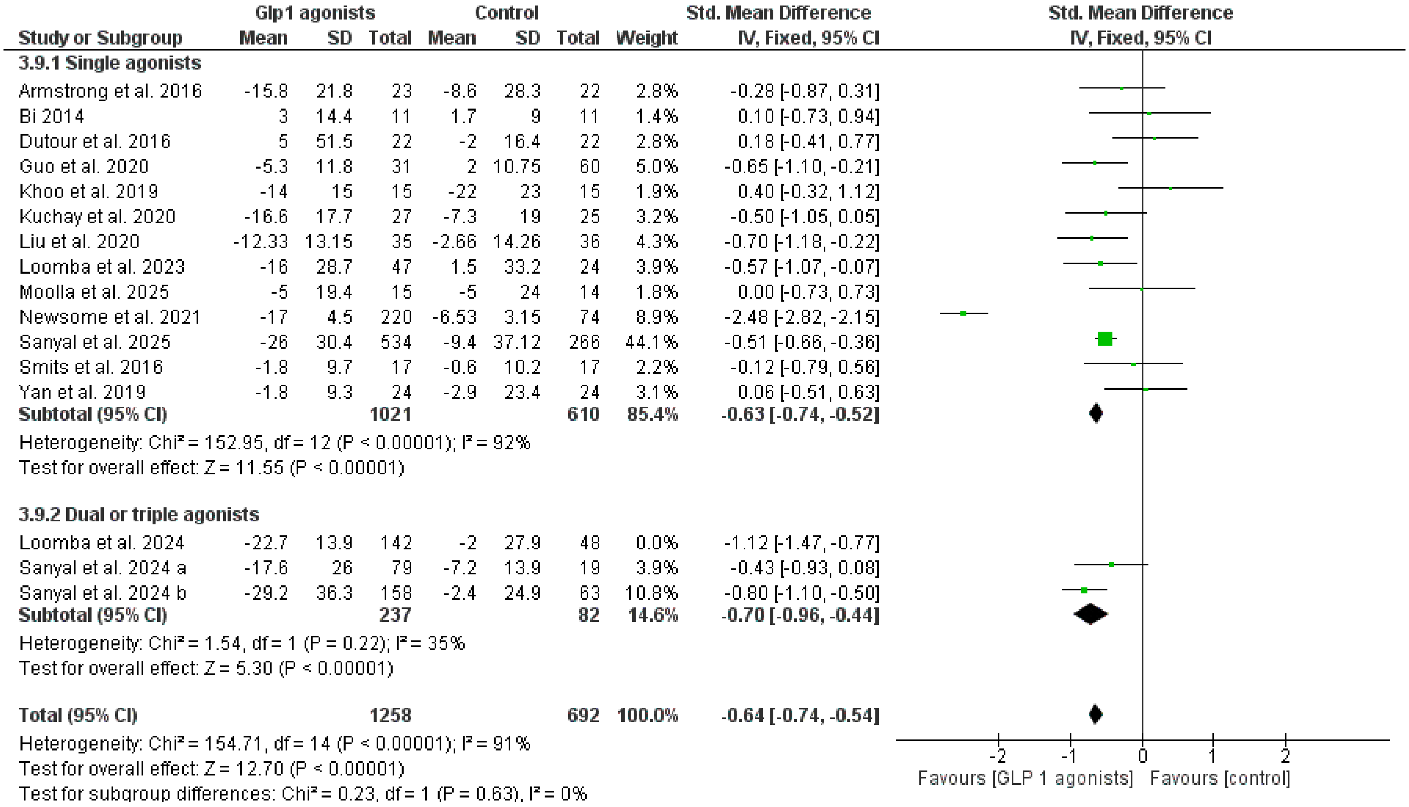

Supplementary Figure S30. Forest plot of the change in AST based on the intervention group (single, or dual or triple agonists) after leaving Loomba et al.

	GLP 1 agonists			Control				Std. Mean Difference
Study or Subgroup	Mean	SD	Total	Mean	SD	Total	Weight	IV, Fixed, 95% CI
**2.5.1 patients with type 2 DM**								
Bi 2014	3	14.4	11	1.7	9	11	5.3%	0.10 [-0.73, 0.94]
Dutour et al. 2016	5	51.5	22	-2	16.4	22	10.6%	0.18 [-0.41, 0.77]
Guo et al. 2020	-5.3	11.8	31	-2.3	10.5	30	14.6%	-0.26 [-0.77, 0.24]
Kuchay et al. 2020	-16.6	17.7	27	-7.3	19	25	12.1%	-0.50 [-1.05, 0.05]
Liu et al. 2020	-12.33	13.15	35	-2.66	14.26	36	16.1%	-0.70 [-1.18, -0.22]
Smits et al. 2016	-1.8	9.7	17	-0.6	10.2	17	8.2%	-0.12 [-0.79, 0.56]
Yan et al. 2019	-1.8	9.3	24	-2.9	23.4	24	11.6%	0.06 [-0.51, 0.63]
**Subtotal (95% CI)**			**167**			**165**	**78.4%**	**-0.24 [-0.46, -0.02]**
Heterogeneity: Chi² = 8.13, df = 6 (P = 0.23); I² = 26%								
Test for overall effect: Z = 2.18 (P = 0.03)								
**2.5.2 patients without type 2 diabetes**								
Moolla et al. 2025	-5	19.4	15	-5	24	14	7.0%	0.00 [-0.73, 0.73]
Sanyal et al. 2024 a	-17.6	26	79	-7.2	13.9	19	14.6%	-0.43 [-0.93, 0.08]
**Subtotal (95% CI)**			**94**			**33**	**21.6%**	**-0.29 [-0.70, 0.13]**
Heterogeneity: Chi² = 0.89, df = 1 (P = 0.35); I² = 0%								
Test for overall effect: Z = 1.36 (P = 0.17)								
**Total (95% CI)**			**261**			**198**	**100.0%**	**-0.25 [-0.44, -0.06]**
Heterogeneity: Chi² = 9.06, df = 8 (P = 0.34); I² = 12%								
Test for overall effect: Z = 2.56 (P = 0.01)								
Test for subgroup differences: Chi² = 0.04, df = 1 (P = 0.85), I² = 0%								

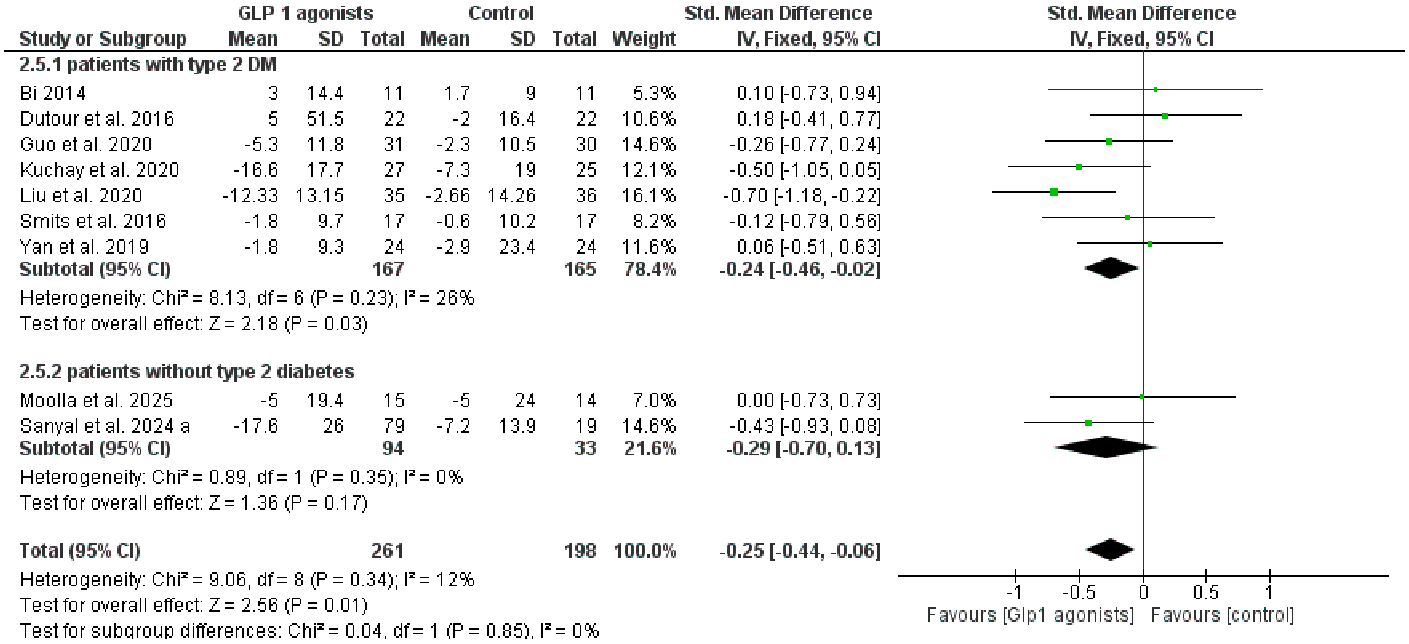

Supplementary Figure S31. Forest plot of the change in AST in patients with and without type 2 diabetes.

	Glp1 agonists			Control				Std. Mean Difference
Study or Subgroup	Mean	SD	Total	Mean	SD	Total	Weight	IV, Random, 95% CI
Armstrong et al. 2016	-26.6	34.4	23	-10.2	35.8	22	5.6%	-0.46 [-1.05, 0.13]
Bi 2014	-10.5	21.1	11	0.2	9.8	11	4.6%	-0.63 [-1.49, 0.23]
Dutour et al. 2016	-3	46.1	22	-1	38.2	22	5.6%	-0.05 [-0.64, 0.54]
Guo et al. 2020	-6	14.5	31	-0.9	6.5	60	6.2%	-0.51 [-0.95, -0.07]
Harrison et al. 2025	-12.9	14.6	70	-6.2	14.3	24	6.1%	-0.46 [-0.93, 0.01]
Khoo et al. 2019	-26	33	15	-39	35	15	5.1%	0.37 [-0.35, 1.09]
Kuchay et al. 2020	-26.4	28.6	27	-13.9	25	25	5.8%	-0.46 [-1.01, 0.09]
Liu et al. 2020	-21.52	20.02	35	-7.66	19.89	36	6.1%	-0.69 [-1.17, -0.21]
Loomba et al. 2023	-20.2	44.5	47	1.9	38.2	24	6.0%	-0.51 [-1.01, -0.01]
Loomba et al. 2024	-34.2	17	142	-3.3	37.6	48	6.5%	-1.29 [-1.64, -0.94]
Moolla et al. 2025	-12	38.7	15	-14	42.3	14	5.1%	0.05 [-0.68, 0.78]
Newsome et al. 2021	-24.9	6.6	240	-10.96	4.7	75	6.6%	-2.24 [-2.56, -1.93]
Sanyal et al. 2024 a	-24.7	37	79	-13.5	18.7	19	6.0%	-0.32 [-0.83, 0.18]
Sanyal et al. 2024 b	-37.4	42.1	158	-5.7	23.22	64	6.6%	-0.84 [-1.14, -0.54]
Sanyal et al. 2025	-37.2	48.5	534	-14	59.9	266	7.0%	-0.44 [-0.59, -0.29]
Smits et al. 2016	0.5	17.8	17	-3.5	26.7	17	5.3%	0.17 [-0.50, 0.85]
Yan et al. 2019	-5.2	12.5	24	-0.8	18.2	24	5.7%	-0.28 [-0.85, 0.29]
**Total (95% CI)**			**1490**			**766**	**100.0%**	**-0.54 [-0.85, -0.23]**
Heterogeneity: Tau² = 0.36; Chi² = 147.12, df = 16 (P < 0.00001); I² = 89%								
Test for overall effect: Z = 3.37 (P = 0.0008)								

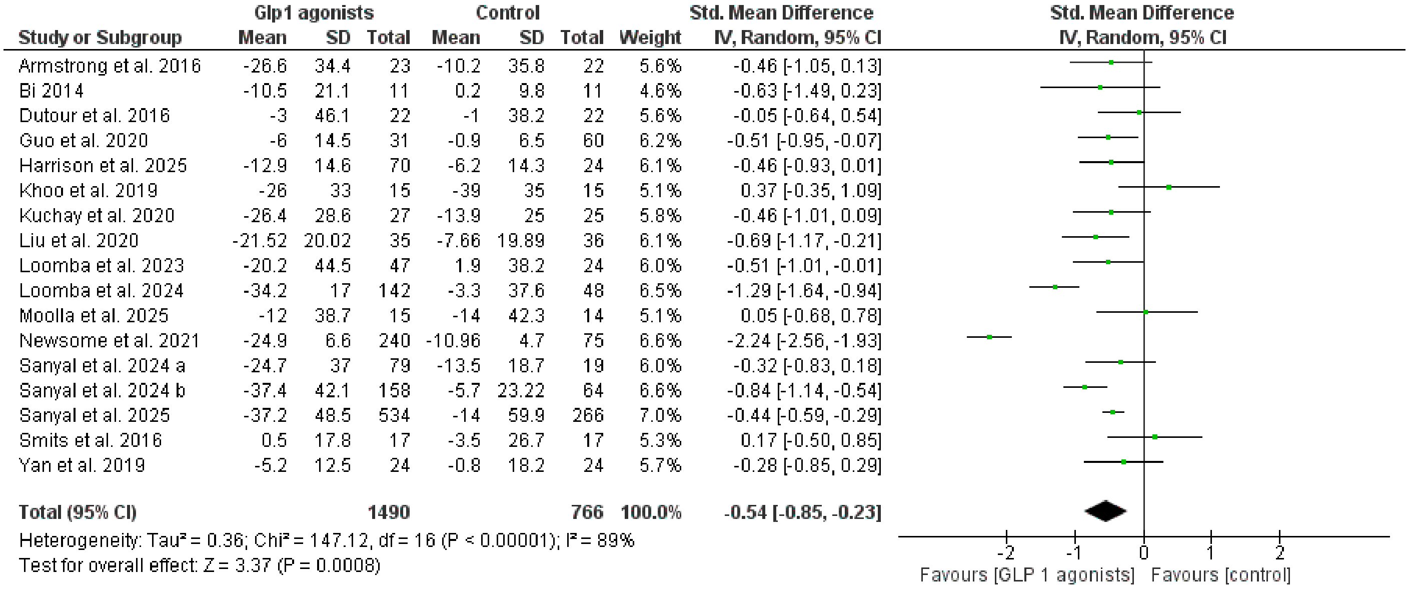

Supplementary Figure S32. Forest plot of the change in ALT in the overall population.

Study or Subgroup	Glp1 agonists Mean	SD	Total	Control Mean	SD	Total	Weight	Std. Mean Difference IV, Random, 95% CI
**4.10.1 Placebo**								
Armstrong et al. 2016	-26.6	34.4	23	-10.2	35.8	22	5.3%	-0.46 [-1.05, 0.13]
Guo et al. 2020	-6	14.5	31	0	0.7	30	5.6%	-0.57 [-1.09, -0.06]
Harrison et al. 2025	-12.9	14.6	70	-6.2	14.3	24	5.8%	-0.46 [-0.93, 0.01]
Loomba et al. 2023	-20.2	44.5	47	1.9	38.2	24	5.7%	-0.51 [-1.01, -0.01]
Loomba et al. 2024	-34.2	17	142	-3.3	37.6	48	6.1%	-1.29 [-1.64, -0.94]
Newsome et al. 2021	-24.9	6.6	240	-10.96	4.7	75	6.3%	-2.24 [-2.56, -1.93]
Sanyal et al. 2024 a	-24.7	37	79	-13.5	18.7	19	5.6%	-0.32 [-0.83, 0.18]
Sanyal et al. 2024 b	-37.4	42.1	158	-5.7	23.22	64	6.3%	-0.84 [-1.14, -0.54]
Sanyal et al. 2025	-37.2	48.5	534	-14	59.9	266	6.6%	-0.44 [-0.59, -0.29]
Smits et al. 2016	0.5	17.8	17	-3.5	26.7	17	5.0%	0.17 [-0.50, 0.85]
**Subtotal (95% CI)**			**1341**			**589**	**58.3%**	**-0.72 [-1.15, -0.29]**
Heterogeneity: Tau² = 0.43; Chi² = 126.56, df = 9 (P < 0.00001); I² = 93%								
Test for overall effect: Z = 3.27 (P = 0.001)								
**4.10.2 Insulin**								
Bi 2014	-10.5	21.1	11	0.2	9.8	11	4.3%	-0.63 [-1.49, 0.23]
Guo et al. 2020	-6	14.5	31	-0.9	6.5	60	5.9%	-0.51 [-0.95, -0.07]
Liu et al. 2020	-21.52	20.02	35	-7.66	19.89	36	5.7%	-0.69 [-1.17, -0.21]
Yan et al. 2019	-5.2	12.5	24	-0.8	18.2	24	5.4%	-0.28 [-0.85, 0.29]
**Subtotal (95% CI)**			**101**			**131**	**21.3%**	**-0.52 [-0.79, -0.26]**
Heterogeneity: Tau² = 0.00; Chi² = 1.23, df = 3 (P = 0.75); I² = 0%								
Test for overall effect: Z = 3.84 (P = 0.0001)								
**4.10.3 usual care or lifestyle interventions**								
Dutour et al. 2016	-3	46.1	22	-1	38.2	22	5.3%	-0.05 [-0.64, 0.54]
Khoo et al. 2019	-26	33	15	-39	35	15	4.8%	0.37 [-0.35, 1.09]
Kuchay et al. 2020	-26.4	28.6	27	-13.9	25	25	5.5%	-0.46 [-1.01, 0.09]
Moolla et al. 2025	-12	38.7	15	-14	42.3	14	4.8%	0.05 [-0.68, 0.78]
**Subtotal (95% CI)**			**79**			**76**	**20.4%**	**-0.08 [-0.42, 0.26]**
Heterogeneity: Tau² = 0.02; Chi² = 3.43, df = 3 (P = 0.33); I² = 12%								
Test for overall effect: Z = 0.44 (P = 0.66)								
**Total (95% CI)**			**1521**			**796**	**100.0%**	**-0.54 [-0.84, -0.24]**
Heterogeneity: Tau² = 0.34; Chi² = 147.24, df = 17 (P < 0.00001); I² = 88%								
Test for overall effect: Z = 3.57 (P = 0.0004)								
Test for subgroup differences: Chi² = 6.37, df = 2 (P = 0.04), I² = 68.6%								

Std. Mean Difference
IV, Random, 95% CI

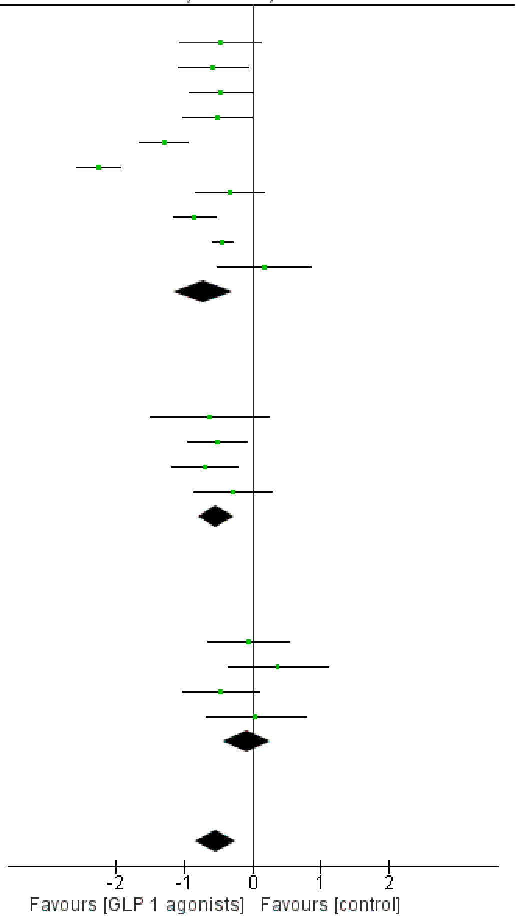

Supplementary Figure S33. Forest plot of the change in ALT based on the control group.

Study or Subgroup	Glp1 agonists Mean	SD	Total	Control Mean	SD	Total	Weight	Std. Mean Difference IV, Random, 95% CI
**3.8.1 Single agonists**								
Armstrong et al. 2016	-26.6	34.4	23	-10.2	35.8	22	5.4%	-0.46 [-1.05, 0.13]
Bi 2014	-10.5	21.1	11	0.2	9.8	11	3.4%	-0.63 [-1.49, 0.23]
Dutour et al. 2016	-3	46.1	22	-1	38.2	22	5.4%	-0.05 [-0.64, 0.54]
Guo et al. 2020	-6	14.5	31	-0.9	6.5	60	7.1%	-0.51 [-0.95, -0.07]
Khoo et al. 2019	-26	33	15	-39	35	15	4.3%	0.37 [-0.35, 1.09]
Kuchay et al. 2020	-26.4	28.6	27	-13.9	25	25	5.8%	-0.46 [-1.01, 0.09]
Liu et al. 2020	-21.52	20.02	35	-7.66	19.89	36	6.6%	-0.69 [-1.17, -0.21]
Loomba et al. 2023	-20.2	44.5	47	1.9	38.2	24	6.4%	-0.51 [-1.01, -0.01]
Moolla et al. 2025	-12	38.7	15	-14	42.3	14	4.2%	0.05 [-0.68, 0.78]
Newsome et al. 2021	-24.9	6.6	240	-10.96	4.7	75		Not estimable
Sanyal et al. 2025	-37.2	48.5	534	-14	59.9	266	10.8%	-0.44 [-0.59, -0.29]
Smits et al. 2016	0.5	17.8	17	-3.5	26.7	17	4.7%	0.17 [-0.50, 0.85]
Yan et al. 2019	-5.2	12.5	24	-0.8	18.2	24	5.6%	-0.28 [-0.85, 0.29]
**Subtotal (95% CI)**			**801**			**536**	**69.7%**	**-0.37 [-0.52, -0.23]**
Heterogeneity: Tau² = 0.01; Chi² = 12.60, df = 11 (P = 0.32); I² = 13%								
Test for overall effect: Z = 5.09 (P < 0.00001)								
**3.8.2 Dual or triple agonists**								
Harrison et al. 2025	-12.9	14.6	70	-6.2	14.3	24	6.7%	-0.46 [-0.93, 0.01]
Loomba et al. 2024	-34.2	17	142	-3.3	37.6	48	8.2%	-1.29 [-1.64, -0.94]
Sanyal et al. 2024 a	-24.7	37	79	-13.5	18.7	19	6.3%	-0.32 [-0.83, 0.18]
Sanyal et al. 2024 b	-37.4	42.1	158	-5.7	23.22	64	9.0%	-0.84 [-1.14, -0.54]
**Subtotal (95% CI)**			**449**			**155**	**30.3%**	**-0.76 [-1.17, -0.35]**
Heterogeneity: Tau² = 0.13; Chi² = 12.83, df = 3 (P = 0.005); I² = 77%								
Test for overall effect: Z = 3.63 (P = 0.0003)								
**Total (95% CI)**			**1250**			**691**	**100.0%**	**-0.46 [-0.65, -0.27]**
Heterogeneity: Tau² = 0.08; Chi² = 40.32, df = 15 (P = 0.0004); I² = 63%								
Test for overall effect: Z = 4.79 (P < 0.00001)								
Test for subgroup differences: Chi² = 3.00, df = 1 (P = 0.08), I² = 66.7%								

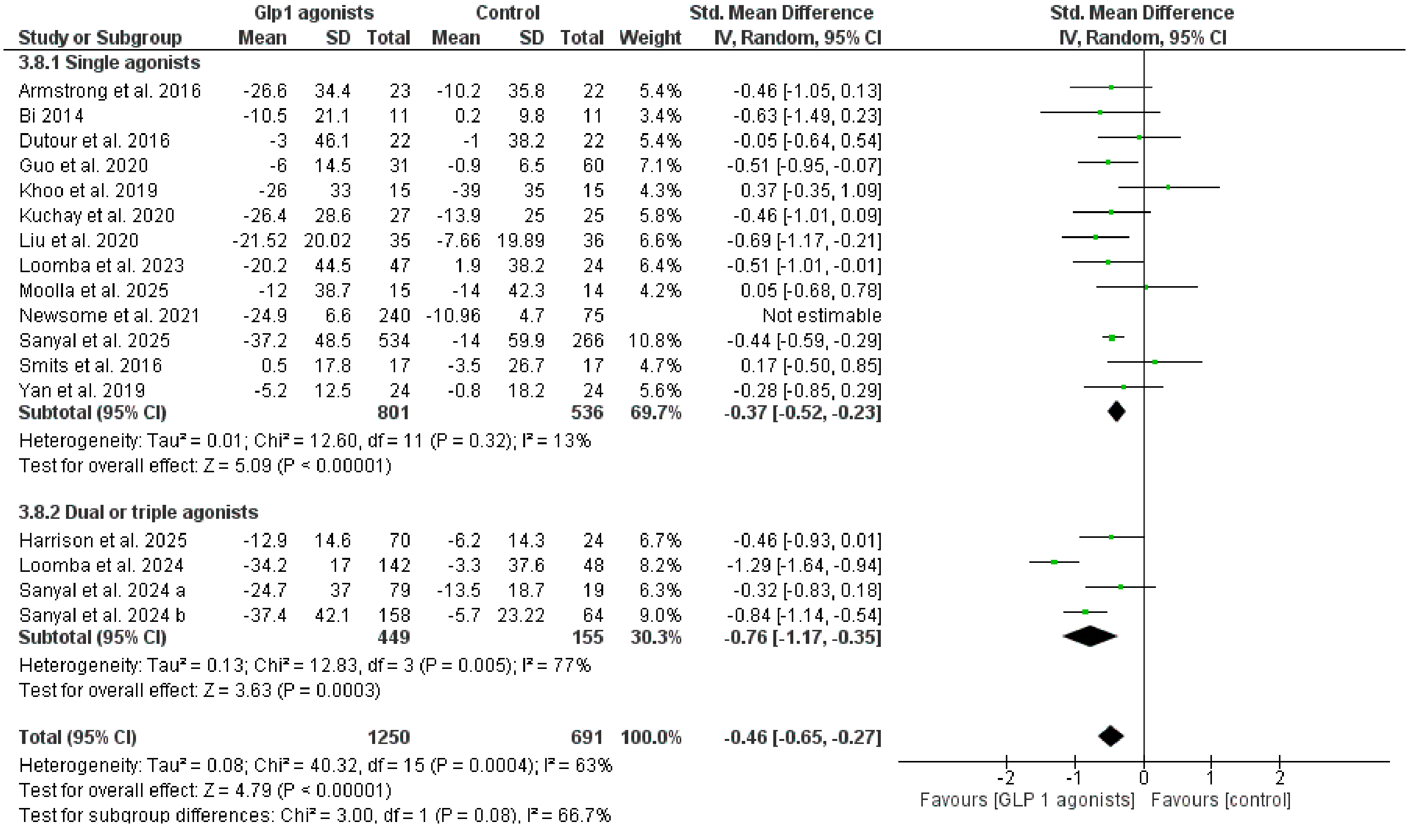

Supplementary Figure S34. Forest plot of the change in ALT based on the intervention group (single, or dual or triple agonists).

Study or Subgroup	Glp1 agonists Mean	SD	Total	Control Mean	SD	Total	Weight	Std. Mean Difference IV, Random, 95% CI
**3.8.1 Single agonists**								
Armstrong et al. 2016	-26.6	34.4	23	-10.2	35.8	22	4.8%	-0.46 [-1.05, 0.13]
Bi 2014	-10.5	21.1	11	0.2	9.8	11	2.6%	-0.63 [-1.49, 0.23]
Dutour et al. 2016	-3	46.1	22	-1	38.2	22	4.9%	-0.05 [-0.64, 0.54]
Guo et al. 2020	-6	14.5	31	-0.9	6.5	60	7.6%	-0.51 [-0.95, -0.07]
Khoo et al. 2019	-26	33	15	-39	35	15	3.5%	0.37 [-0.35, 1.09]
Kuchay et al. 2020	-26.4	28.6	27	-13.9	25	25	5.4%	-0.46 [-1.01, 0.09]
Liu et al. 2020	-21.52	20.02	35	-7.66	19.89	36	6.7%	-0.69 [-1.17, -0.21]
Loomba et al. 2023	-20.2	44.5	47	1.9	38.2	24	6.3%	-0.51 [-1.01, -0.01]
Moolla et al. 2025	-12	38.7	15	-14	42.3	14	3.4%	0.05 [-0.68, 0.78]
Newsome et al. 2021	-24.9	6.6	240	-10.96	4.7	75		Not estimable
Sanyal et al. 2025	-37.2	48.5	534	-14	59.9	266	20.2%	-0.44 [-0.59, -0.29]
Smits et al. 2016	0.5	17.8	17	-3.5	26.7	17	3.9%	0.17 [-0.50, 0.85]
Yan et al. 2019	-5.2	12.5	24	-0.8	18.2	24	5.2%	-0.28 [-0.85, 0.29]
**Subtotal (95% CI)**			**801**			**536**	**74.6%**	**-0.37 [-0.52, -0.23]**
Heterogeneity: Tau² = 0.01; Chi² = 12.60, df = 11 (P = 0.32); I² = 13%								
Test for overall effect: Z = 5.09 (P < 0.00001)								
**3.8.2 Dual or triple agonists**								
Harrison et al. 2025	-12.9	14.6	70	-6.2	14.3	24	7.0%	-0.46 [-0.93, 0.01]
Loomba et al. 2024	-34.2	17	142	-3.3	37.6	48	0.0%	-1.29 [-1.64, -0.94]
Sanyal et al. 2024 a	-24.7	37	79	-13.5	18.7	19	6.3%	-0.32 [-0.83, 0.18]
Sanyal et al. 2024 b	-37.4	42.1	158	-5.7	23.22	64	12.2%	-0.84 [-1.14, -0.54]
**Subtotal (95% CI)**			**307**			**107**	**25.4%**	**-0.59 [-0.92, -0.26]**
Heterogeneity: Tau² = 0.04; Chi² = 3.77, df = 2 (P = 0.15); I² = 47%								
Test for overall effect: Z = 3.51 (P = 0.0004)								
**Total (95% CI)**			**1108**			**643**	**100.0%**	**-0.42 [-0.56, -0.27]**
Heterogeneity: Tau² = 0.02; Chi² = 20.08, df = 14 (P = 0.13); I² = 30%								
Test for overall effect: Z = 5.67 (P < 0.00001)								
Test for subgroup differences: Chi² = 1.40, df = 1 (P = 0.24), I² = 28.6%								

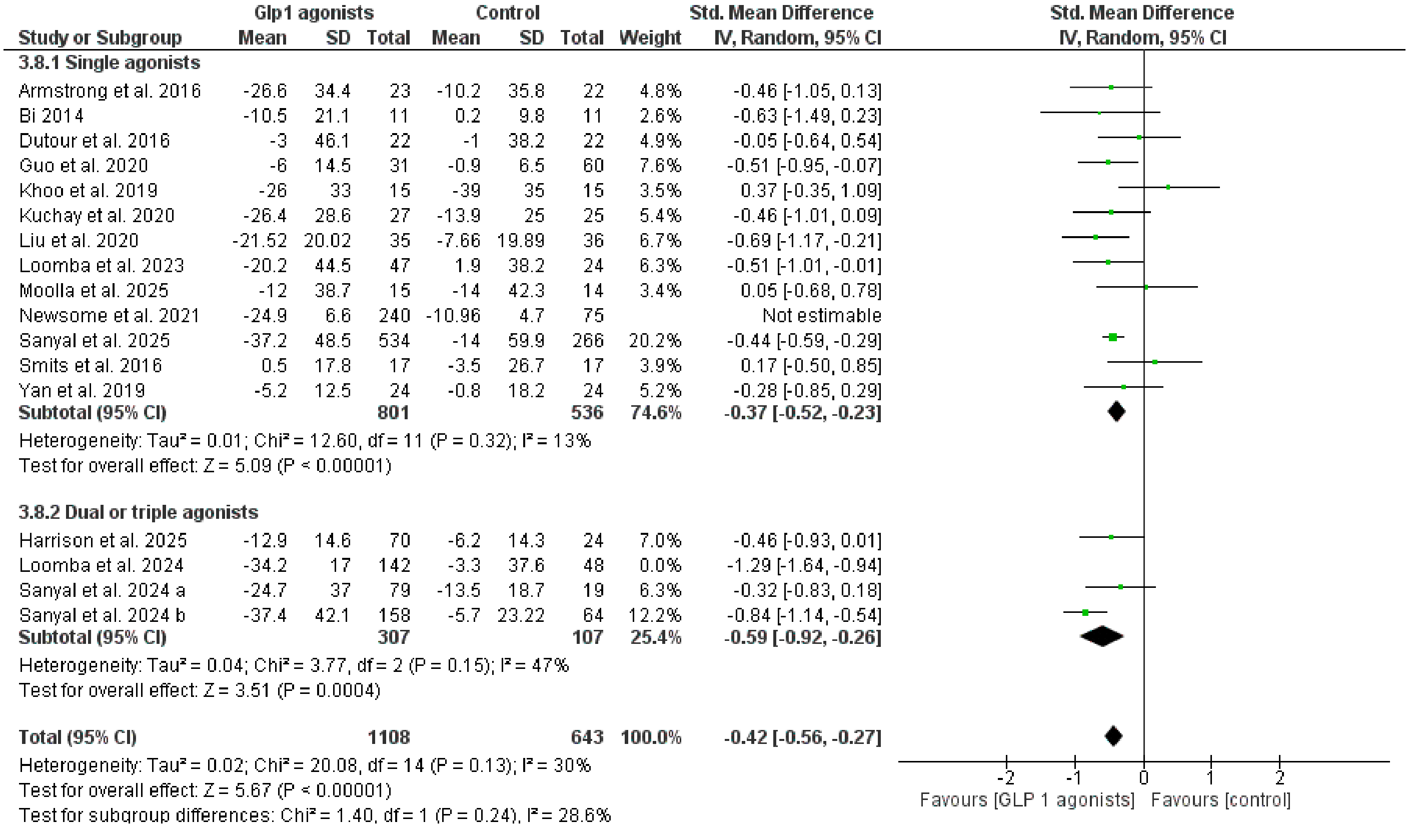

Supplementary Figure S35. Forest plot of the change in ALT based on the intervention group (single, or dual or triple agonists) after Leave-one-out test.

Study or Subgroup	GLP 1 agonists Mean	SD	Total	Control Mean	SD	Total	Weight	Std. Mean Difference IV, Fixed, 95% CI
**2.6.1 patients with type 2 DM**								
Bi 2014	-10.5	21.1	11	0.2	9.8	11	5.0%	-0.63 [-1.49, 0.23]
Dutour et al. 2016	-3	46.1	22	-1	38.2	22	10.6%	-0.05 [-0.64, 0.54]
Guo et al. 2020	-6	14.5	31	-1.8	12.3	30	14.6%	-0.31 [-0.81, 0.20]
Kuchay et al. 2020	-26.4	28.6	27	-13.9	25	25	12.2%	-0.46 [-1.01, 0.09]
Liu et al. 2020	-21.52	20.02	35	-7.66	19.89	36	16.2%	-0.69 [-1.17, -0.21]
Smits et al. 2016	0.5	17.8	17	-3.5	26.7	17	8.2%	0.17 [-0.50, 0.85]
Yan et al. 2019	-5.2	12.5	24	-0.8	18.2	24	11.5%	-0.28 [-0.85, 0.29]
**Subtotal (95% CI)**			**167**			**165**	**78.3%**	**-0.34 [-0.56, -0.12]**
Heterogeneity: Chi² = 5.84, df = 6 (P = 0.44); I² = 0%								
Test for overall effect: Z = 3.05 (P = 0.002)								
**2.6.2 patients without type 2 diabetes**								
Moolla et al. 2025	-12	38.7	15	-14	42.3	14	7.0%	0.05 [-0.68, 0.78]
Sanyal et al. 2024 a	-24.7	37	79	-13.5	18.7	19	14.7%	-0.32 [-0.83, 0.18]
**Subtotal (95% CI)**			**94**			**33**	**21.7%**	**-0.20 [-0.62, 0.21]**
Heterogeneity: Chi² = 0.68, df = 1 (P = 0.41); I² = 0%								
Test for overall effect: Z = 0.96 (P = 0.33)								
**Total (95% CI)**			**261**			**198**	**100.0%**	**-0.31 [-0.50, -0.12]**
Heterogeneity: Chi² = 6.84, df = 8 (P = 0.55); I² = 0%								
Test for overall effect: Z = 3.15 (P = 0.002)								
Test for subgroup differences: Chi² = 0.32, df = 1 (P = 0.57), I² = 0%								

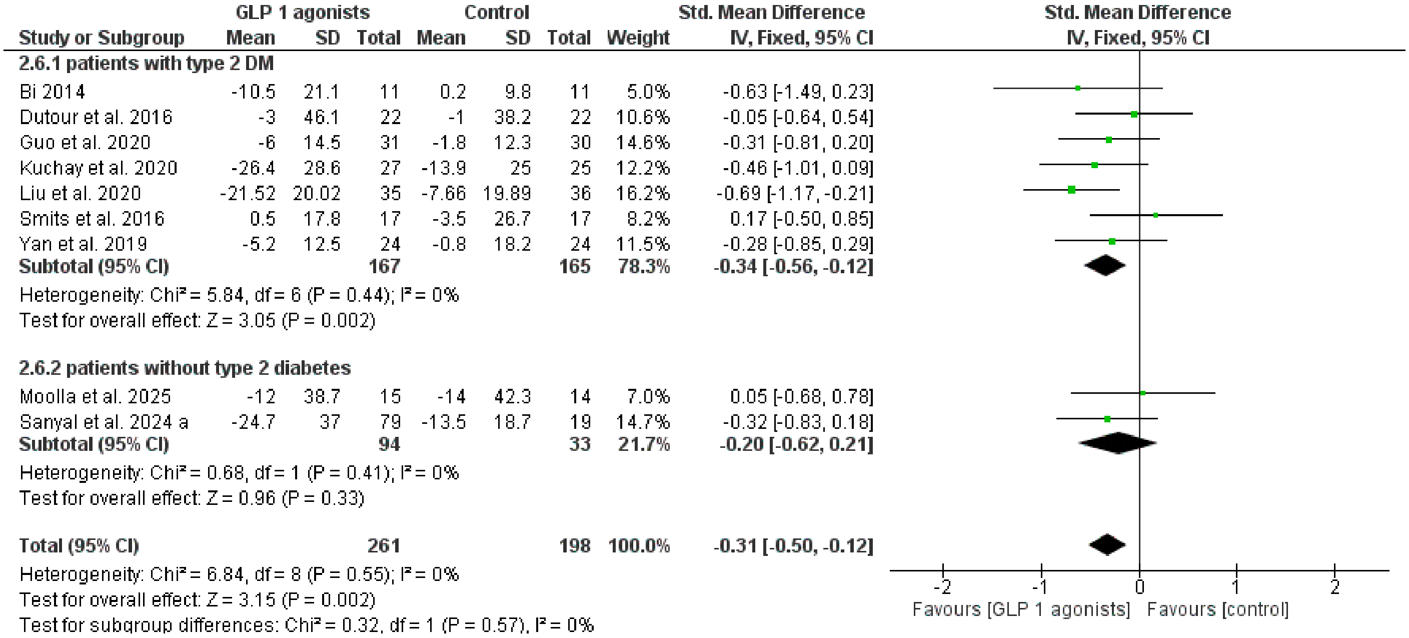

Supplementary Figure S36. Forest plot of the change in ALT in patients with and without type 2 diabetes.

Study or Subgroup	Glp1 agonists Mean	SD	Total	Control Mean	SD	Total	Weight	Std. Mean Difference IV, Fixed, 95% CI
Armstrong et al. 2016	1.9	5.1	23	-0.5	8	22	15.7%	0.35 [-0.24, 0.94]
Newsome et al. 2021	2.4	7.5	240	-0.22	7.5	80	84.3%	0.35 [0.09, 0.60]
**Total (95% CI)**			**263**			**102**	**100.0%**	**0.35 [0.12, 0.58]**

Heterogeneity: Chi² = 0.00, df = 1 (P = 0.99); I² = 0%
Test for overall effect: Z = 2.93 (P = 0.003)

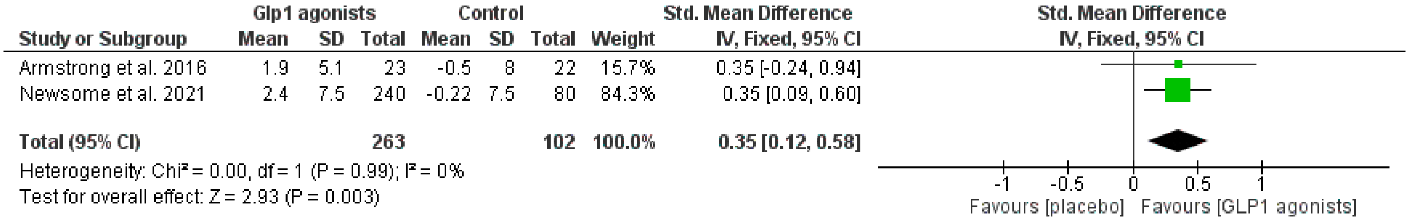

Supplementary Figure S37. Forest plot of the change Quality of life SF-36 (physical component).

Study or Subgroup	Glp1 agonists Mean	SD	Total	Control Mean	SD	Total	Weight	Std. Mean Difference IV, Fixed, 95% CI
Armstrong et al. 2016	-2.8	6.9	23	-3.3	12.4	22	15.8%	0.05 [-0.54, 0.63]
Newsome et al. 2021	1.2	8.5	240	-0.17	8.5	80	84.2%	0.16 [-0.09, 0.41]
**Total (95% CI)**			**263**			**102**	**100.0%**	**0.14 [-0.09, 0.38]**

Heterogeneity: Chi² = 0.12, df = 1 (P = 0.73); I² = 0%
Test for overall effect: Z = 1.21 (P = 0.23)

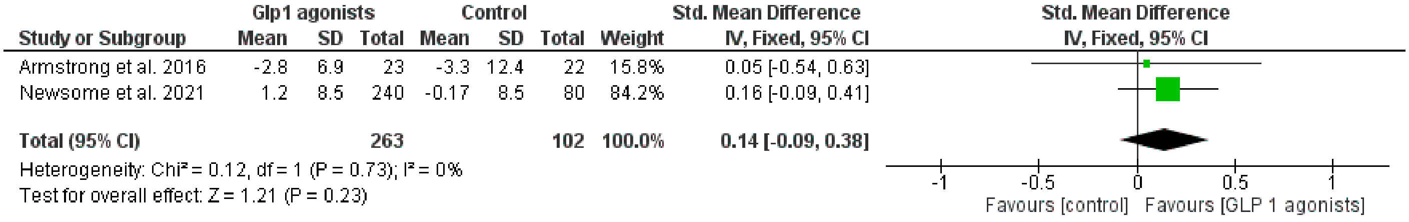

Supplementary Figure S38. Forest plot of the change Quality of life SF-36 (Mental component).

Study or Subgroup	Glp1 agonists Events	Total	Control Events	Total	Weight	Risk Ratio M-H, Fixed, 95% CI
Armstrong et al. 2016	23	26	24	26	3.0%	0.96 [0.80, 1.14]
Flint et al 2021	31	33	29	33	3.6%	1.07 [0.92, 1.25]
Liu et al. 2020	5	35	6	36	0.7%	0.86 [0.29, 2.55]
Loomba et al. 2023	42	47	19	24	3.1%	1.13 [0.90, 1.42]
Loomba et al. 2024	131	142	40	48	7.4%	1.11 [0.97, 1.27]
Newsome et al. 2021	224	239	67	80	12.4%	1.12 [1.01, 1.24]
Sanyal et al. 2024 a	66	79	15	19	3.0%	1.06 [0.82, 1.36]
Sanyal et al. 2024 b	208	219	68	74	12.6%	1.03 [0.96, 1.11]
Sanyal et al. 2025	690	800	315	395	52.3%	1.08 [1.02, 1.14]
Shankar et al. 2024	42	50	9	24	1.5%	2.24 [1.32, 3.81]
Yan et al. 2019	5	24	3	24	0.4%	1.67 [0.45, 6.21]
**Total (95% CI)**		**1694**		**783**	**100.0%**	**1.10 [1.05, 1.14]**
Total events	1467		595			

Heterogeneity: Chi² = 12.86, df = 10 (P = 0.23); I² = 22%
Test for overall effect: Z = 4.53 (P < 0.00001)

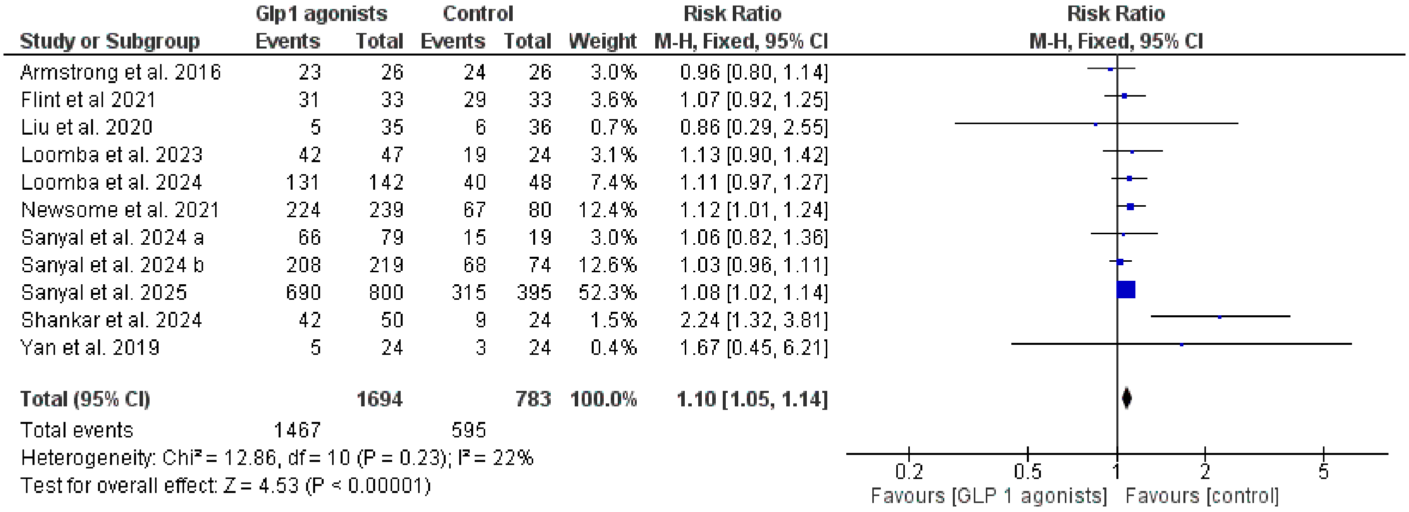

Supplementary Figure S39. Forest plot of the adverse events.

Study or Subgroup	Glp1 agonists Events	Total	Control Events	Total	Weight	Risk Ratio M-H, Fixed, 95% CI
Armstrong et al. 2016	21	26	17	26	5.1%	1.24 [0.88, 1.73]
Kuchay et al. 2020	5	27	2	25	0.6%	2.31 [0.49, 10.87]
Loomba et al. 2023	36	47	8	24	3.2%	2.30 [1.28, 4.13]
Newsome et al. 2021	166	239	36	80	16.3%	1.54 [1.19, 1.99]
Sanyal et al. 2025	556	800	185	395	74.8%	1.48 [1.32, 1.66]
**Total (95% CI)**		**1139**		**550**	**100.0%**	**1.51 [1.37, 1.67]**
Total events	784		248			

Heterogeneity: Chi² = 3.75, df = 4 (P = 0.44); I² = 0%
Test for overall effect: Z = 8.14 (P < 0.00001)

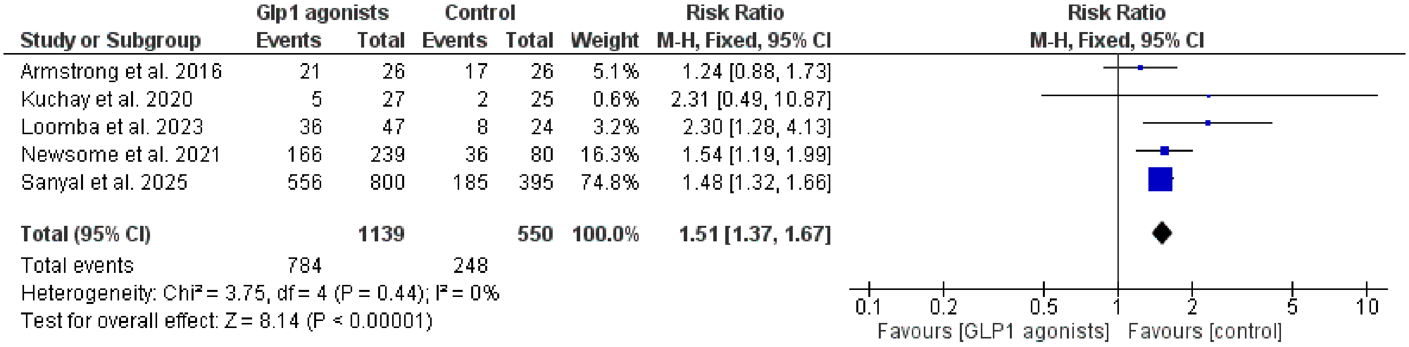

Supplementary Figure S40. Forest plot of the gastrointestinal adverse events.

Study or Subgroup	Glp1 agonists Events	Glp1 agonists Total	Control Events	Control Total	Weight	Risk Ratio M-H, Fixed, 95% CI
Armstrong et al. 2016	10	26	5	26	3.4%	2.00 [0.79, 5.05]
Flint et al 2021	10	33	8	33	5.4%	1.25 [0.56, 2.77]
Guo et al. 2020	1	31	0	60	0.2%	5.72 [0.24, 136.41]
Harrison et al. 2025	9	70	4	24	4.0%	0.77 [0.26, 2.28]
Khoo et al. 2019	5	15	0	15	0.3%	11.00 [0.66, 182.87]
Kuchay et al. 2020	1	27	0	25	0.3%	2.79 [0.12, 65.38]
Loomba et al. 2023	9	47	2	24	1.8%	2.30 [0.54, 9.81]
Loomba et al. 2024	45	142	11	48	11.1%	1.38 [0.78, 2.45]
Newsome et al. 2021	61	239	11	80	11.1%	1.86 [1.03, 3.35]
Sanyal et al. 2024 a	13	79	1	19	1.1%	3.13 [0.44, 22.45]
Sanyal et al. 2024 b	107	219	17	74	17.1%	2.13 [1.37, 3.30]
Sanyal et al. 2025	215	800	48	395	43.3%	2.21 [1.66, 2.95]
Shankar et al. 2024	8	50	1	24	0.9%	3.84 [0.51, 28.98]
**Total (95% CI)**		**1778**		**847**	**100.0%**	**2.02 [1.67, 2.43]**
Total events	494		108			

Heterogeneity: Chi² = 9.07, df = 12 (P = 0.70); I² = 0%
Test for overall effect: Z = 7.29 (P < 0.00001)

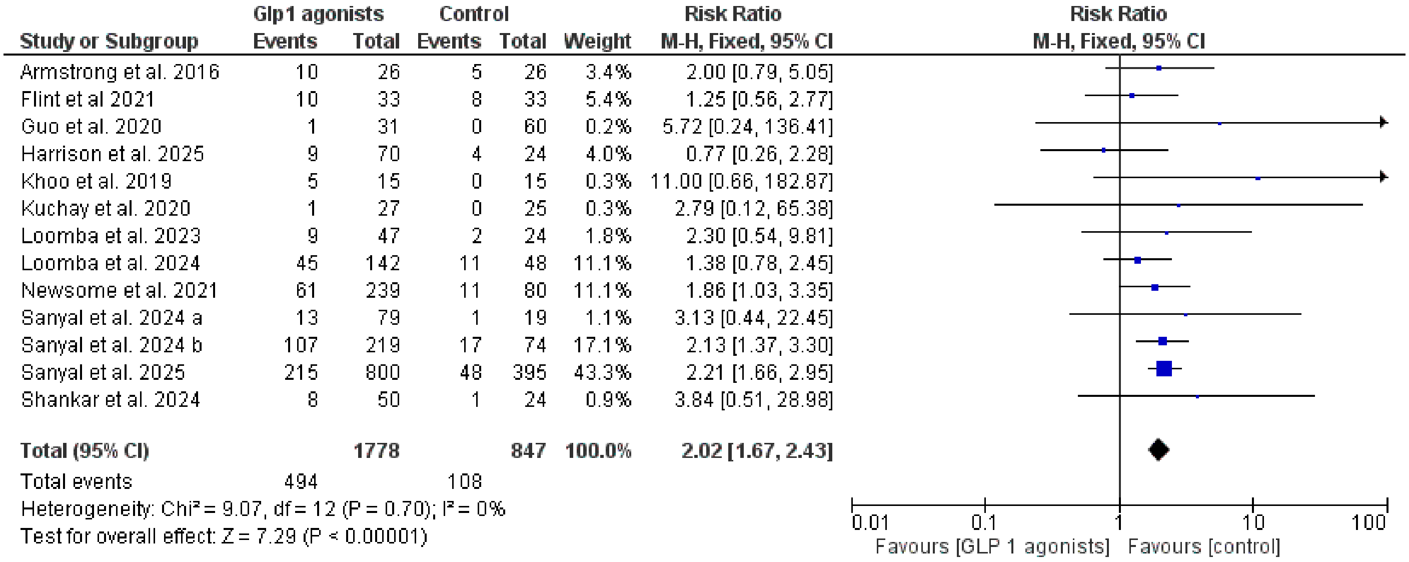

Supplementary Figure S41. Forest plot of diarrhea.

Study or Subgroup	Glp1 agonists Events	Glp1 agonists Total	Control Events	Control Total	Weight	Risk Ratio M-H, Fixed, 95% CI
Armstrong et al. 2016	12	26	10	26	6.7%	1.20 [0.63, 2.27]
Flint et al 2021	10	33	3	33	2.0%	3.33 [1.01, 11.03]
Guo et al. 2020	8	31	3	60	1.4%	5.16 [1.47, 18.09]
Harrison et al. 2025	25	70	3	24	3.0%	2.86 [0.95, 8.62]
Khoo et al. 2019	12	15	0	15	0.3%	25.00 [1.61, 387.35]
Loomba et al. 2023	21	47	4	24	3.6%	2.68 [1.04, 6.93]
Loomba et al. 2024	54	142	6	48	6.0%	3.04 [1.40, 6.62]
Newsome et al. 2021	87	239	9	80	9.1%	3.24 [1.71, 6.12]
Sanyal et al. 2024 a	27	79	1	19	1.1%	6.49 [0.94, 44.83]
Sanyal et al. 2024 b	144	219	17	74	17.1%	2.86 [1.87, 4.39]
Sanyal et al. 2025	290	800	52	395	46.7%	2.75 [2.10, 3.61]
Shankar et al. 2024	34	50	3	24	2.7%	5.44 [1.86, 15.95]
Yan et al. 2019	4	24	0	24	0.3%	9.00 [0.51, 158.52]
**Total (95% CI)**		**1775**		**846**	**100.0%**	**2.98 [2.49, 3.58]**
Total events	728		111			

Heterogeneity: Chi² = 13.76, df = 12 (P = 0.32); I² = 13%
Test for overall effect: Z = 11.78 (P < 0.00001)

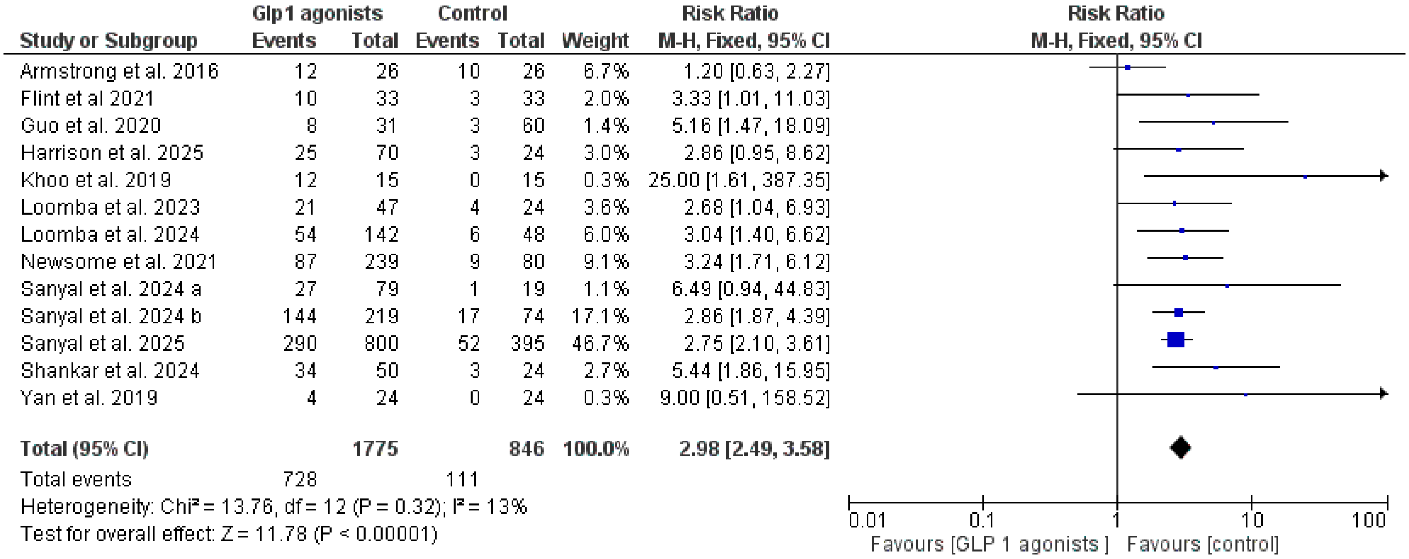

Supplementary Figure S42. Forest plot of nausea.

Study or Subgroup	Glp1 agonists Events	Glp1 agonists Total	Control Events	Control Total	Weight	Risk Ratio M-H, Fixed, 95% CI
Armstrong et al. 2016	5	26	3	26	6.3%	1.67 [0.44, 6.26]
Flint et al 2021	9	33	0	33	1.1%	19.00 [1.15, 313.64]
Guo et al. 2020	8	31	3	60	4.3%	5.16 [1.47, 18.09]
Harrison et al. 2025	7	70	0	24	1.6%	5.28 [0.31, 89.16]
Loomba et al. 2023	8	47	0	24	1.4%	8.85 [0.53, 147.20]
Loomba et al. 2024	13	142	1	48	3.2%	4.39 [0.59, 32.71]
Newsome et al. 2021	43	239	2	80	6.3%	7.20 [1.78, 29.04]
Sanyal et al. 2024 a	13	79	0	19	1.7%	6.75 [0.42, 108.78]
Sanyal et al. 2024 b	89	219	3	74	9.5%	10.02 [3.27, 30.72]
Sanyal et al. 2025	149	800	22	395	62.2%	3.34 [2.17, 5.15]
Shankar et al. 2024	13	50	0	24	1.4%	13.24 [0.82, 213.73]
Yan et al. 2019	4	24	0	24	1.1%	9.00 [0.51, 158.52]
**Total (95% CI)**		**1760**		**831**	**100.0%**	**4.76 [3.40, 6.66]**
Total events	361		34			

Heterogeneity: Chi² = 8.94, df = 11 (P = 0.63); I² = 0%
Test for overall effect: Z = 9.08 (P < 0.00001)

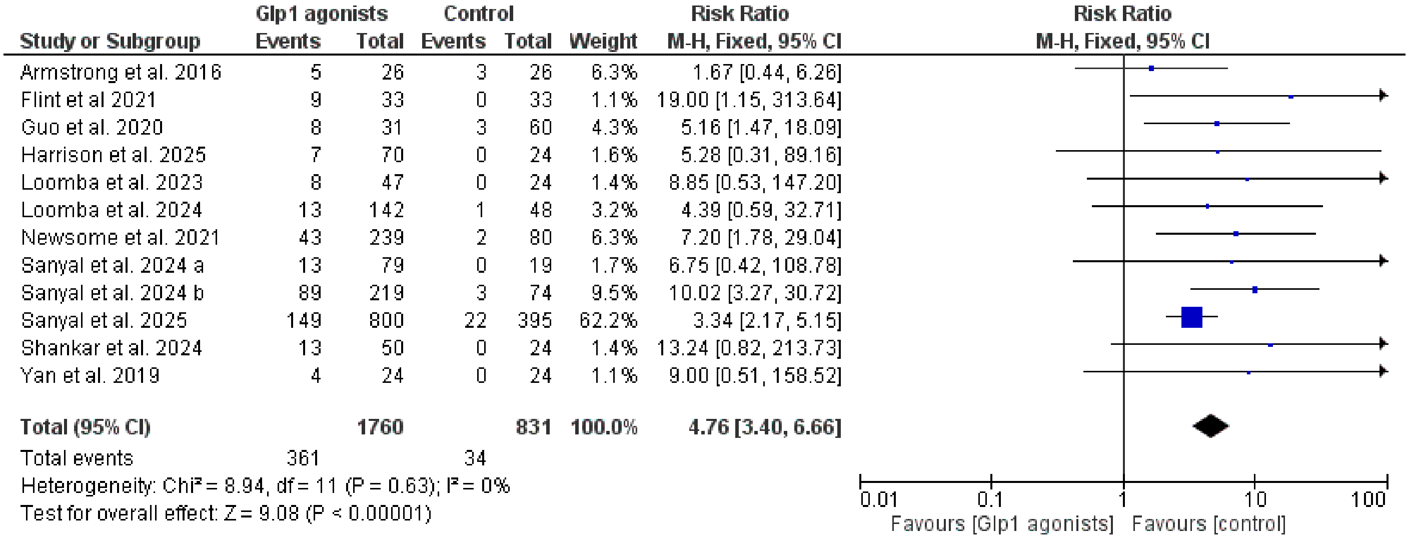

Supplementary Figure S43. Forest plot of vomiting.

Study or Subgroup	Glp1 agonists Events	Glp1 agonists Total	Control Events	Control Total	Weight	Risk Ratio M-H, Fixed, 95% CI
Armstrong et al. 2016	4	26	5	26	8.0%	0.80 [0.24, 2.65]
Flint et al 2021	4	33	3	33	4.8%	1.33 [0.32, 5.50]
Loomba et al. 2024	13	142	3	48	7.2%	1.46 [0.44, 4.92]
Newsome et al. 2021	22	239	7	80	16.9%	1.05 [0.47, 2.37]
Sanyal et al. 2024 a	8	79	0	19	1.3%	4.25 [0.26, 70.58]
Sanyal et al. 2024 b	37	219	6	74	14.4%	2.08 [0.92, 4.74]
Sanyal et al. 2025	71	800	22	395	47.4%	1.59 [1.00, 2.53]
**Total (95% CI)**		**1538**		**675**	**100.0%**	**1.52 [1.10, 2.10]**
Total events	159		46			

Heterogeneity: Chi² = 3.05, df = 6 (P = 0.80); I² = 0%
Test for overall effect: Z = 2.57 (P = 0.01)

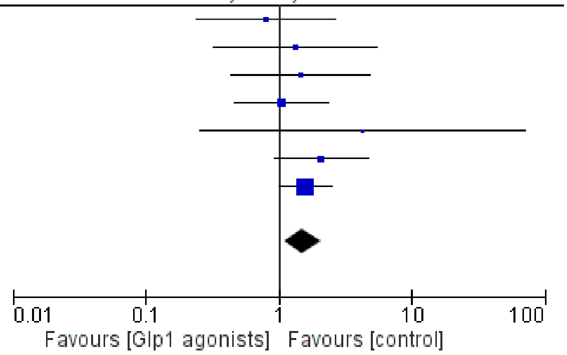

Supplementary Figure S44. Forest plot of fatigue.

Study or Subgroup	Glp1 agonists Events	Glp1 agonists Total	Control Events	Control Total	Weight	Risk Ratio M-H, Fixed, 95% CI
Armstrong et al. 2016	2	26	2	26	1.9%	1.00 [0.15, 6.57]
Flint et al 2021	4	33	3	33	2.8%	1.33 [0.32, 5.50]
Guo et al. 2020	0	31	0	60		Not estimable
Harrison et al. 2025	0	70	0	24		Not estimable
Liu et al. 2020	1	35	0	36	0.5%	3.08 [0.13, 73.23]
Loomba et al. 2023	12	47	4	24	4.9%	1.53 [0.55, 4.24]
Loomba et al. 2024	9	142	3	48	4.2%	1.01 [0.29, 3.59]
Newsome et al. 2021	39	239	8	80	11.1%	1.63 [0.80, 3.34]
Sanyal et al. 2024 a	2	79	0	19	0.7%	1.25 [0.06, 25.02]
Sanyal et al. 2024 b	17	219	5	74	6.9%	1.15 [0.44, 3.01]
Sanyal et al. 2025	107	800	53	395	65.8%	1.00 [0.73, 1.35]
Shankar et al. 2024	2	50	1	24	1.3%	0.96 [0.09, 10.07]
**Total (95% CI)**		**1771**		**843**	**100.0%**	**1.13 [0.88, 1.44]**
Total events	195		79			

Heterogeneity: Chi² = 2.49, df = 9 (P = 0.98); I² = 0%
Test for overall effect: Z = 0.94 (P = 0.35)

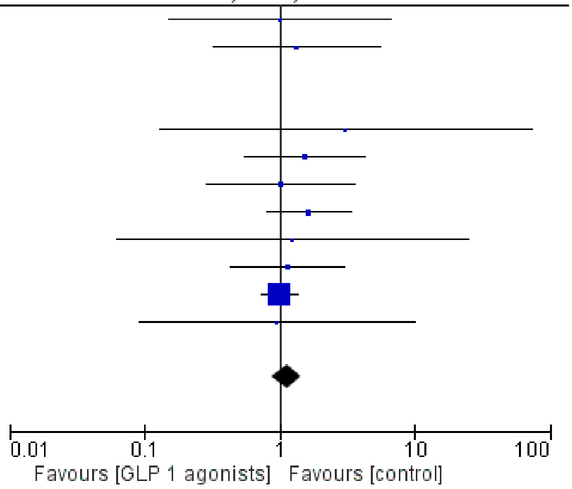

Supplementary Figure S45. Forest plot of serious adverse events.

Study or Subgroup	Glp1 agonists Events	Glp1 agonists Total	Control Events	Control Total	Weight	Risk Ratio M-H, Fixed, 95% CI
Guo et al. 2020	1	31	4	60	6.3%	0.48 [0.06, 4.15]
Liu et al. 2020	3	35	6	36	13.6%	0.51 [0.14, 1.90]
Newsome et al. 2021	39	149	10	50	34.4%	1.31 [0.71, 2.42]
Sanyal et al. 2025	34	800	13	395	40.0%	1.29 [0.69, 2.42]
Yan et al. 2019	0	24	2	24	5.7%	0.20 [0.01, 3.96]
**Total (95% CI)**		**1039**		**565**	**100.0%**	**1.08 [0.72, 1.61]**
Total events	77		35			

Heterogeneity: Chi² = 3.69, df = 4 (P = 0.45); I² = 0%
Test for overall effect: Z = 0.37 (P = 0.71)

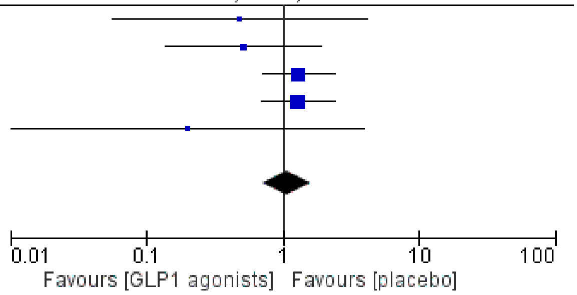

Supplementary Figure S46. Forest plot of hypoglycemia.

Study or Subgroup	Glp1 agonists Events	Glp1 agonists Total	Control Events	Control Total	Weight	Risk Ratio M-H, Fixed, 95% CI
Armstrong et al. 2016	9	26	10	26	24.4%	0.90 [0.44, 1.85]
Newsome et al. 2021	16	239	5	80	18.3%	1.07 [0.41, 2.83]
Sanyal et al. 2025	32	800	17	395	55.6%	0.93 [0.52, 1.65]
Shankar et al. 2024	4	50	0	24	1.6%	4.41 [0.25, 78.77]
**Total (95% CI)**		**1115**		**525**	**100.0%**	**1.01 [0.67, 1.51]**
Total events	61		32			

Heterogeneity: Chi² = 1.19, df = 3 (P = 0.76); I² = 0%
Test for overall effect: Z = 0.03 (P = 0.98)

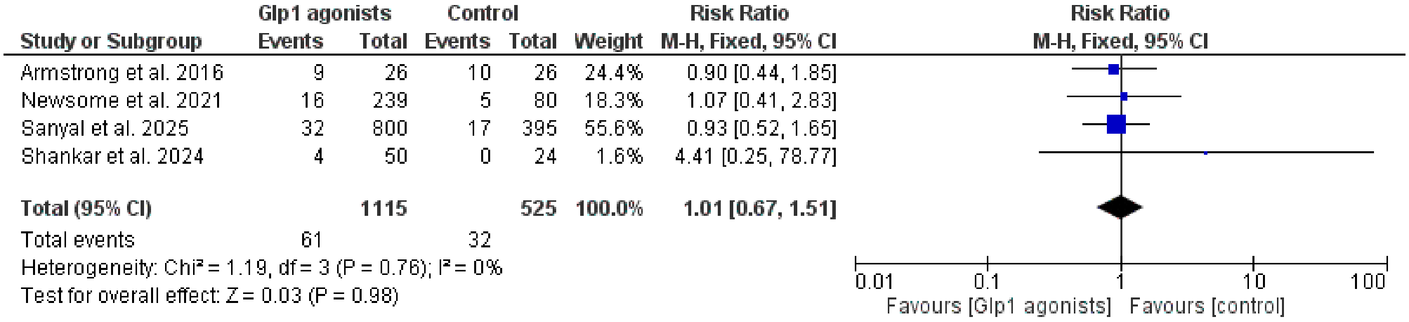

Supplementary Figure S47. Forest plot of injection site reactions.

Study or Subgroup	Glp1 agonists Events	Glp1 agonists Total	Control Events	Control Total	Weight	Risk Ratio M-H, Fixed, 95% CI
Loomba et al. 2024	4	142	1	48	11.2%	1.35 [0.15, 11.80]
Newsome et al. 2021	15	239	2	80	22.5%	2.51 [0.59, 10.74]
Sanyal et al. 2024 a	1	79	0	19	6.0%	0.75 [0.03, 17.73]
Sanyal et al. 2025	20	800	6	395	60.3%	1.65 [0.67, 4.07]
**Total (95% CI)**		**1260**		**542**	**100.0%**	**1.75 [0.87, 3.54]**
Total events	40		9			

Heterogeneity: Chi² = 0.59, df = 3 (P = 0.90); I² = 0%
Test for overall effect: Z = 1.57 (P = 0.12)

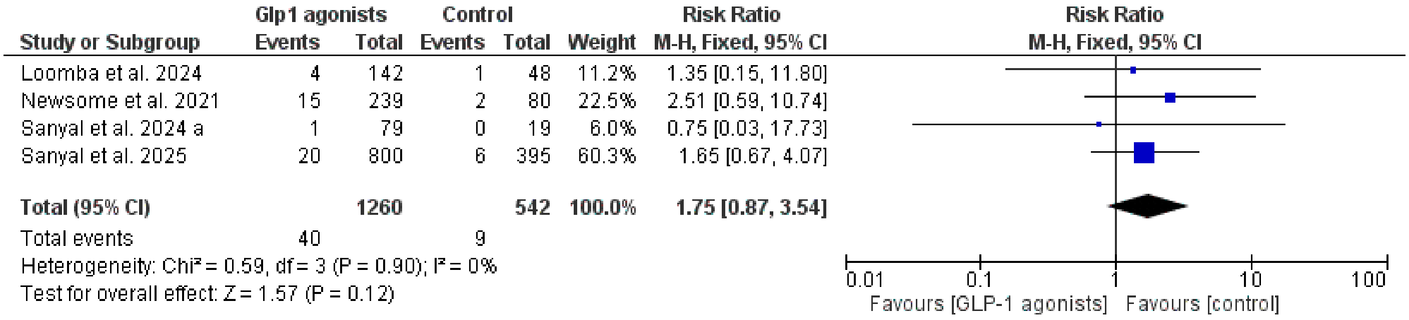

Supplementary Figure S48. Forest plot of gallbladder events.

Study or Subgroup	Glp1 agonists Events	Glp1 agonists Total	Control Events	Control Total	Weight	Risk Ratio M-H, Random, 95% CI
Armstrong et al. 2016	6	26	7	26	23.4%	0.86 [0.33, 2.21]
Flint et al 2021	6	33	3	33	17.6%	2.00 [0.55, 7.33]
Khoo et al. 2019	2	15	0	15	5.5%	5.00 [0.26, 96.13]
Loomba et al. 2024	12	142	2	48	15.4%	2.03 [0.47, 8.74]
Sanyal et al. 2024 a	0	79	5	19	5.8%	0.02 [0.00, 0.39]
Sanyal et al. 2025	42	800	25	395	32.4%	0.83 [0.51, 1.34]
**Total (95% CI)**		**1095**		**536**	**100.0%**	**1.00 [0.47, 2.12]**
Total events	68		42			

Heterogeneity: Tau² = 0.39; Chi² = 10.52, df = 5 (P = 0.06); I² = 52%
Test for overall effect: Z = 0.01 (P = 0.99)

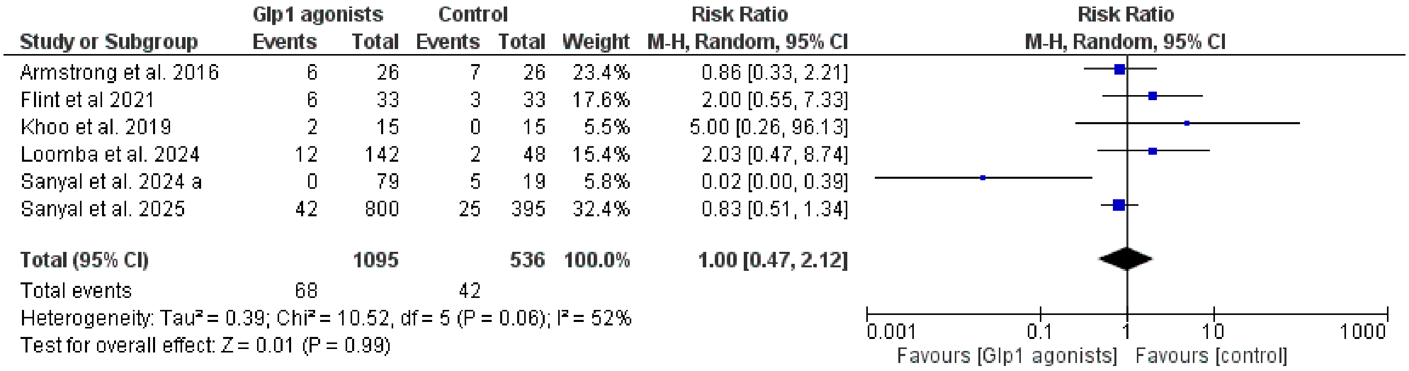

Supplementary Figure S49. Forest plot of dizziness.

Study or Subgroup	Glp1 agonists Events	Glp1 agonists Total	Control Events	Control Total	Weight	Risk Ratio M-H, Random, 95% CI
Armstrong et al. 2016	6	26	7	26	16.9%	0.86 [0.33, 2.21]
Flint et al 2021	6	33	3	33	8.9%	2.00 [0.55, 7.33]
Khoo et al. 2019	2	15	0	15	1.7%	5.00 [0.26, 96.13]
Loomba et al. 2024	12	142	2	48	7.1%	2.03 [0.47, 8.74]
Sanyal et al. 2024 a	0	79	5	19	0.0%	0.02 [0.00, 0.39]
Sanyal et al. 2025	42	800	25	395	65.4%	0.83 [0.51, 1.34]
**Total (95% CI)**		**1016**		**517**	**100.0%**	**0.99 [0.67, 1.46]**
Total events	68		37			

Heterogeneity: Tau² = 0.00; Chi² = 3.85, df = 4 (P = 0.43); I² = 0%
Test for overall effect: Z = 0.04 (P = 0.97)

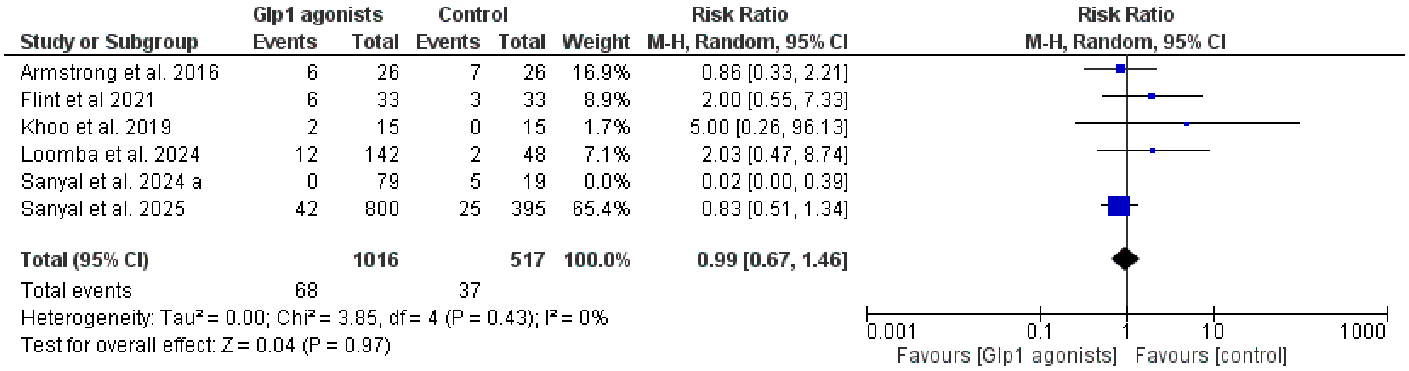

Supplementary Figure S50. Forest plot of dizziness after leaving Sanyal 2024 a.

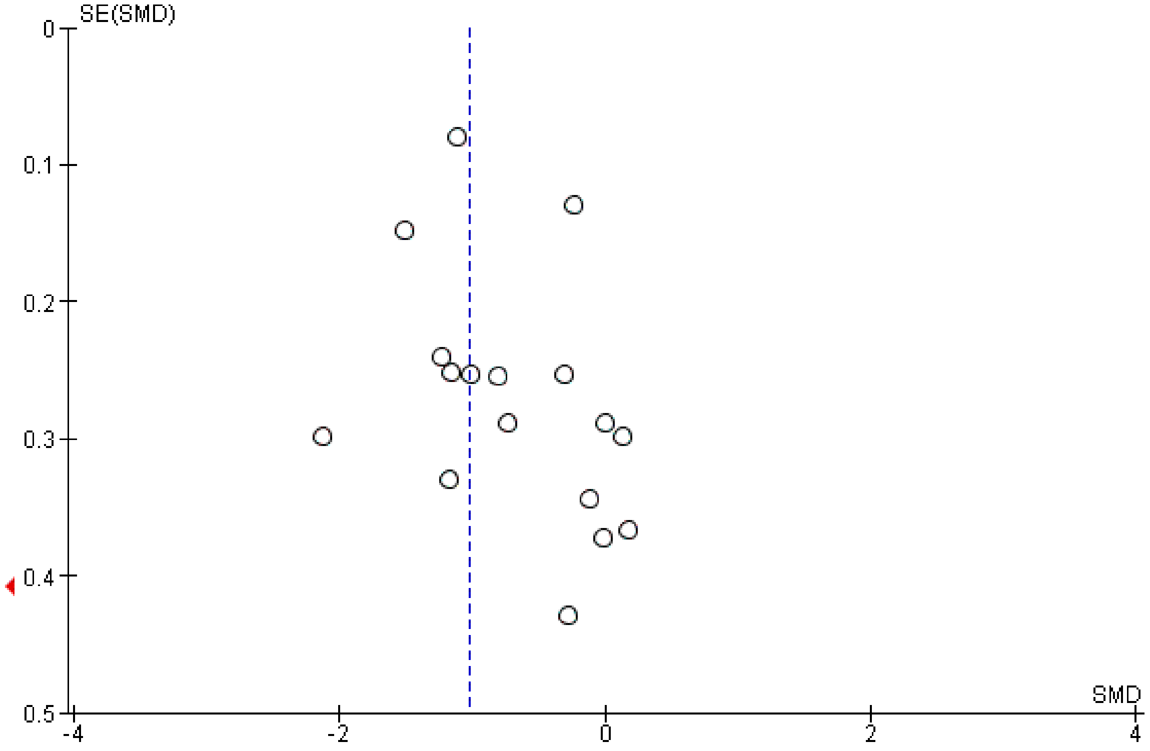

Supplementary Figure S51. Funnel plot of weight

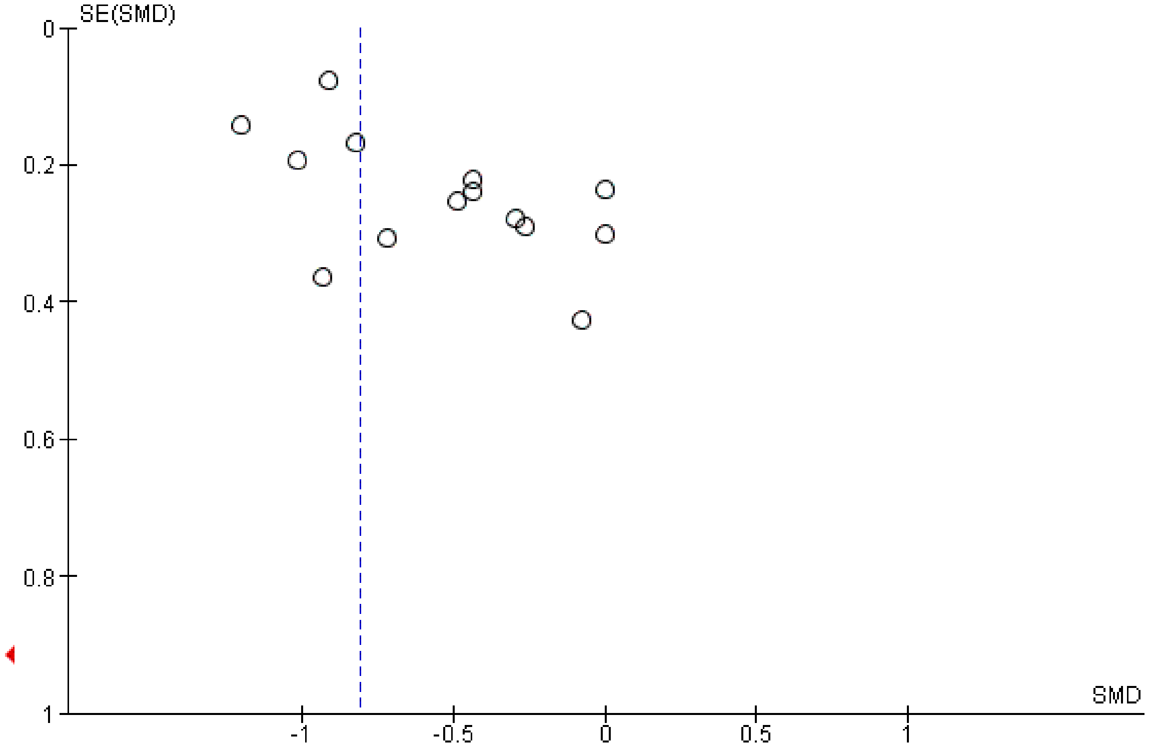

Supplementary Figure S52. Funnel plot of HbA1c

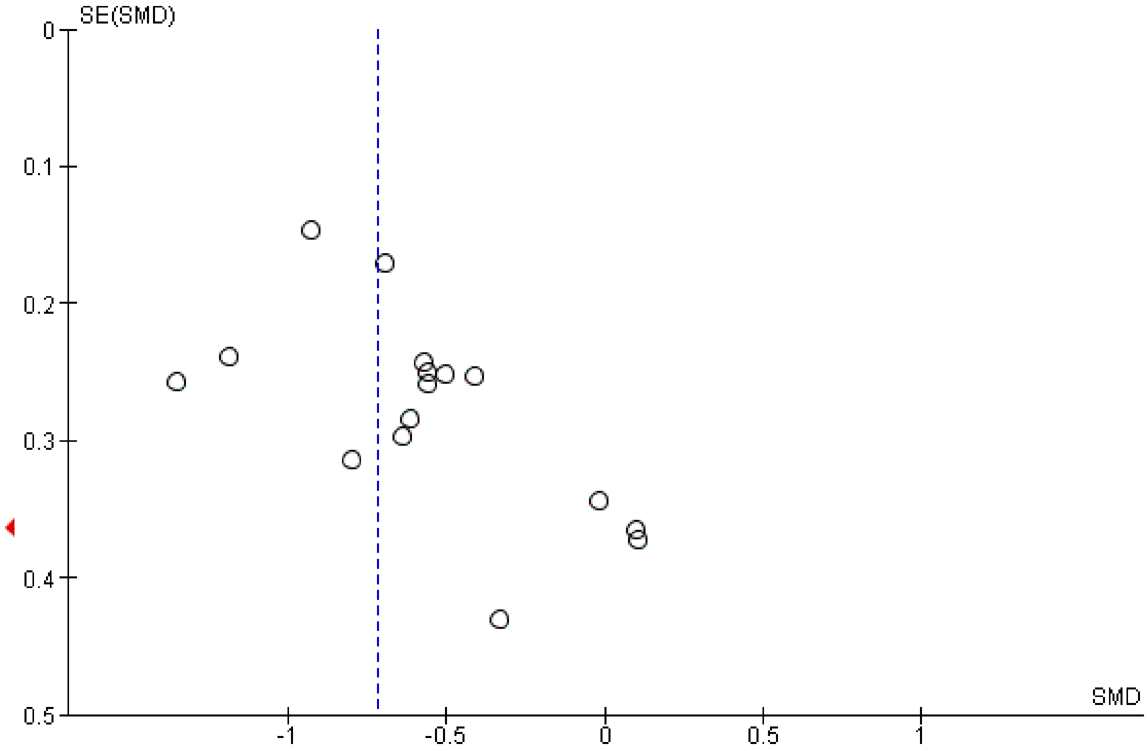

Supplementary Figure S53. Funnel plot of liver fat

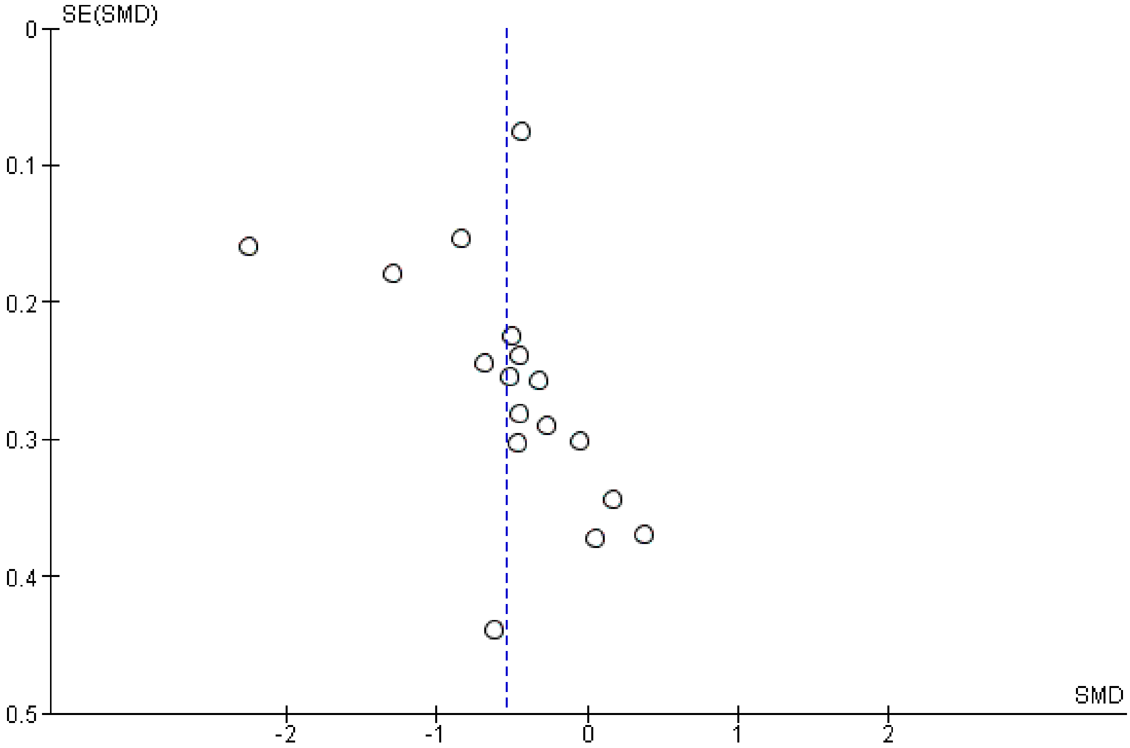

Supplementary Figure S54. Funnel plot of ALT

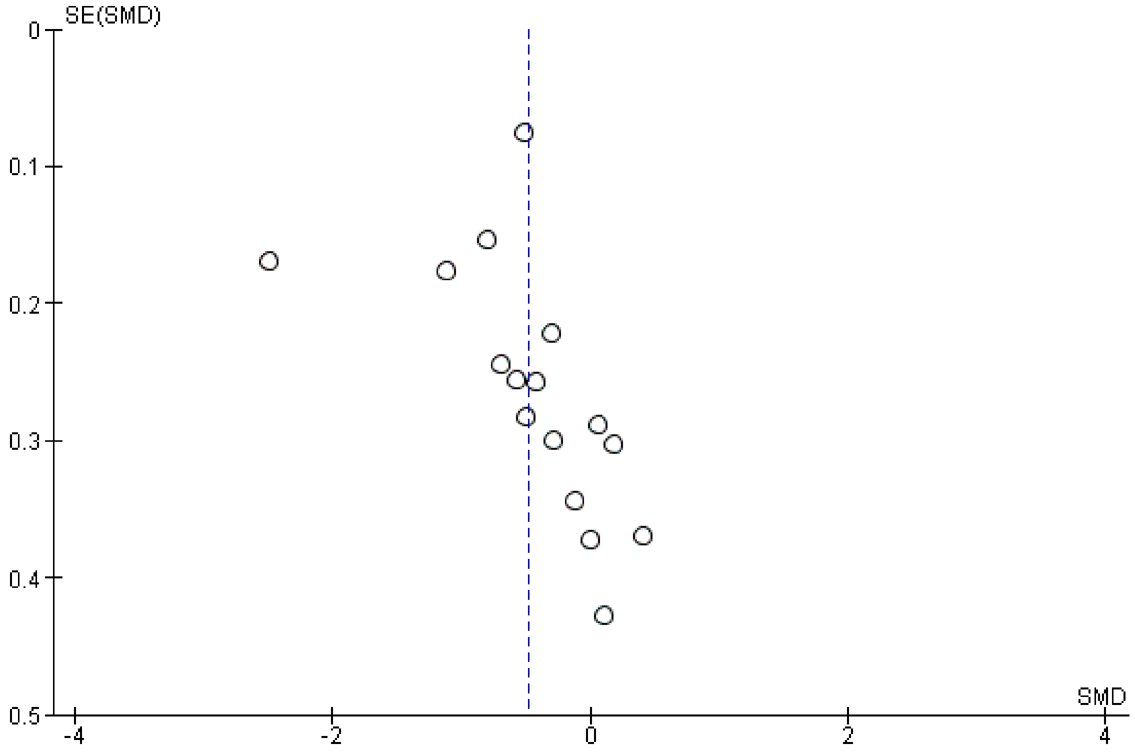

Supplementary Figure S55. Funnel plot of AST

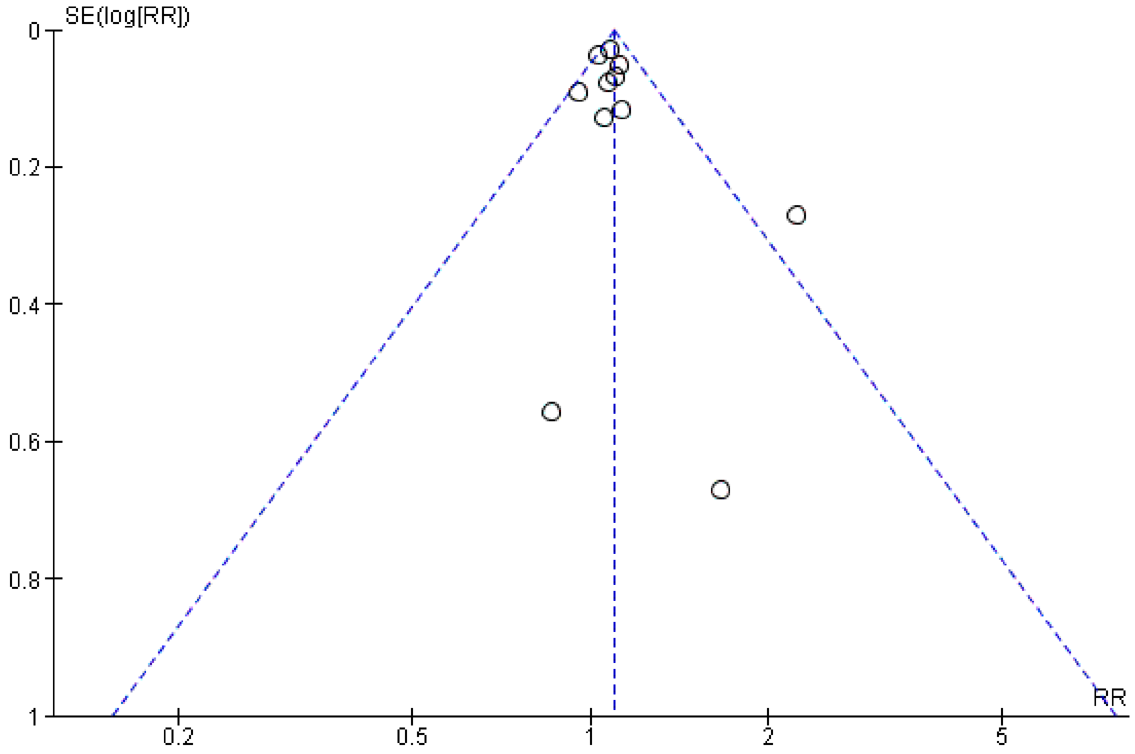

Supplementary Figure S56. Funnel plot of Adverse events.

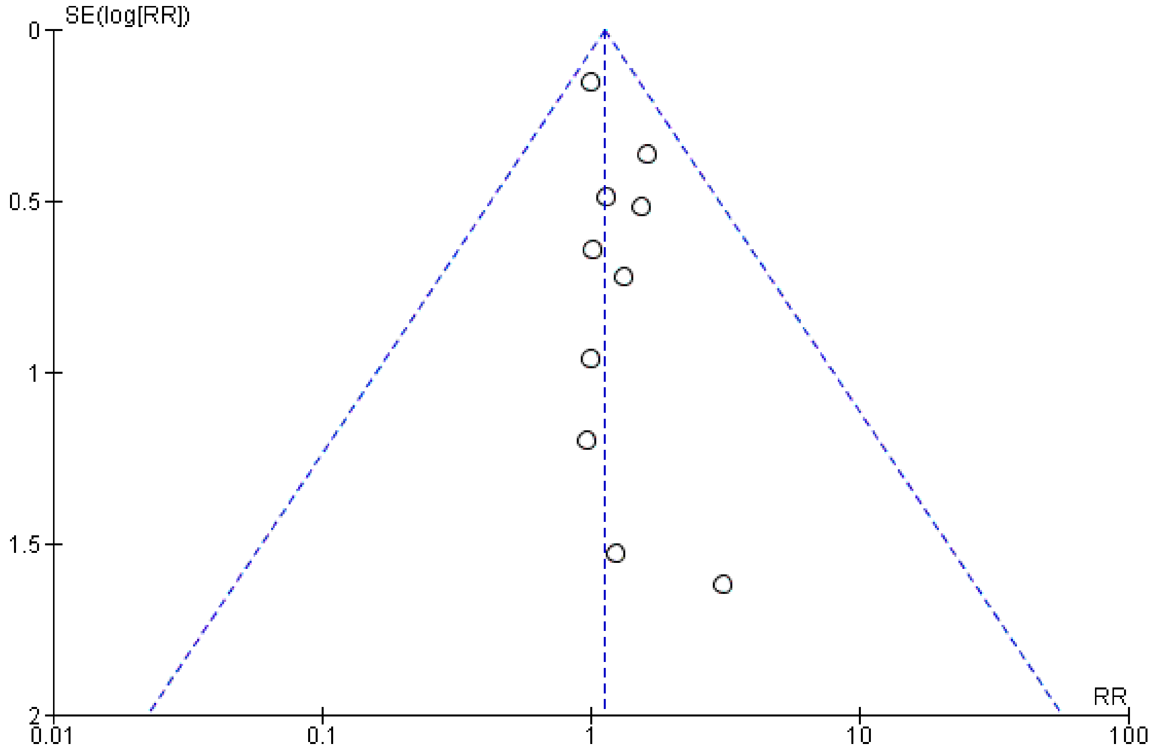

Supplementary Figure S57. Funnel plot of serious adverse events.

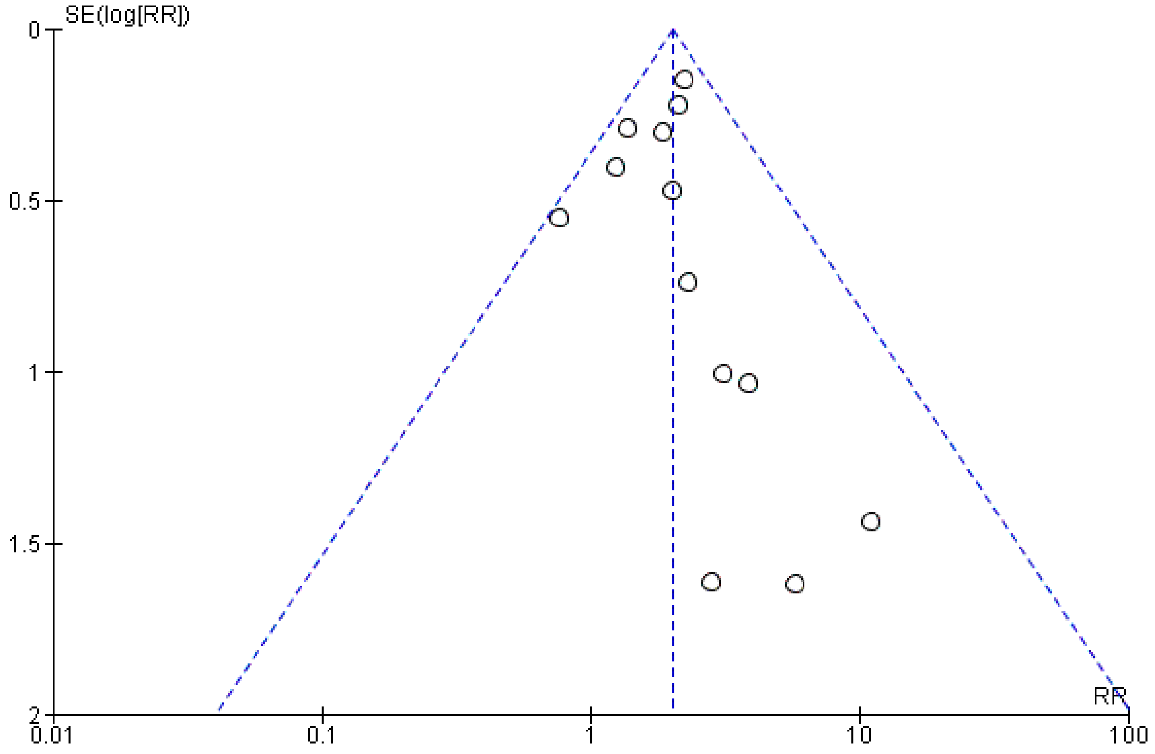

Supplementary Figure S58. Funnel plot of Diarrhea.

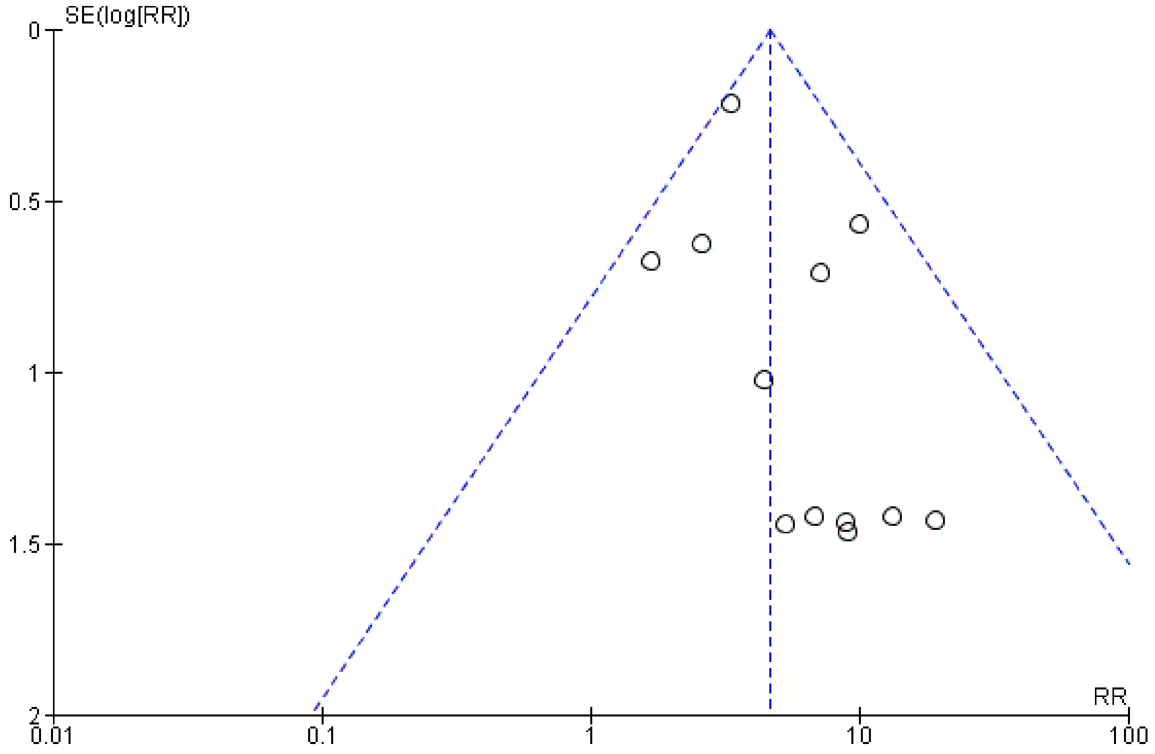

Supplementary Figure S59. Funnel plot of Vomiting.

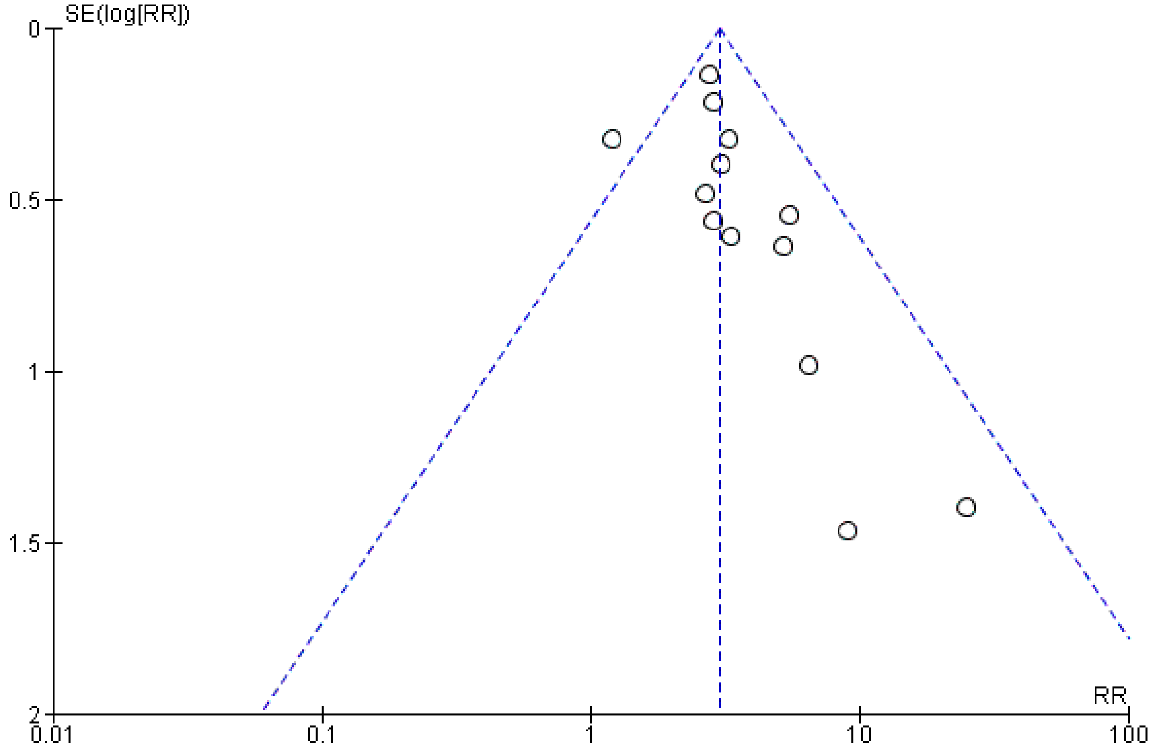

Supplementary Figure S60. Funnel plot of nausea.
